# Supplementary material for: You Can Teach Every Patient: A Health Literacy and Clear Communication Curriculum for Pediatric Clerkship Students
Source: MedEdPORTAL. 2021 Jan 22;17:11086. doi: 10.15766/mep_2374-8265.11086 (PMC7821440; doi:10.15766/mep_2374-8265.11086)
Supplement: Supplementary file 1 — HLCC Didactic PowerPoint.pptxWorkshop PowerPoint.pptxCTEP Card.docxVideo for Critique.m4vClear Language Cases Students.docxClear Language Cases Instructors Guide.docxTeach-back Cases Students.docxTeach-back Cases Instructors Guide.docxPicture Cases Students.docxPicture Cases Instructors Guide.docxCTEP Cases Students.docxCTEP Cases Instructors Guide.docxCommunication Checklist.docxStudent Survey.docx [file mep_2374-8265.11086-s001.zip › _Educational_Summary_Report_11086.pdf]

MedEdPORTAL® The AAMC Journal of Teaching and Learning Resources

Original Publication Open Access

You Can Teach Every Patient: A Health Literacy and Clear Communication Curriculum for Pediatric Clerkship Students

Emily Spengler, MD*, Miriam Schechter, MD, Paulo Pina, MD, MPH, Hai Jung Helen Rhim, MD, MPH

*Corresponding author: emilyspengler@gmail.com

Abstract

Introduction: Poor health literacy has a negative impact on various health care outcomes. Medical schools are not consistently providing health literacy training; when they do, they overly rely on didactics. **Methods:** Our curriculum for third-year pediatric clerkship students taught principles of health literacy and evidence-supported clear communication strategies. Communication skills were structured on a novel mnemonic: CTEP (clear language, teach-back, effectively encouraging questions, and pictures). The curriculum included a 30-minute didactic, followed 1-2 weeks later by a 90-minute interactive workshop. All 188 clerkship students attended the didactic lecture; approximately half (90) attended the follow-up workshop. All students completed a formative objective structured clinical encounter. Standardized patients then evaluated students' use of the four clear communication skills. Students completed a survey to assess confidence, knowledge, and use of the skills. **Results:** Compared to the didactic-only group, students in the didactic + workshop group more frequently used teach-back (53% vs. 27%, $p < .01$) and pictures (46% vs. 10%, $p < .01$). In addition, the didactic + workshop group had improved recall, self-reported use, and comfort with the skills. The didactic + workshop group solicited questions from the standardized patient less often, and there was no difference in use of clear language between the two groups. **Discussion:** An interactive curriculum in health literacy and clear communication for pediatric clerkship students was superior to a didactic alone. Optimizing instructional methods for health literacy skills can help future physicians properly communicate with their patients to improve health outcomes.

Keywords
Health Literacy, Communication Skills, Curriculum Development, Pediatrics

Educational Objectives

By the end of this activity, learners will be able to:

1. Discuss the best health literacy–informed communication skills to use with all patients.
2. Demonstrate clear language, teach-back, open-ended questions, and use of pictures during a simulated pediatric patient encounter.
3. Incorporate four specific clear communication skills into patient care.

Citation:
Spengler E, Schechter M, Pina P, Rhim HH. You can teach every patient: a health literacy and clear communication curriculum for pediatric clerkship students. *MedEdPORTAL*. 2021;17:11086.
https://doi.org/10.15766/mep_2374-8265.11086

Introduction

Health literacy has been defined by the Health and Medicine Division (HMD) of the National Academy of Sciences as "the degree to which individuals have the capacity to obtain, process, and understand basic information and services needed to make appropriate health decisions."[1] The fact that approximately one-third of US adults have limited health literacy[2] has far-reaching consequences for health outcomes, health care costs, and medication errors. While the HMD recommended health literacy skills training for professional skills in 2004,[1] as of a 2010 survey of US allopathic schools (with a 47% response rate), only 72% had health literacy curricula.[3]

While health literacy curricula are not yet universally implemented, there has been a recent increase in published curricula in this area.[4-13] There are robust and effective health literacy curricula for medical students,[4,8-10] residents,[6,7,11,12] attendings,[5,12] and multidisciplinary fields.[5] These published resources mostly utilize interactive workshops to teach health

Copyright © 2021 Spengler et al. This is an open-access publication distributed under the terms of the Creative Commons Attribution license.

literacy skills, but in practice, there is an overreliance on didactics. For example, in a 2010 survey of medical school health literacy curricula, the most common curricular method was didactics (84%), while fewer used workshops (46%) or standardized patients (SPs; 57%).[3] Few studies have compared efficacy of didactics versus other types of educational methods in this area.

Past health literacy curricula published in *MedEdPORTAL* have focused on adult care.[4,5,12,13] There are limited published curricula for teaching health literacy and clear communication skills when caring for pediatric patients and teaching parents and caregivers.

To fill this gap, we designed a curriculum for pediatric clerkship students to learn about health literacy and clear communication skills, with didactics, taped simulated patient encounters with feedback, and small-group role-plays as the primary instructional methods. Students also learned a novel mnemonic to encourage repeated practice of four core communication skills in the workshop and future clinical work. The curriculum was informed by the theory of deliberate practice, in which learners repeatedly engage in practice of skills in order to gain mastery of them.[14]

The intended learner for this curriculum is a clerkship student. We chose this learner because these students are early in the process of establishing their clinical routines and practice. It is important to lay a groundwork early for incorporating strong, evidence-based communication skills into future practice. Facilitators should preferably have experience in clinical medicine and be familiar with key concepts in health literacy and clear communication skills.

We evaluated this curriculum by giving different curricular elements to two separate groups of students and comparing performance on a formative, low-stakes objective structured clinical encounter (OSCE). One group of students attended just a didactic, while the other group attended a didactic and participated in an additional workshop. This curriculum adds the following to the growing body of knowledge of health literacy curricula: (1) an easy-to-implement curriculum tailored to pediatric clerkship students (and easily adapted for pediatric practitioners) and (2) a unique between-groups design to evaluate the optimal curricular method for teaching these skills.

Methods

A mini-curriculum focused on health literacy and clear communication skills was added to the third-year pediatrics clerkship. Review of the medical school curriculum with the assistant dean for medical education found that students had no mandatory prior medical school experience in these topics. No prerequisite learning was required for the curriculum. The curriculum focused only on evidence-based or consensus-supported clear communication techniques.[2,15,16] As directed by the Agency for Healthcare Research and Quality,[2] the curriculum used a Universal Precautions approach: Students were taught that because one never knows who has poor health literacy, clear communication skills should always be used.

At clerkship orientation, all students attended a 30-minute didactic lecture about health literacy and four clear communication skills (Appendix A). The didactic included a background on health literacy, featuring a definition, health literacy's prevalence in the United States, and the impact on clinical care. The second half of the didactic was dedicated to concrete evidence-based or consensus-supported clear communication skills. Four of these skills were introduced, along with a mnemonic we developed: Can Teach Every Patient (CTEP), C standing for clear language, T for teach-back, E for effectively encourage patient questions (using an open-ended technique), and P for pictures.

About 1-2 weeks after the didactic, approximately half of the students participated in a required 90-minute small-group skills workshop on the same topics. Only half the students participated in the workshop to allow for a between-groups comparison between the workshop group and the group that had just the didactic instruction. Workshop group size was approximately five to eight students, with the same facilitator for each group. The workshop included role-plays with feedback, video critiques, and small-group discussions, along with a PowerPoint (Appendix B) to guide the facilitator. At the beginning of the workshop, students were given CTEP cards with the four clear communication skills (Appendix C). Students were asked to refer to the card during the workshop and could keep it for future use, but no specific instructions were given about when and how to use this card in the future. Students watched a video of a simulated patient encounter (Appendix D) and critiqued the communication skills using the CTEP framework. Then, they reviewed key concepts from the health literacy didactic, with time to discuss their importance and reflect on personal experiences with health literacy.

The remaining time in the workshop was dedicated to skills practice. We practiced or reviewed the four skills in the following order:

1. Clear language: The facilitator gave each student one medical case scenario (Appendix E). Students took turns

Copyright © 2021 Spengler et al. This is an open-access publication distributed under the terms of the Creative Commons Attribution license.

explaining their case in clear language to the group, and then, the group gave the student feedback about their performance. The facilitator used the Clear Language Cases Instructors Guide (Appendix F) for this exercise.

2. Teach-back technique: In this role-play exercise, students paired off and practiced the teach-back technique. First, one pair demonstrated for the group, followed by group feedback. Then, all the pairs practiced. There were five sample cases (Appendix G), so the instructor chose one case for the group demonstration, then gave each pair two different cases. The pair role-played the cases with one student taking the provider role and the other the patient/caregiver role. The provider explained the case to the patient/caregiver and then elicited teach-back from the patient/caregiver. Students reversed roles for the second case. The facilitator used the Teach-Back Cases Instructors Guide (Appendix H) for this exercise.
3. Effectively encouraging questions: The facilitator taught use of an open-ended technique for encouraging patient questions (saying, “What questions do you have?” instead of “Do you have any questions?”). In each subsequent role-play, the facilitator reminded the students to use an open-ended approach for eliciting patient questions.
4. Pictures: As a group, students discussed ways to use pictures in clinical care. There were three simple clinical instructions (Appendix I) that students volunteered to draw on the board while others guessed the clinical instruction. The facilitator used the Picture Cases Instructors Guide (Appendix J) for this exercise.
5. CTEP: As a final role-play exercise, students again split into pairs. This exercise was structured the same as the teach-back exercise. First, one pair demonstrated for the group, followed by group feedback. Then, all the pairs practiced. There were five sample cases (Appendix K), so the instructor chose one case for the group demonstration, then gave each pair two different cases. The pair role-played the cases with one student taking the provider role and the other the patient/caregiver role. The provider explained the case to the patient/caregiver using all four techniques (CTEP). Students then reversed roles for the second case. They were encouraged to take out a pen and paper and incorporate pictures into their teaching. The facilitator used the CTEP Cases Instructors Guide (Appendix L) for this exercise.

At the end, the facilitator summarized key points. Students each shared one thing they would take away from the workshop and/or change in their future clinical practice.

The estimated time for each section of the workshop was as follows:

- Video/introduction: 15 minutes.
- Clear language (group practice): 20 minutes.
- Teach-back (group observing a pair with group feedback, then pairing and practicing): 20 minutes.
- Pictures (group practice): 5 minutes.
- CTEP practice (group observing a pair with group feedback, then pairing and practicing): 30 minutes.
- Wrap-up: 5 minutes.

Materials used in the workshop included the following:

- Large display screen and computer to project the PowerPoint onto the screen.
- Whiteboard.
- Handouts with case scenarios for instructors and students (Appendices E-L).

Curriculum Evaluation

Our objectives included improved knowledge, skills, and attitudes. To evaluate the effectiveness of the curriculum, we conducted a between-groups design in the implementation year of the program. Students were divided into two groups based on their clinical sites. These sites were chosen based on convenience of workshop administration and to create a cohort of students comprising approximately half of the class. To test the effects of didactic versus didactic + workshop, all students attended the didactic lecture, but the 90-minute skills workshop was given to only half the students.

All of the students—didactic-only and didactic + workshop—participated in an educational day midway through the clerkship, about 2-3 weeks after the didactic and workshop. On that day, we tested their knowledge and attitudes (learning) through a survey and their use of the skills (behavior) through self-reported use and performance on an OSCE.

To assess behavioral changes, a brief 5-minute counseling task with an SP in a simulation center was added to an existing pediatric clerkship OSCE case, with separate educational goals. The addition to the case challenged all students to explain fever and antipyretic dosing to an SP acting as the parent of a sick infant in the emergency department setting. The SP evaluated the students based on a checklist of how many of the four clear communication skills they used (Appendix M). The checklist was adapted from a tool developed by Green, Gonzaga, Cohen, and Spagnoletti to evaluate a health literacy and clear communication curriculum for internal medicine residents.[6] All of the SPs were

Copyright © 2021 Spengler et al. This is an open-access publication distributed under the terms of the Creative Commons Attribution license.

from a professional agency. They were trained by the same faculty member on the OSCE case and use of the evaluation tool. It is important to note that the checklist can be used in any counseling OSCE to evaluate health literacy and clear communication. Therefore, the specific addition to our OSCE, focusing on fever and antipyretic dosing, is not included with this publication.

To evaluate changes in knowledge and attitudes, students completed a survey about knowledge and use of skills and a retrospective pre-post question about comfort with the skills (Appendix N). Edits were made to the survey after the start of the study, so only students in the final two-thirds of the year completed the final version of the survey. Only data from the final version of the survey were analyzed.

Results

The curriculum was successfully implemented with third-year pediatric clerkship students. A total of 188 students participated. All students attended the didactic, and 90 attended the workshop. Data include survey responses from 80 students and the SP OSCE performance ratings for all 188 students.

Survey results are summarized in Table 1. Students from the workshop group could recall a greater number of the four primary clear communication skills compared to the didactic-only group (2.4 vs. 0.4, $p < .01$) and on a retrospective pre-post survey had improved ratings of comfort using the skills after the curriculum (1.4 vs. 0.7, $p < .01$). Of the workshop group, 96% reported using the skills during the clerkship, compared to 64% in the didactic-only group ($p < .01$).

The OSCE performance results are summarized in Table 2. Students in the workshop group used teach-back (53% vs. 27%, $p < .01$) and pictures (46% vs. 10%, $p < .01$) at a greater rate. There was no significant group difference in use of clear language (88% vs. 84%, $p = .38$). The didactic-only group was more likely to ask the SP for questions (92% vs. 78%, $p < .01$), but among those who asked for questions, there was a nonsignificant trend towards greater use of the preferred open-ended method in the workshop group (25% vs. 15%, $p = .13$).

Table 1. Postcurriculum Survey Results (*N* = 80)

Survey Measure	Didactic Group	Didactic + Workshop Group	*p*
Average number of communication skills recalled[a]	0.4	2.4	<.01
Difference in retrospective pre/post comfort using skills[b]	0.7	1.4	<.01
Percentage of students who used CTEP skills in the clerkship	64%	96%	<.01

[a]Out of four communication skills taught.
[b]Rated on a 5-point scale (1 = *low*, 5 = *high*).

Table 2. Objective Structured Clinical Encounter Data

Communication Skill	Didactic Group	Didactic + Workshop Group	*p*
Clear language use	84%	88%	.38
Teach-back use	27%	53%	<.01
Asked standardized patient for questions	92%	78%	<.01
Used open-ended approach to ask standardized patient for questions	15%	25%	.13
Use of pictures	10%	46%	<.01

In summary, the didactic + workshop curriculum led to greater use of two of the four clear communication skills—teach-back method and pictures—in an OSCE. Students in the didactic + workshop group could name more of the key communication skills, had greater comfort using the skills, and had higher rates of self-reported use of the skills in the clerkship.

Discussion

Improving communication skills involves a conscious change in standard clinical practice, one that it seems cannot be met by a simple didactic session. The curriculum presented here showed statistically significant improvements in knowledge and use of these skills when students received instruction at two discrete encounters and presented in an interactive manner, as compared to a single didactic session. Furthermore, this health literacy curriculum was easy to administer and one of few published for care of pediatric patients.

The curriculum was based on the theory of deliberate practice. Ericsson defined the conditions associated with improved performance of a skill: Learners should be (1) given a task with a well-defined goal, (2) motivated to improve, (3) provided with feedback, and (4) offered the opportunity for repetition and refinement of their performance.[14] The workshop included context and meaning for the skills, and learners had an opportunity for repeated, purposeful practice of the skills, along with time for reflection and feedback. This allowed learners to start down the road toward mastery of these skills and to incorporate them into their clinical practice.

This curriculum was studied with pediatric clerkship students. However, it is easily adaptable for use across the educational continuum (in pediatric residents and attendings) or across disciplines (nurses, medical assistants, and advanced practitioners). It can be easily adapted to other clerkships or residency types by adapting the cases. We recommend early introduction of these skills—at the time learners are developing

Copyright © 2021 Spengler et al. This is an open-access publication distributed under the terms of the Creative Commons Attribution license.

clinical scripts and habits—to best incorporate them into long-term clinical practice.

The greatest challenge for implementation of the curriculum was faculty engagement. While the didactic was easy to implement, the workshop was studied in small groups of only five to eight students and so required a significant amount of faculty time. In the year after this curriculum was studied, the workshop was also implemented with a large group of about 30 students, with breakouts into smaller groups for role-plays. It was always well received, with good engagement, and required only two to three faculty to be successful.

While this study shows statistically significant differences in the didactic + workshop group compared to the didactic-only group, there are some areas of similar results between the two groups worth noting. Both groups had similar SP ratings on use of clear language (see Table 2). Despite prompts explaining what jargon is, as well as training, and retraining when necessary, of SPs, our tool was an imperfect measure of clear language use. A better assessment of language use, such as transcripts and/or multiple evaluators, would be preferable and yield richer results.

Another surprising finding was that students in the workshop group asked the SPs for questions less often than did the didactic group. It may have been that the workshop group more frequently used other skills, such as teach-back and pictures, and that in the context of a time-limited encounter like this OSCE, they simply ran out of time before asking the SP for questions. Alternatively, it is possible that the students in the didactic group were overly reliant on asking whether the SP had any questions to assess patient understanding, instead of using teach-back. However, when the students in the workshop group asked the SP for questions, the workshop group had a statistically insignificant trend toward using the open-ended approach ("What questions do you have?") more often than did the didactic group.

A limitation of the curriculum evaluation is that the two groups were not assigned randomly but rather based on clinical sites and convenience of administration of the workshop to half of the class. However, there is no reason to believe clinical site assignment in any way correlated with clear communication or health literacy skills. Another limitation is that our survey was not validated. However, the survey included a retrospective pre-post question, which has been shown to be a useful assessment of learning.[17]

Much work needs to be done in the field of health literacy and clear communication education. It will be hard to effect long-term, sustainable changes in clinical practice to address the needs of patients with low health literacy. Based on our experience, future curricular efforts must include repeated encounters with the material and interactive teaching methods. Future curricular evaluation methods can include evaluations by patients to better assess real-life application of the skills taught. This curriculum is an important start, but it cannot stand alone. As demanded by deliberate practice theory, these skills should be repeatedly reinforced across the educational curriculum for a sustained impact. Further work should be done to construct longitudinal curricula to address the needs of patients with low health literacy.

Appendices

A. HLCC Didactic PowerPoint.pptx
B. Workshop PowerPoint.pptx
C. CTEP Card.docx
D. Video for Critique.m4v
E. Clear Language Cases Students.docx
F. Clear Language Cases Instructors Guide.docx
G. Teach-back Cases Students.docx
H. Teach-back Cases Instructors Guide.docx
I. Picture Cases Students.docx
J. Picture Cases Instructors Guide.docx
K. CTEP Cases Students.docx
L. CTEP Cases Instructors Guide.docx
M. Communication Checklist.docx
N. Student Survey.docx

All appendices are peer reviewed as integral parts of the Original Publication.

Emily Spengler, MD: Assistant Professor, Department of Pediatrics, Drexel University College of Medicine and St. Christopher's Hospital for Children; ORCID: https://orcid.org/0000-0002-9642-0258

Miriam Schechter, MD: Associate Professor, Department of Pediatrics, Albert Einstein College of Medicine and the Children's Hospital at Montefiore

Paulo Pina, MD, MPH: Clinical Assistant Professor, Department of Pediatrics, New York University Grossman School of Medicine

Hai Jung Helen Rhim, MD, MPH: Assistant Professor, Department of Pediatrics, Albert Einstein College of Medicine and the Children's Hospital at Montefiore

Copyright © 2021 Spengler et al. This is an open-access publication distributed under the terms of the Creative Commons Attribution license.

Disclosures
None to report.

Funding/Support
This work was supported by the Albert Einstein College of Medicine Grants for Excellence in Medical Education.

Ethical Approval
Reported as not applicable.

References

1. Institute of Medicine. *Health Literacy: A Prescription to End Confusion*. National Academies Press; 2004.
2. Brega AG, Barnard J, Mabachi NM, et al. *AHRQ Health Literacy Universal Precautions Toolkit*. 2nd ed. Agency for Healthcare Research and Quality; 2015. AHRQ publication no. 15-0023-EF.
3. Coleman CA, Appy S. Health literacy teaching in US medical schools, 2010. *Fam Med*. 2012;44(7):504-507.
4. Zoberi K, Everard K, Deckert J, Frankenstein L. Health literacy for clerkship students. *MedEdPORTAL*. 2008;4:732. https://doi.org/10.15766/mep_2374-8265.732
5. Allenbaugh J, Spagnoletti CL, Rack L, Rubio D, Corbelli J. Health literacy and clear bedside communication: a curricular intervention for internal medicine physicians and medicine nurses. *MedEdPORTAL*. 2019;15:10795. https://doi.org/10.15766/mep_2374-8265.10795
6. Green JA, Gonzaga AM, Cohen ED, Spagnoletti CL. Addressing health literacy through clear health communication: a training program for internal medicine residents. *Patient Educ Couns*. 2014;95(1):76-82. https://doi.org/10.1016/j.pec.2014.01.004
7. Kripalani S, Osborn CY, Vaccarino V, Jacobson TA. Development and evaluation of a medication counseling workshop for physicians: can we improve on “take two pills and call me in the morning”? *Med Educ Online*. 2011;16(1):7133. https://doi.org/10.3402/meo.v16i0.7133
8. Coleman CA, Peterson-Perry S, Bumsted T. Long-term effects of a health literacy curriculum for medical students. *Fam Med*. 2016; 48(1):49-53. https://doi.org/10.22454/PRiMER.2017.703541
9. Marion GS, Hairston JM, Davis SW, Kirk JK. Using standardized patient assessments to evaluate a health literacy curriculum. *Fam Med*. 2018;50(1):52-57. https://doi.org/10.22454/FamMed.2018.539107
10. Saba GW, Chou CL, Satterfield J, et al. Teaching patient-centered communication skills: a telephone follow-up curriculum for medical students. *Med Educ Online*. 2014;19(1):22522. https://doi.org/10.3402/meo.v19.22522
11. Kripalani S, Jacobson KL, Brown S, Manning K, Rask KJ, Jacobson TA. Development and implementation of a health literacy training program for medical residents. *Med Educ Online*. 2006;11(1):4612. https://doi.org/10.3402/meo.v11i.4612
12. Limaye S, Hikoyeda N, Yeo G, MacClarence P, Baron AR. Geriatric health literacy workshop. *MedEdPORTAL*. 2013;9:9395. https://doi.org/10.15766/mep_2374-8265.9395
13. Howley L, Peterson C, Barrett E, Gazewood J, Wilson W, Holroyd S. Utilizing standardized patients to enhance health literacy communication skills. *MedEdPORTAL*. 2008;4:1101. https://doi.org/10.15766/mep_2374-8265.1101
14. Ericsson KA. Deliberate practice and acquisition of expert performance: a general overview. *Acad Emerg Med*. 2008; 15(11):988-994. https://doi.org/10.1111/j.1553-2712.2008.00227.x
15. Yin HS, Dreyer BP, van Schaick L, Foltin GL, Dinglas C, Mendelsohn AL. Randomized controlled trial of a pictogram-based intervention to reduce liquid medication dosing errors and improve adherence among caregivers of young children. *Arch Pediatr Adolesc Med*. 2008;162(9):814-822. https://doi.org/10.1001/archpedi.162.9.814
16. Schillinger D, Piette J, Grumbach K, et al. Closing the loop: physician communication with diabetic patients who have low health literacy. *Arch Intern Med*. 2003;163(1):83-90. https://doi.org/10.1001/archinte.163.1.83
17. Bhanji F, Gottesman R, de Grave W, Steinert Y, Winer LR. The retrospective pre–post: a practical method to evaluate learning from an educational program. *Acad Emerg Med*. 2012;19(2):189-194. https://doi.org/10.1111/j.1553-2712.2011.01270.x

Received: April 22, 2020
Accepted: October 20, 2020
Published: January 22, 2021

Copyright © 2021 Spengler et al. This is an open-access publication distributed under the terms of the Creative Commons Attribution license.
