# Supplementary material for: You Can Teach Every Patient: A Health Literacy and Clear Communication Curriculum for Pediatric Clerkship Students
Source: MedEdPORTAL. 2021 Jan 22;17:11086. doi: 10.15766/mep_2374-8265.11086 (PMC7821440; doi:10.15766/mep_2374-8265.11086)
Supplement: Supplementary file 1 — HLCC Didactic PowerPoint.pptxWorkshop PowerPoint.pptxCTEP Card.docxVideo for Critique.m4vClear Language Cases Students.docxClear Language Cases Instructors Guide.docxTeach-back Cases Students.docxTeach-back Cases Instructors Guide.docxPicture Cases Students.docxPicture Cases Instructors Guide.docxCTEP Cases Students.docxCTEP Cases Instructors Guide.docxCommunication Checklist.docxStudent Survey.docx [file mep_2374-8265.11086-s001.zip › A. HLCC Didactic PowerPoint.pptx]

## Slide 1
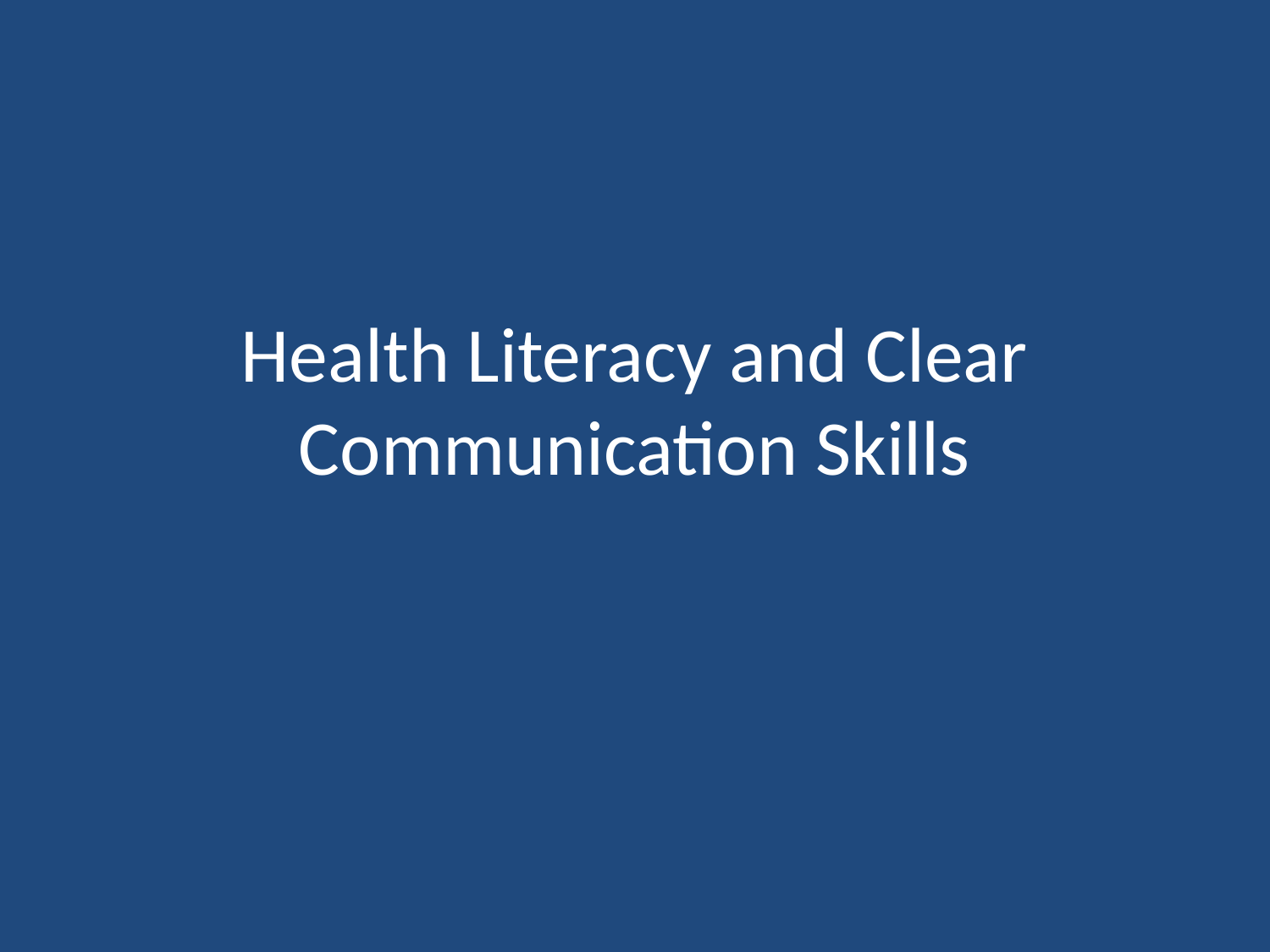

# Health Literacy and Clear Communication Skills

## Slide 2
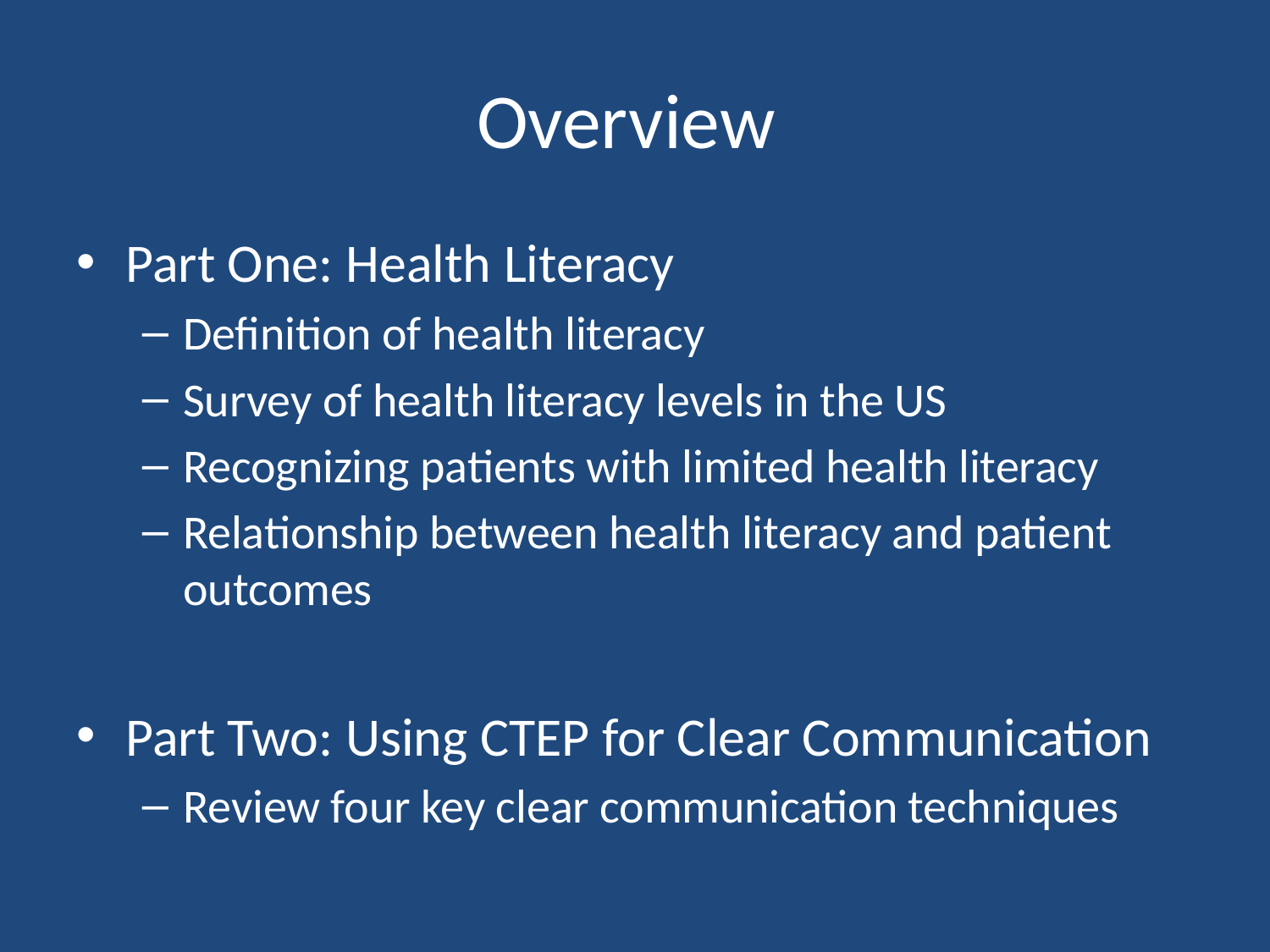

# Overview
Part One: Health Literacy
Definition of health literacy
Survey of health literacy levels in the US
Recognizing patients with limited health literacy
Relationship between health literacy and patient outcomes
Part Two: Using CTEP for Clear Communication
Review four key clear communication techniques

## Slide 3
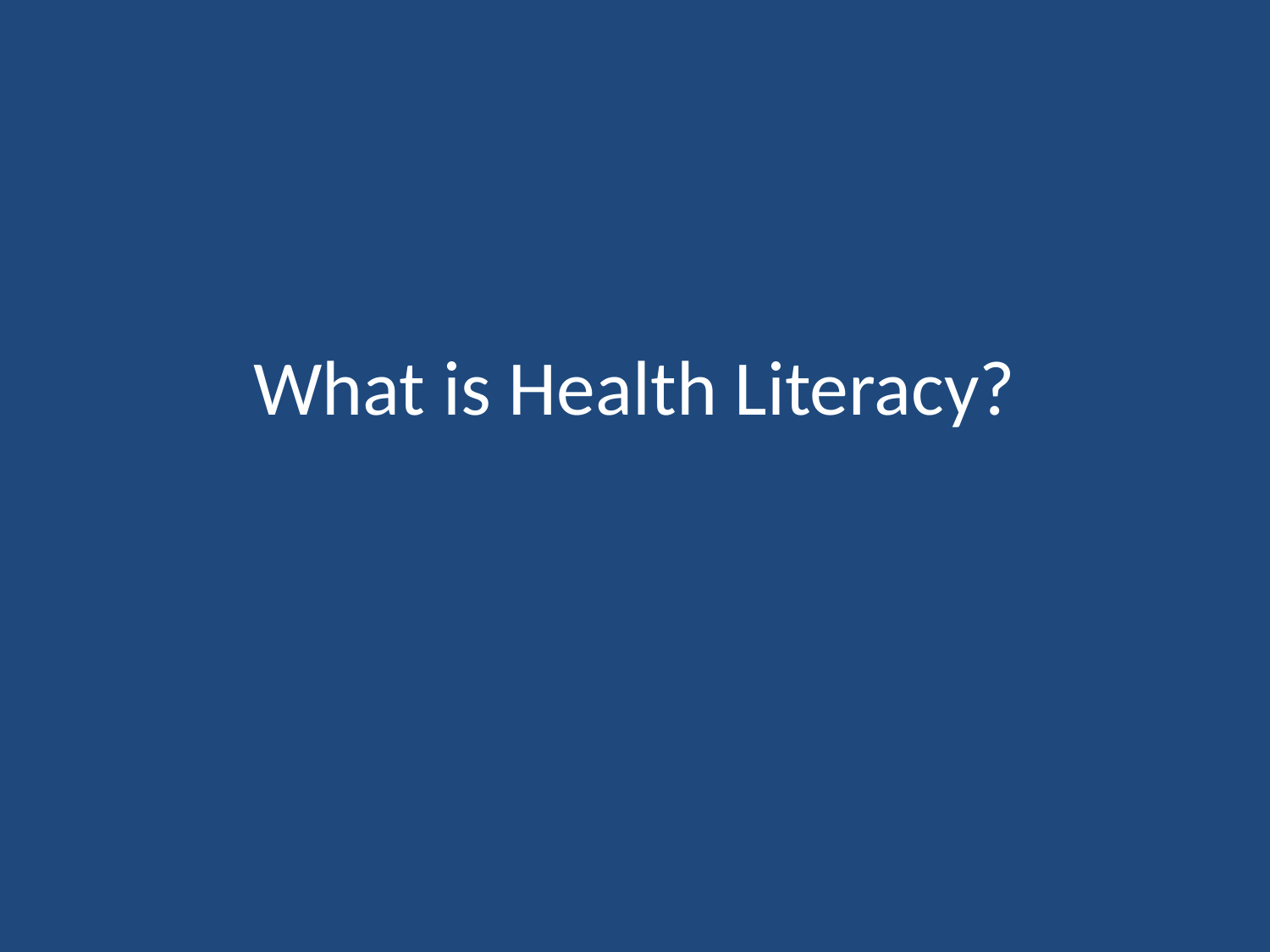

What is Health Literacy?

## Slide 4
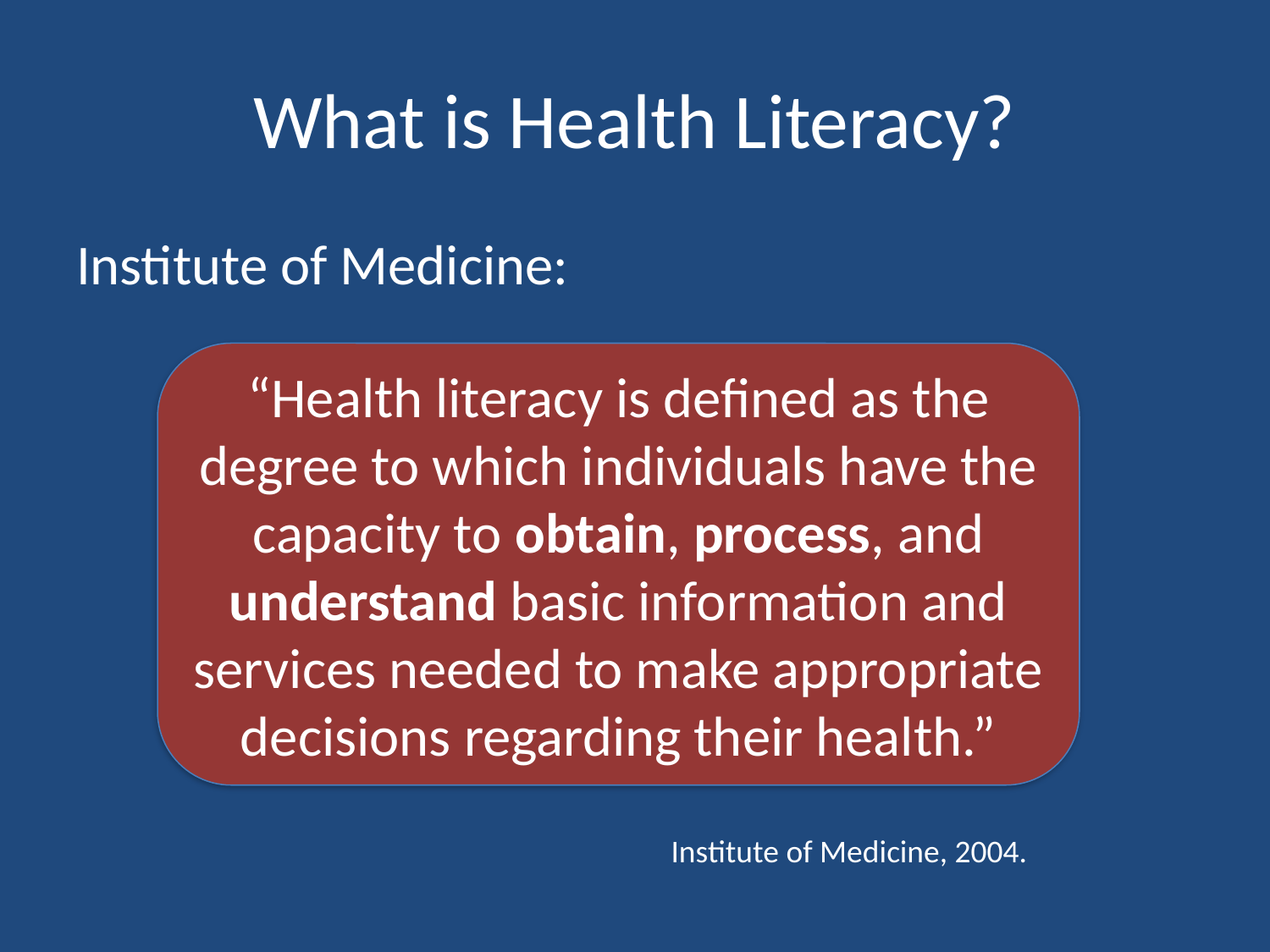

# What is Health Literacy?
Institute of Medicine:
“Health literacy is defined as the degree to which individuals have the capacity to obtain, process, and understand basic information and services needed to make appropriate decisions regarding their health.”
Institute of Medicine, 2004.

## Slide 5
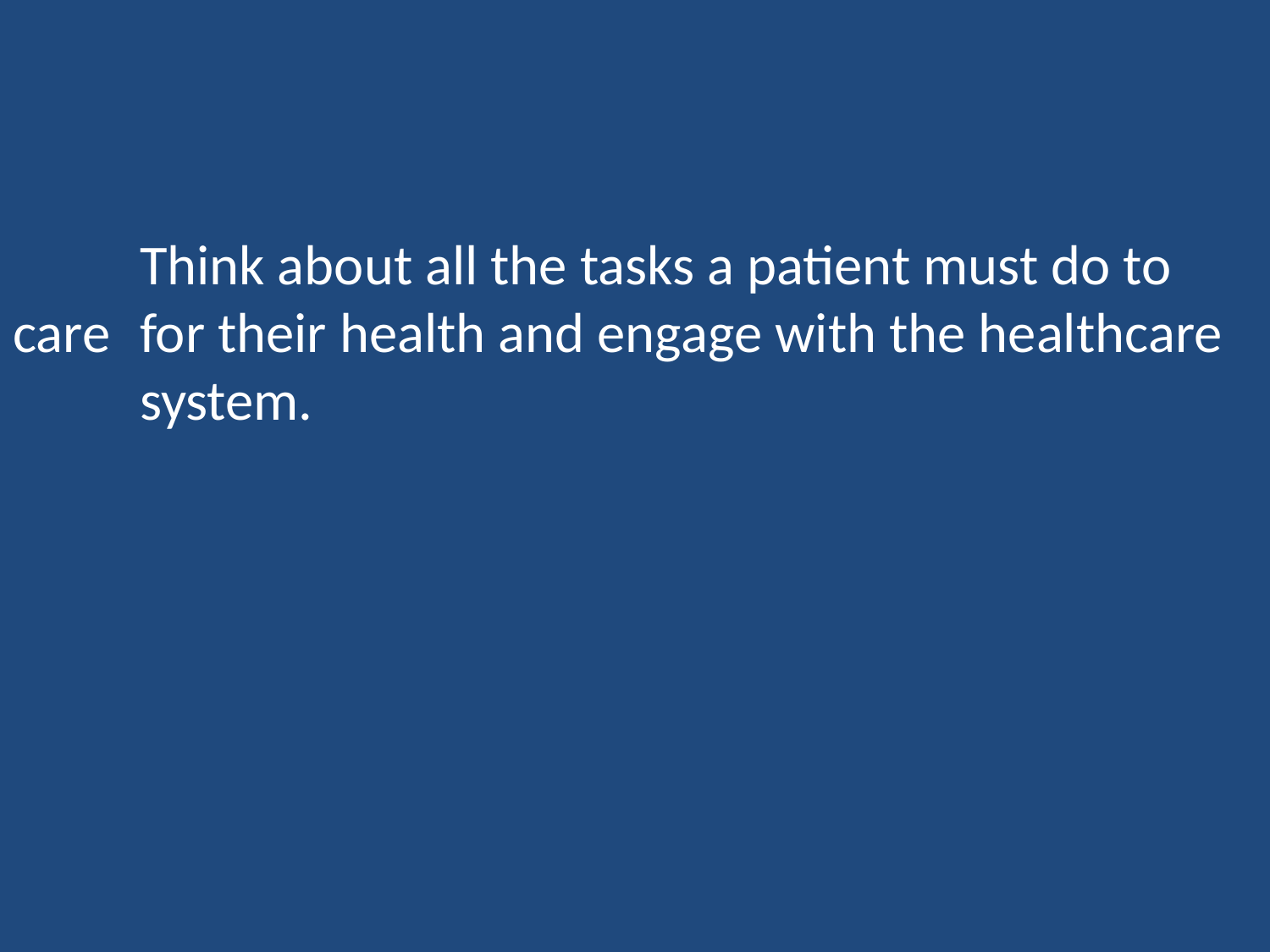

Think about all the tasks a patient must do to care 	for their health and engage with the healthcare 	system.

## Slide 6
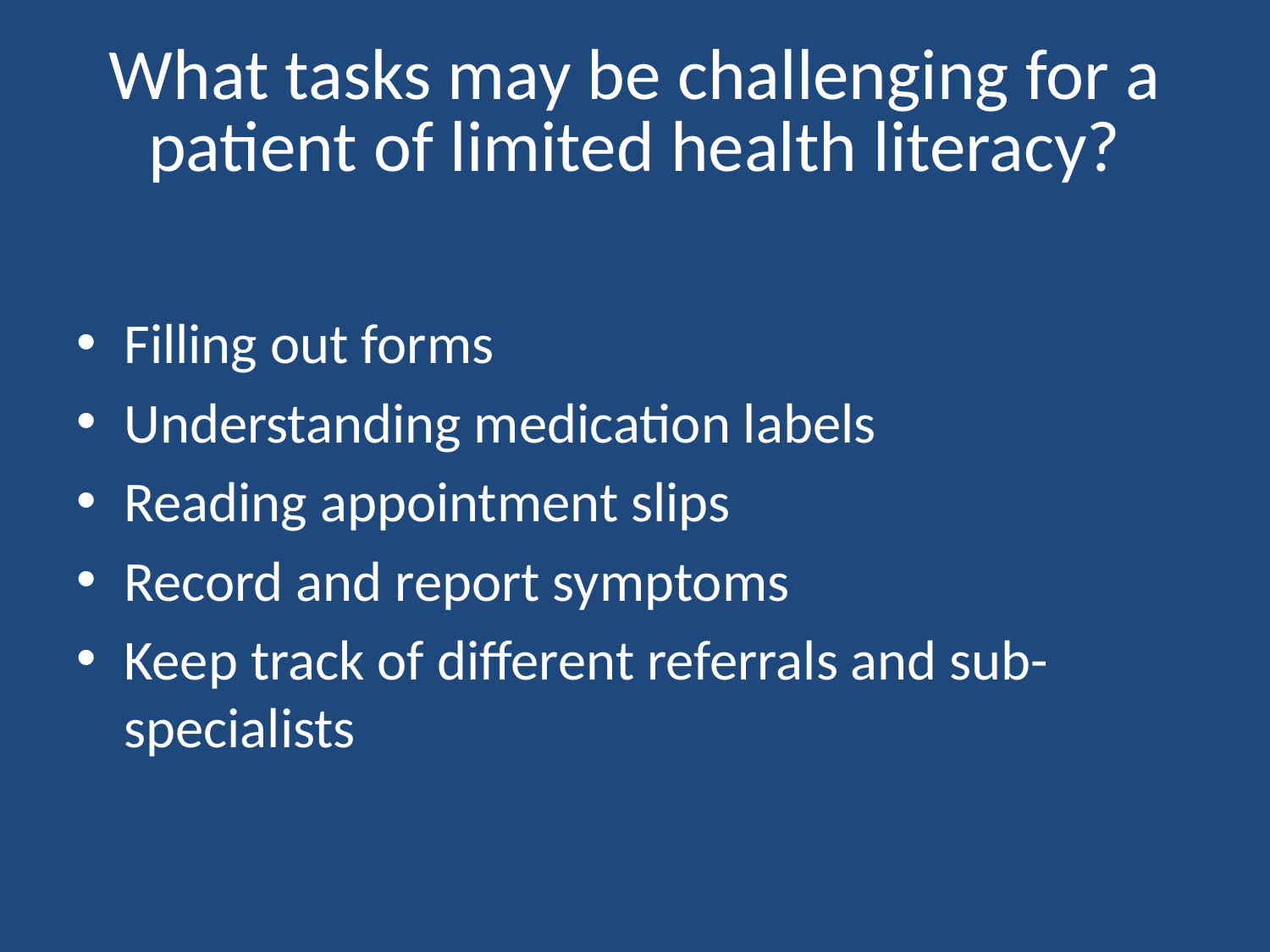

# What tasks may be challenging for a patient of limited health literacy?
Filling out forms
Understanding medication labels
Reading appointment slips
Record and report symptoms
Keep track of different referrals and sub-specialists

## Slide 7
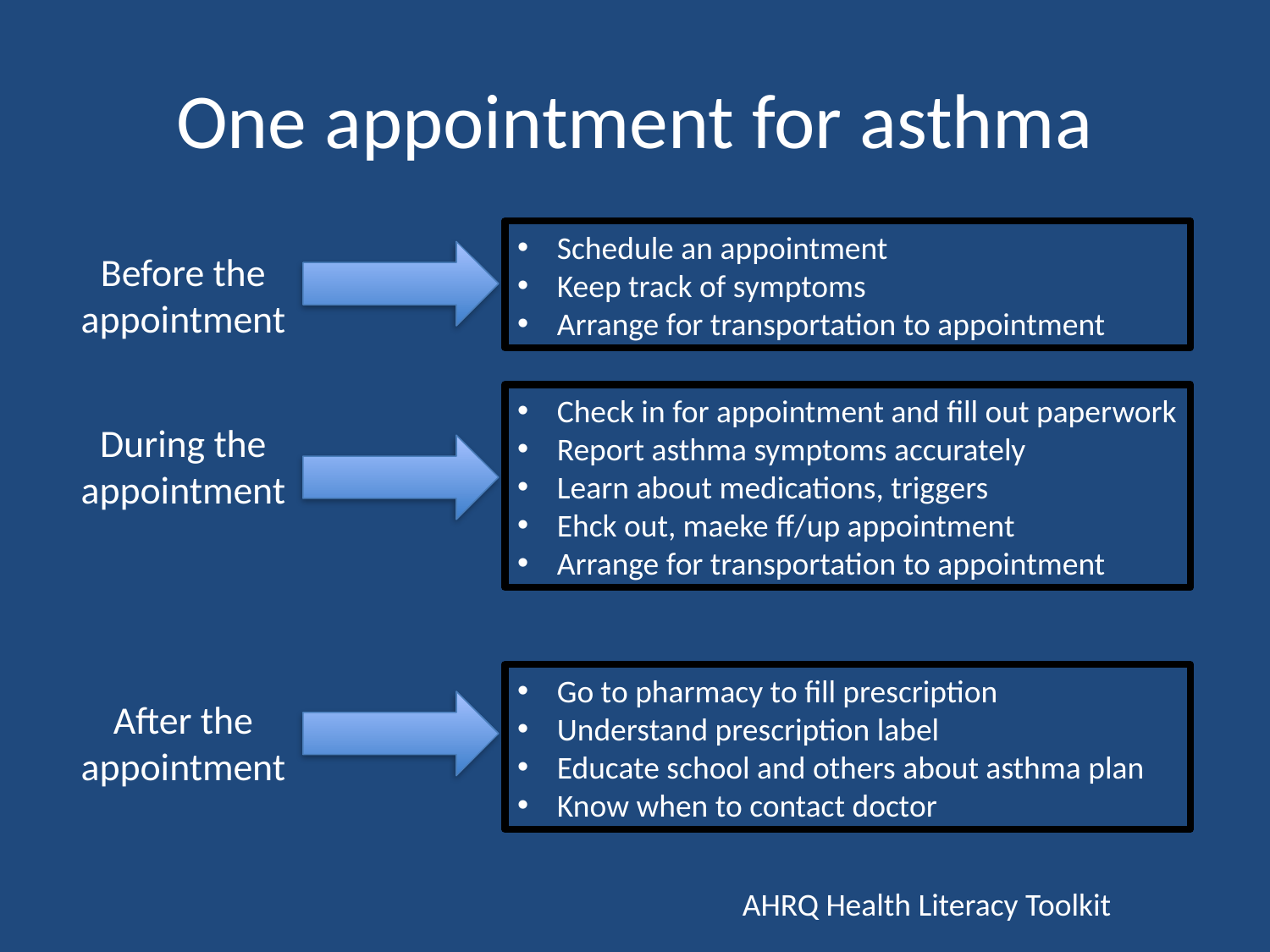

# One appointment for asthma
Schedule an appointment
Keep track of symptoms
Arrange for transportation to appointment
Before the appointment
Check in for appointment and fill out paperwork
Report asthma symptoms accurately
Learn about medications, triggers
Ehck out, maeke ff/up appointment
Arrange for transportation to appointment
During the appointment
Go to pharmacy to fill prescription
Understand prescription label
Educate school and others about asthma plan
Know when to contact doctor
After the appointment
AHRQ Health Literacy Toolkit

## Slide 8
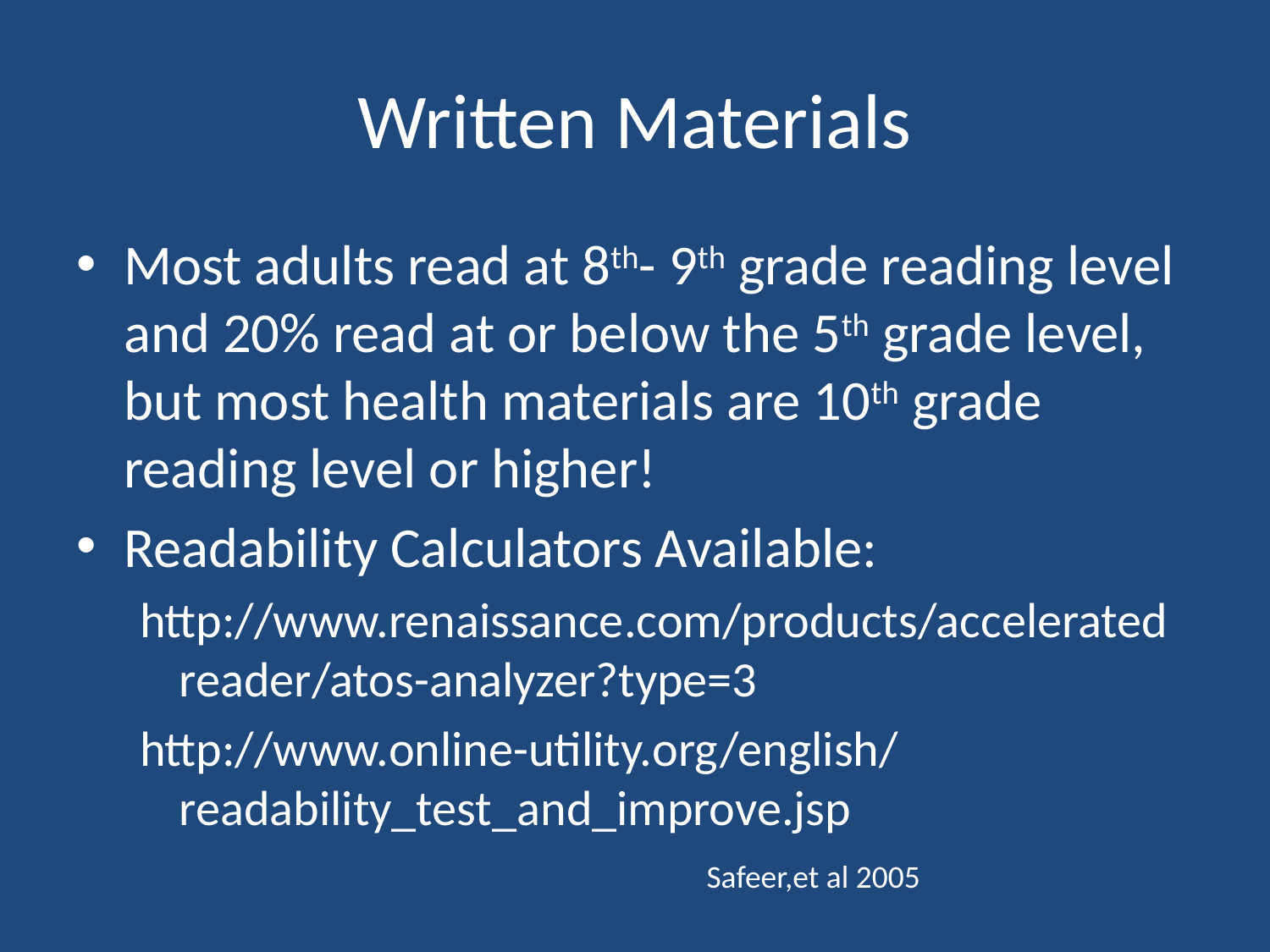

# Written Materials
Most adults read at 8th- 9th grade reading level and 20% read at or below the 5th grade level, but most health materials are 10th grade reading level or higher!
Readability Calculators Available:
http://www.renaissance.com/products/accelerated reader/atos-analyzer?type=3
http://www.online-utility.org/english/readability_test_and_improve.jsp
Safeer,et al 2005

## Slide 9
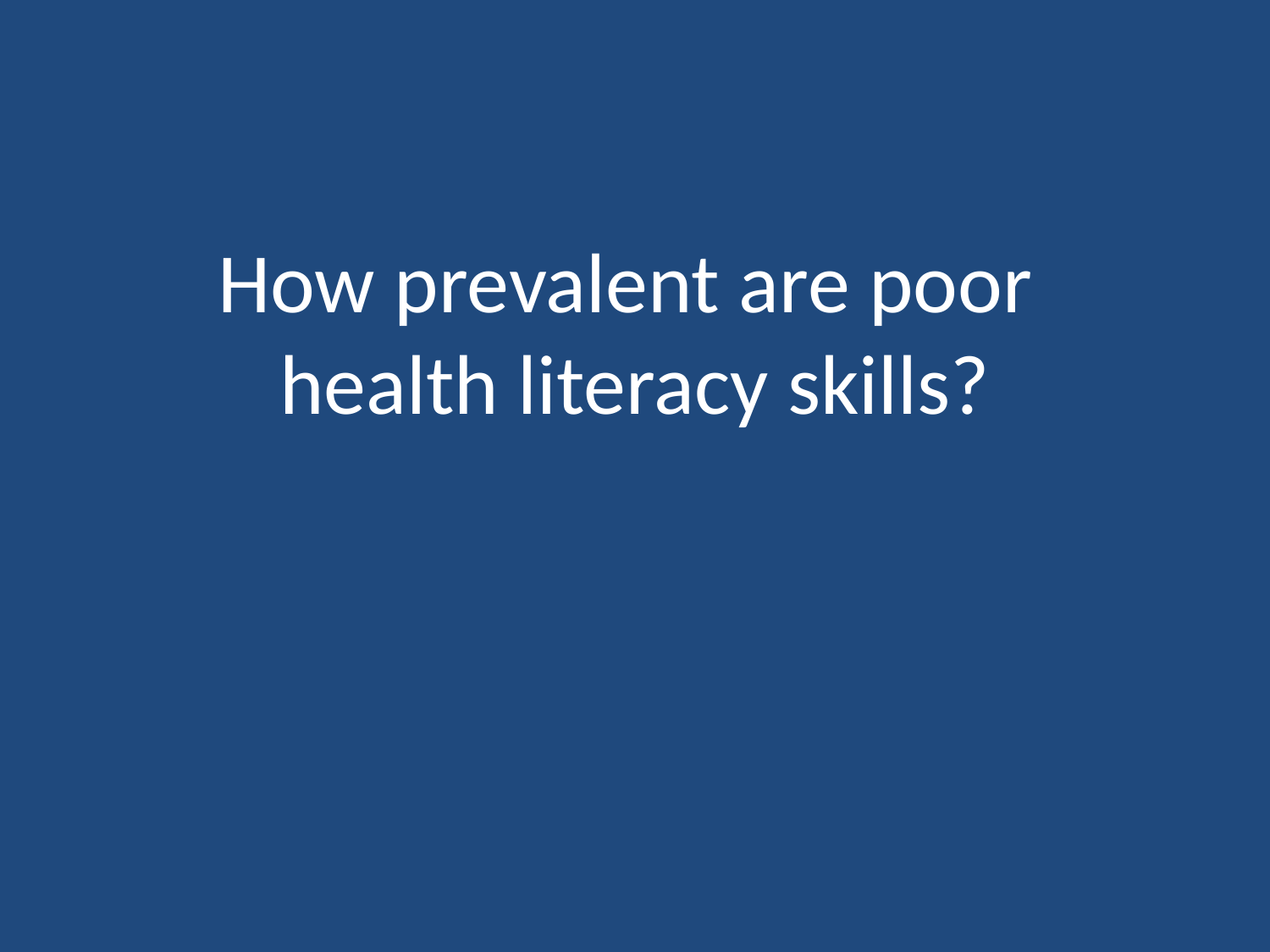

How prevalent are poor
health literacy skills?

## Slide 10
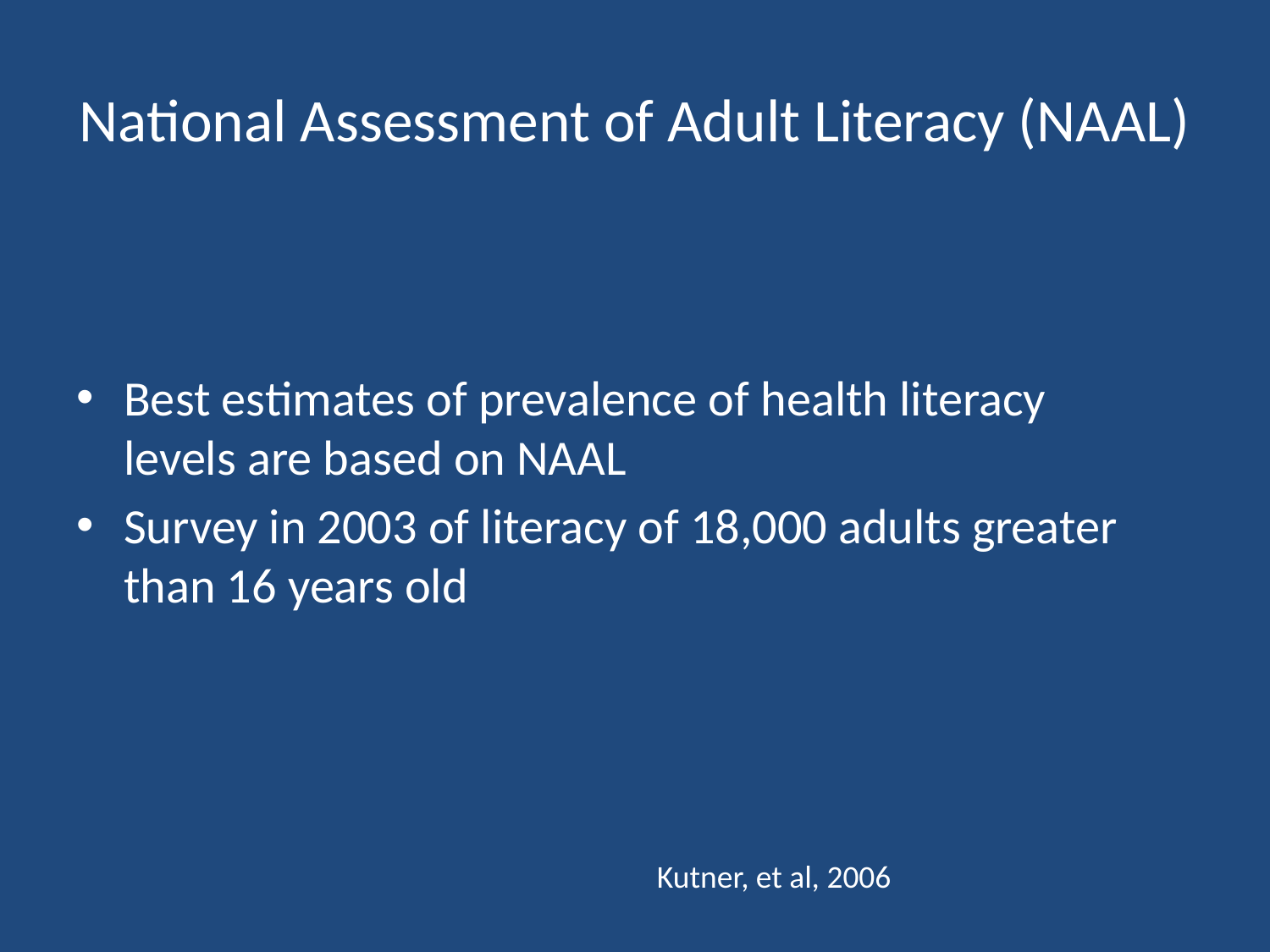

# National Assessment of Adult Literacy (NAAL)
Best estimates of prevalence of health literacy levels are based on NAAL
Survey in 2003 of literacy of 18,000 adults greater than 16 years old
Kutner, et al, 2006

## Slide 11
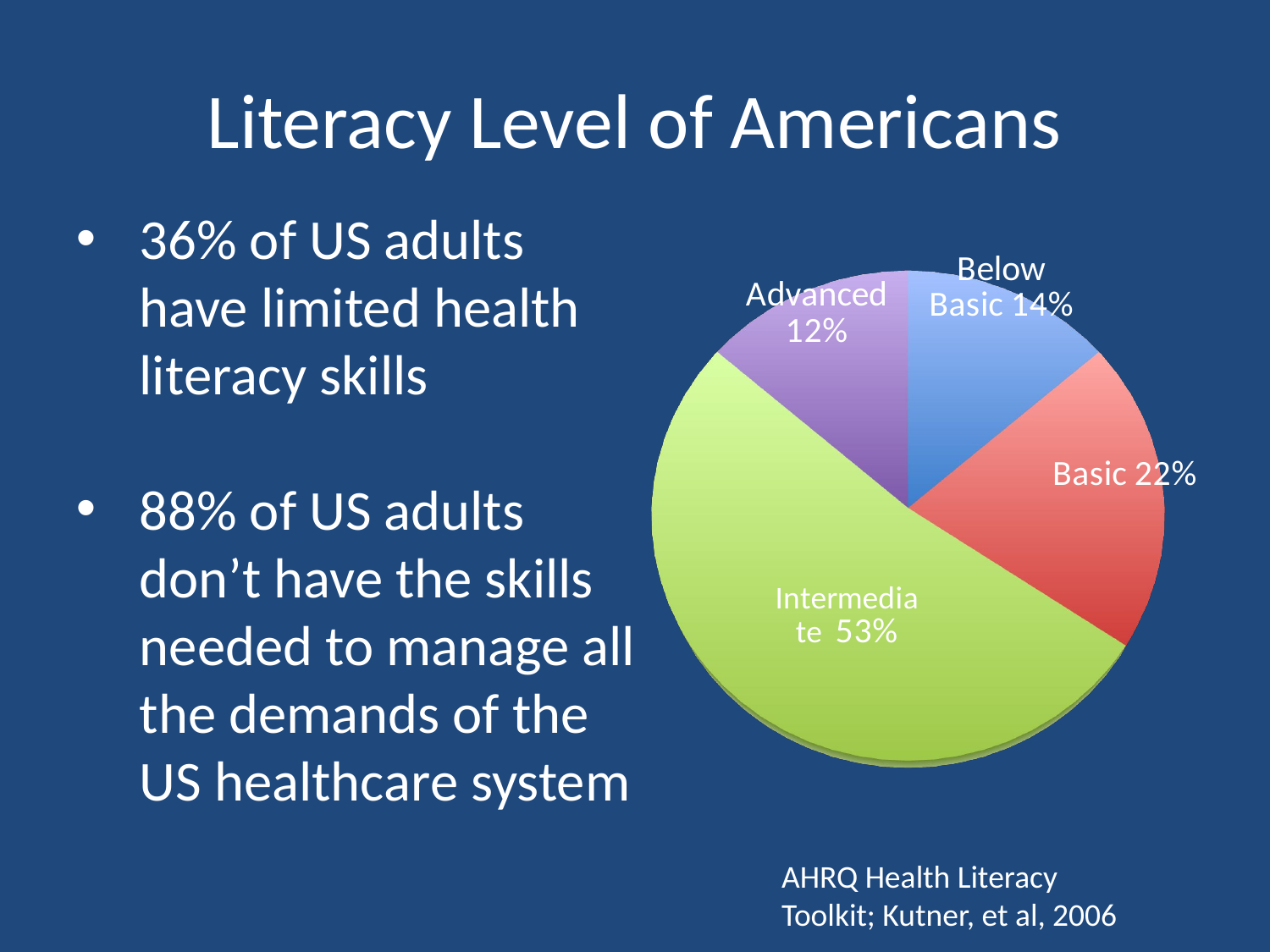

# Literacy Level of Americans
36% of US adults have limited health literacy skills
88% of US adults don’t have the skills needed to manage all the demands of the US healthcare system
[unsupported chart]
AHRQ Health Literacy Toolkit; Kutner, et al, 2006

## Slide 12
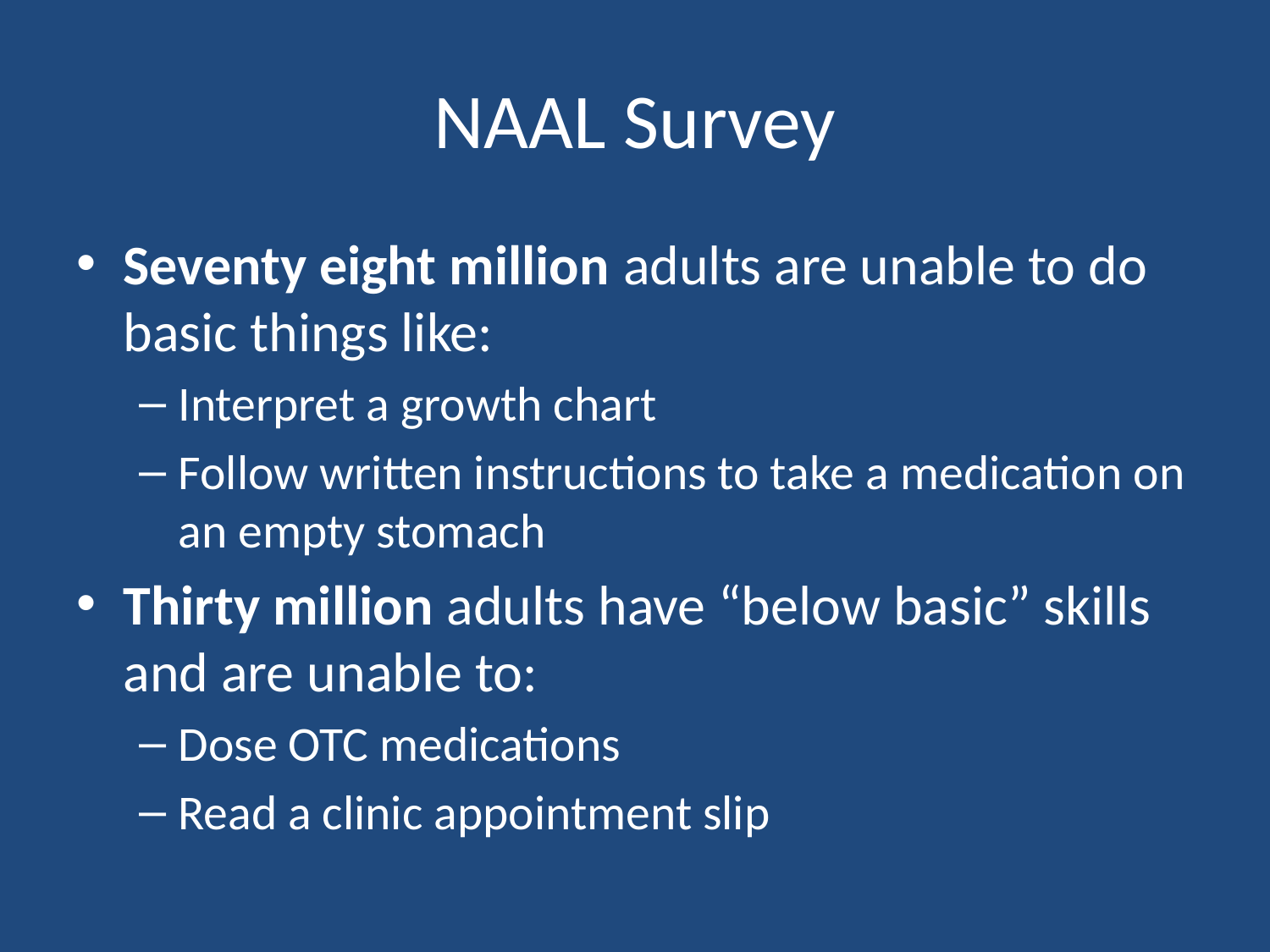

# NAAL Survey
Seventy eight million adults are unable to do basic things like:
Interpret a growth chart
Follow written instructions to take a medication on an empty stomach
Thirty million adults have “below basic” skills and are unable to:
Dose OTC medications
Read a clinic appointment slip

## Slide 13
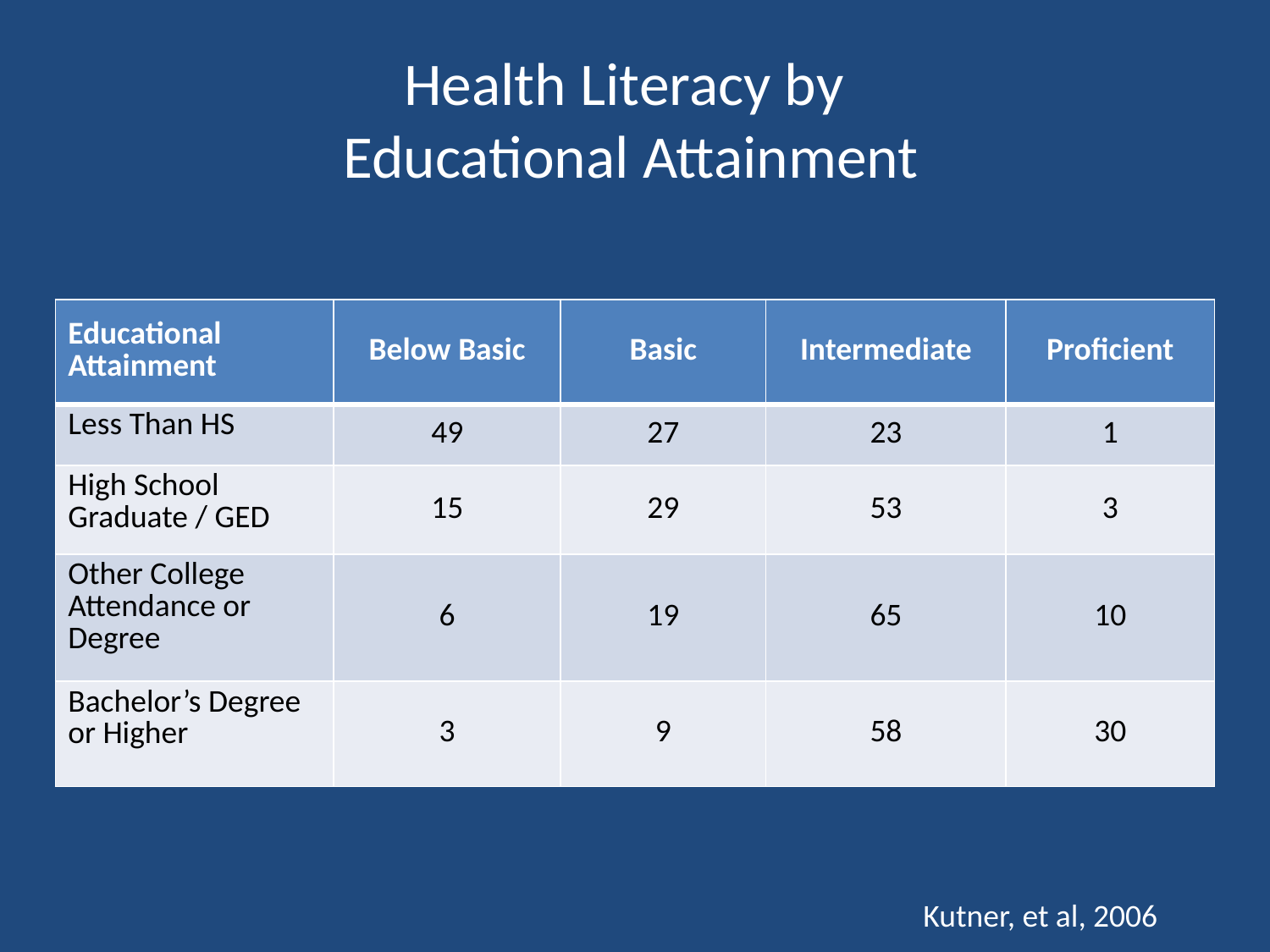

# Health Literacy by Educational Attainment
| Educational Attainment | Below Basic | Basic | Intermediate | Proficient |
| --- | --- | --- | --- | --- |
| Less Than HS | 49 | 27 | 23 | 1 |
| High School Graduate / GED | 15 | 29 | 53 | 3 |
| Other College Attendance or Degree | 6 | 19 | 65 | 10 |
| Bachelor’s Degree or Higher | 3 | 9 | 58 | 30 |
Kutner, et al, 2006

## Slide 14
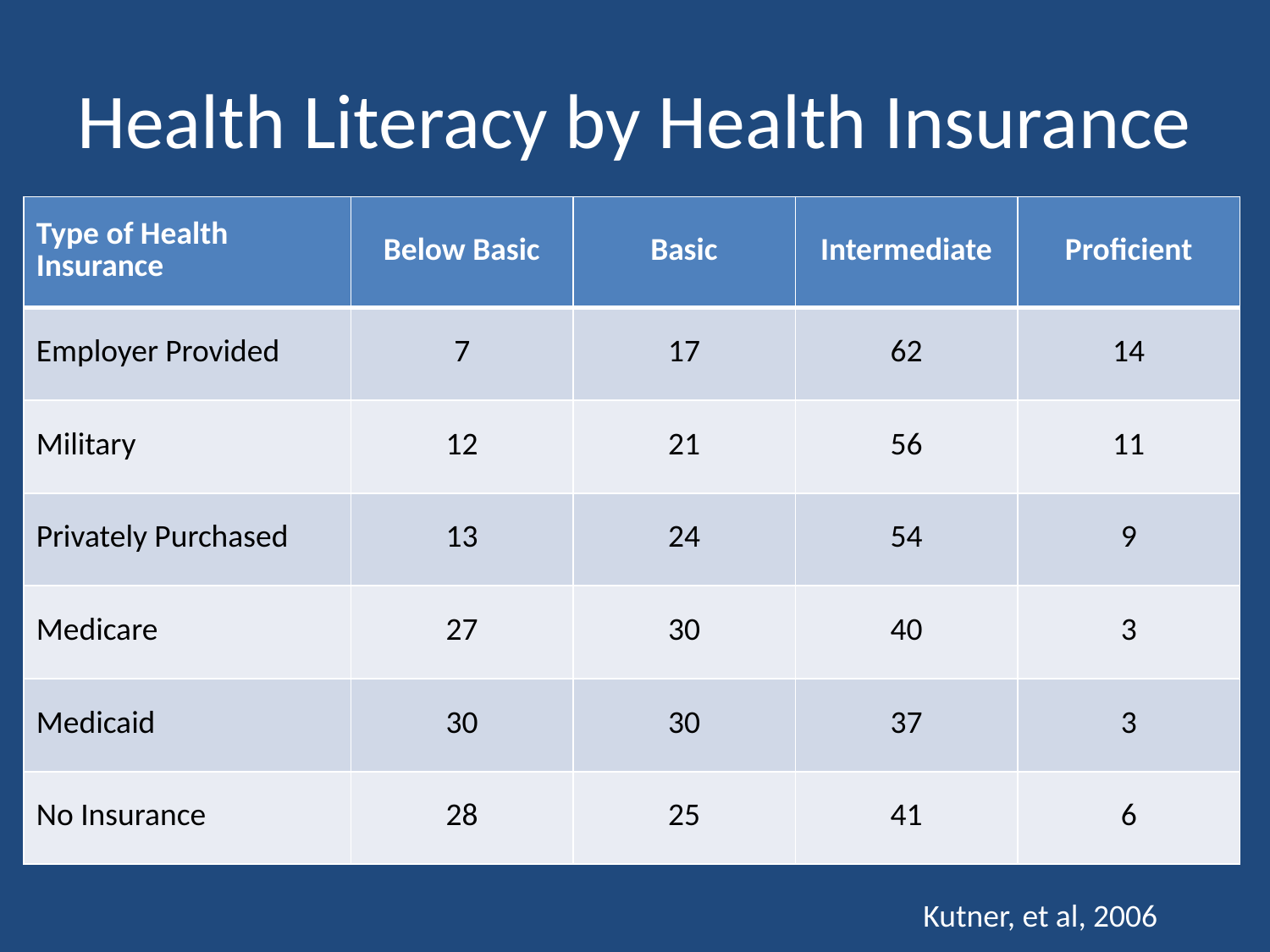

# Health Literacy by Health Insurance
| Type of Health Insurance | Below Basic | Basic | Intermediate | Proficient |
| --- | --- | --- | --- | --- |
| Employer Provided | 7 | 17 | 62 | 14 |
| Military | 12 | 21 | 56 | 11 |
| Privately Purchased | 13 | 24 | 54 | 9 |
| Medicare | 27 | 30 | 40 | 3 |
| Medicaid | 30 | 30 | 37 | 3 |
| No Insurance | 28 | 25 | 41 | 6 |
Kutner, et al, 2006

## Slide 15
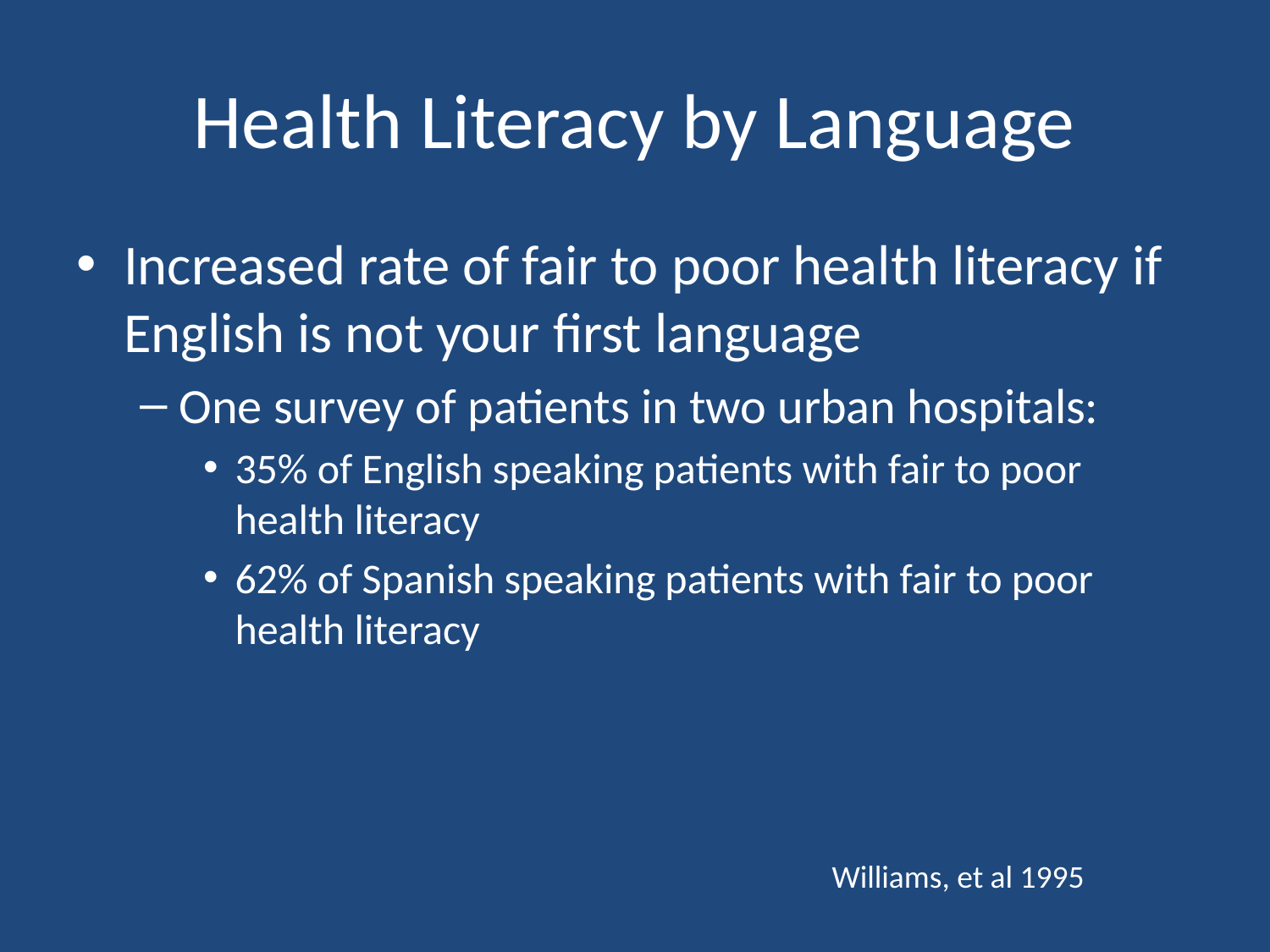

# Health Literacy by Language
Increased rate of fair to poor health literacy if English is not your first language
One survey of patients in two urban hospitals:
35% of English speaking patients with fair to poor health literacy
62% of Spanish speaking patients with fair to poor health literacy
Williams, et al 1995

## Slide 16
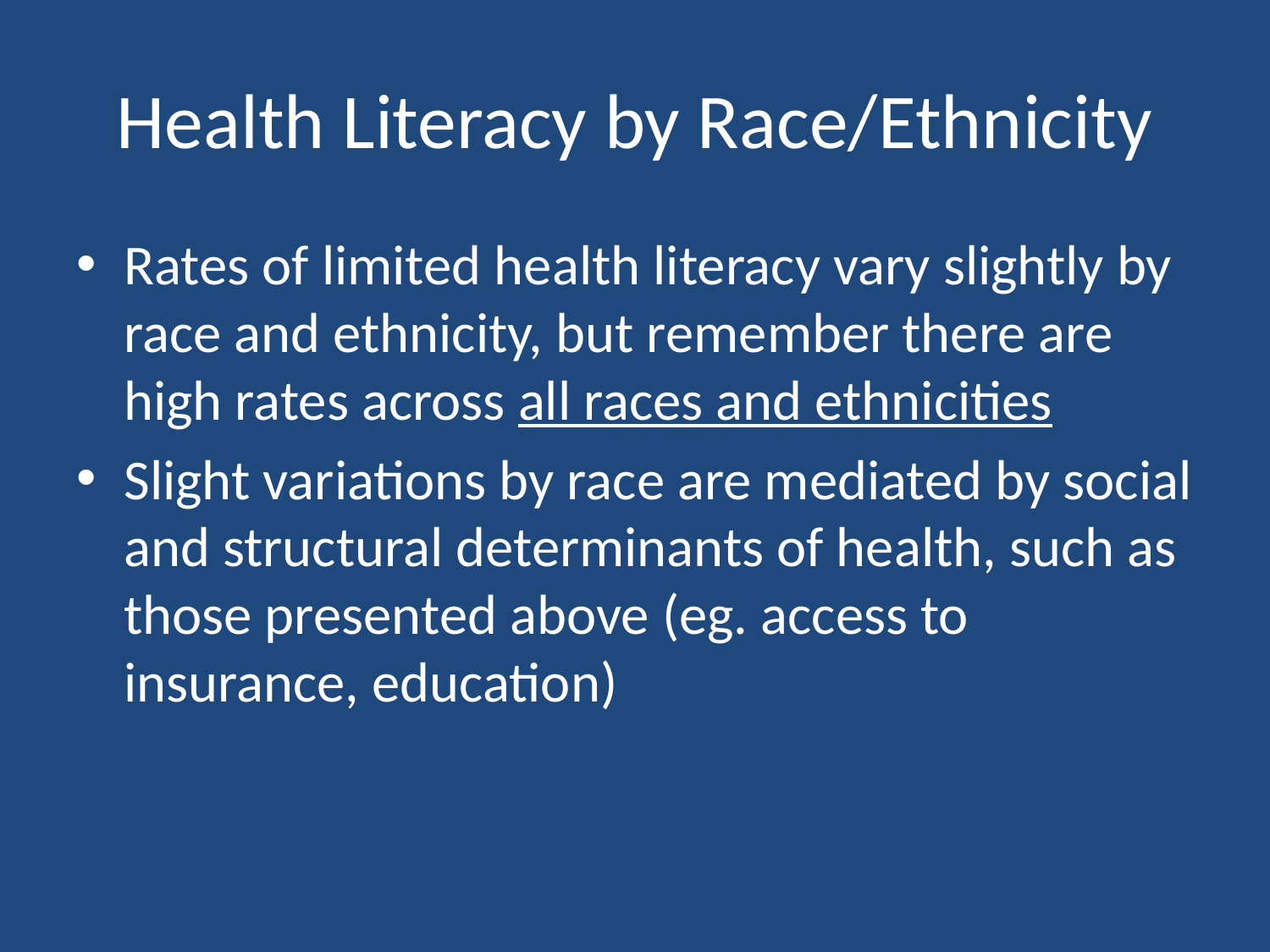

# Health Literacy by Race/Ethnicity
Rates of limited health literacy vary slightly by race and ethnicity, but remember there are high rates across all races and ethnicities
Slight variations by race are mediated by social and structural determinants of health, such as those presented above (eg. access to insurance, education)

## Slide 17
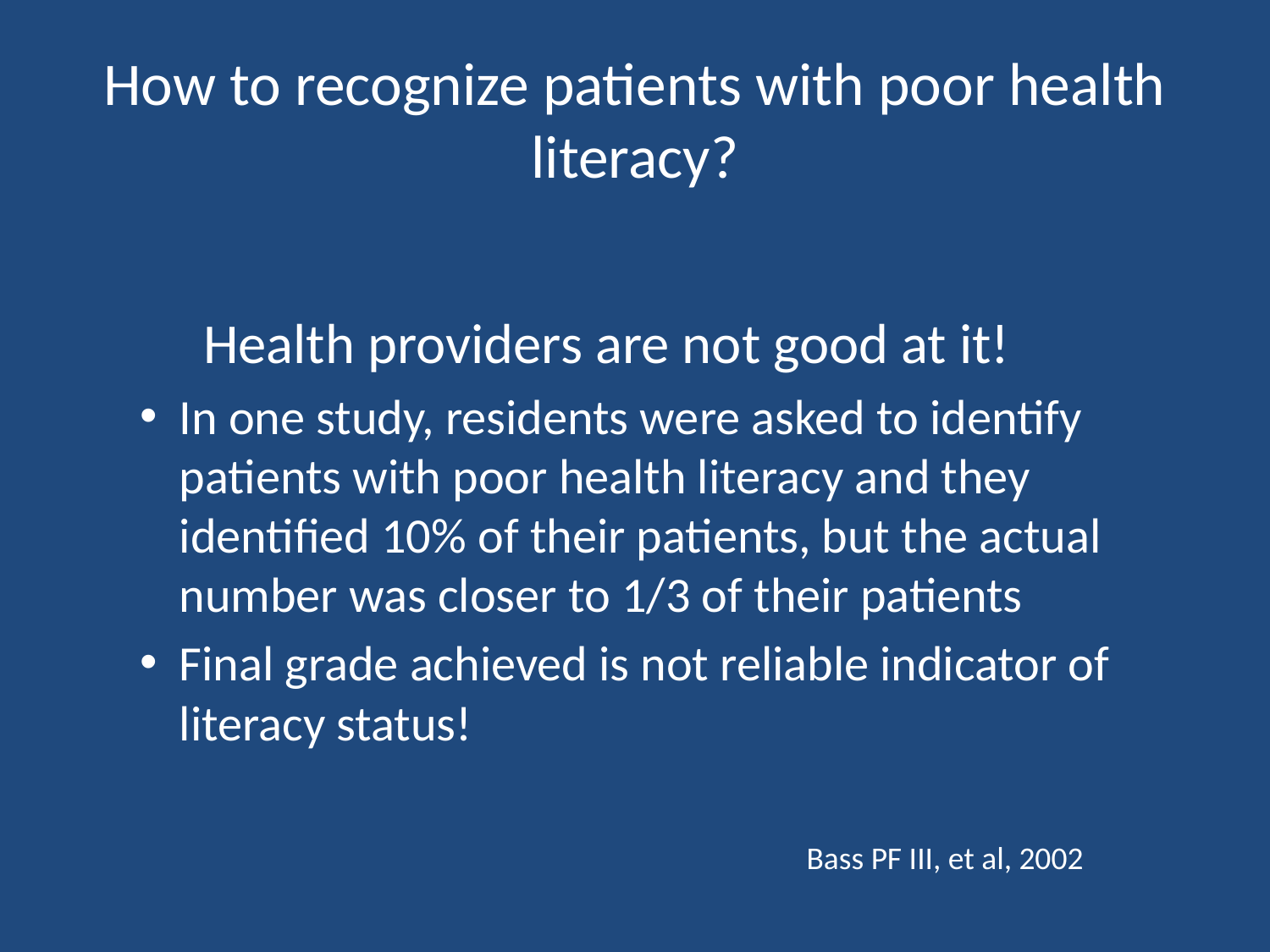

# How to recognize patients with poor health literacy?
	Health providers are not good at it!
In one study, residents were asked to identify patients with poor health literacy and they identified 10% of their patients, but the actual number was closer to 1/3 of their patients
Final grade achieved is not reliable indicator of literacy status!
Bass PF III, et al, 2002

## Slide 18
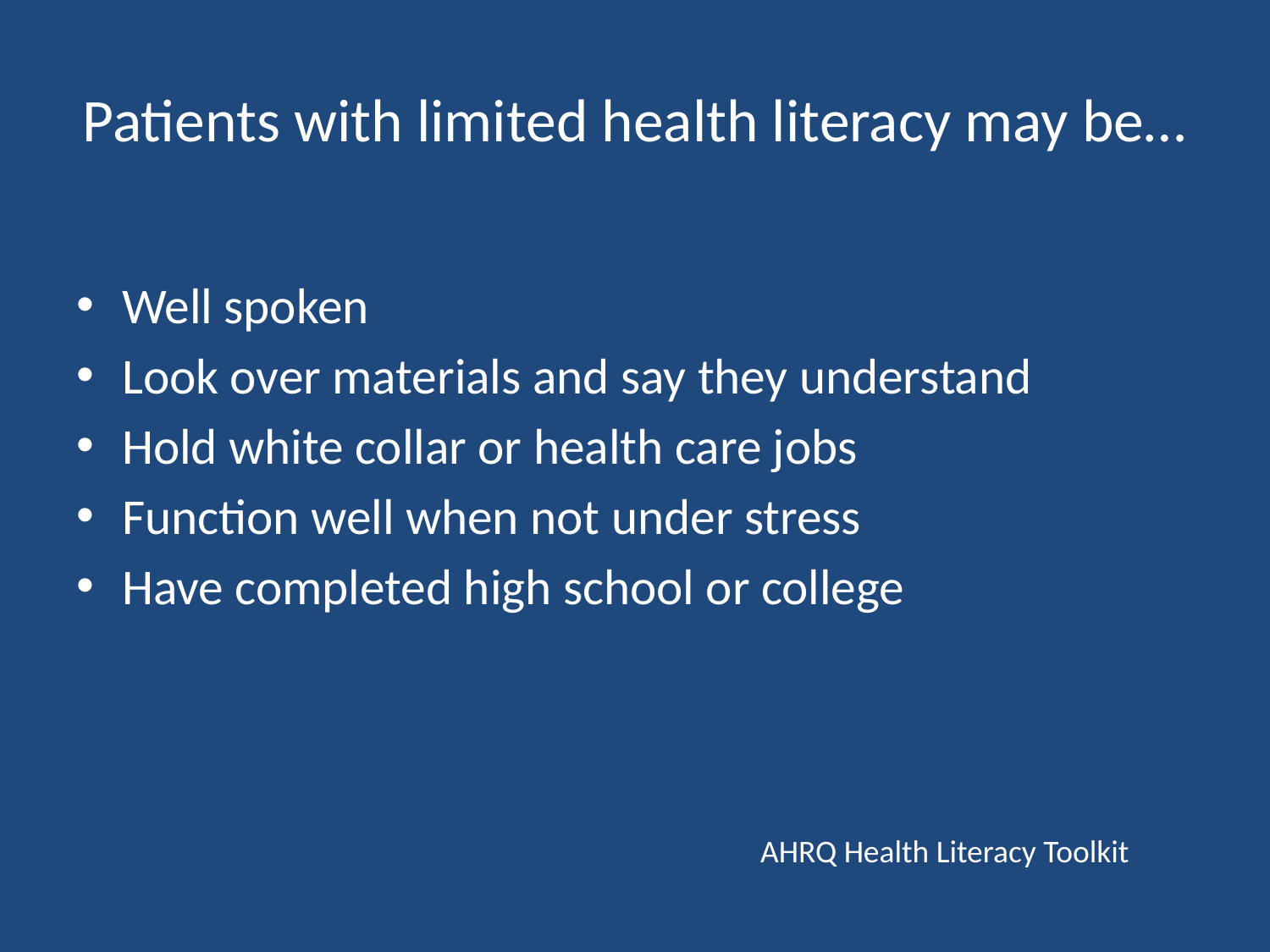

# Patients with limited health literacy may be…
Well spoken
Look over materials and say they understand
Hold white collar or health care jobs
Function well when not under stress
Have completed high school or college
AHRQ Health Literacy Toolkit

## Slide 19
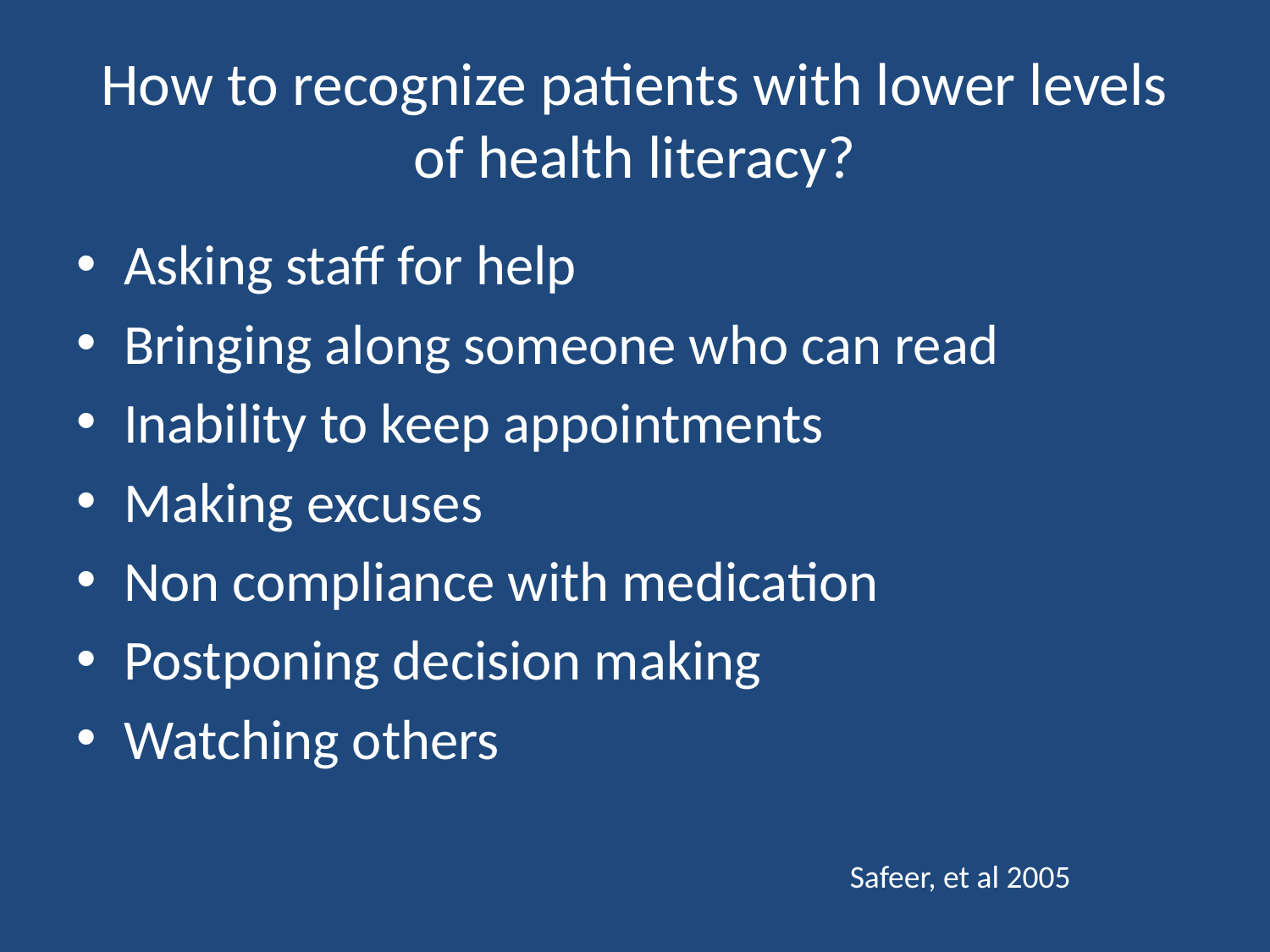

# How to recognize patients with lower levels of health literacy?
Asking staff for help
Bringing along someone who can read
Inability to keep appointments
Making excuses
Non compliance with medication
Postponing decision making
Watching others
Safeer, et al 2005

## Slide 20
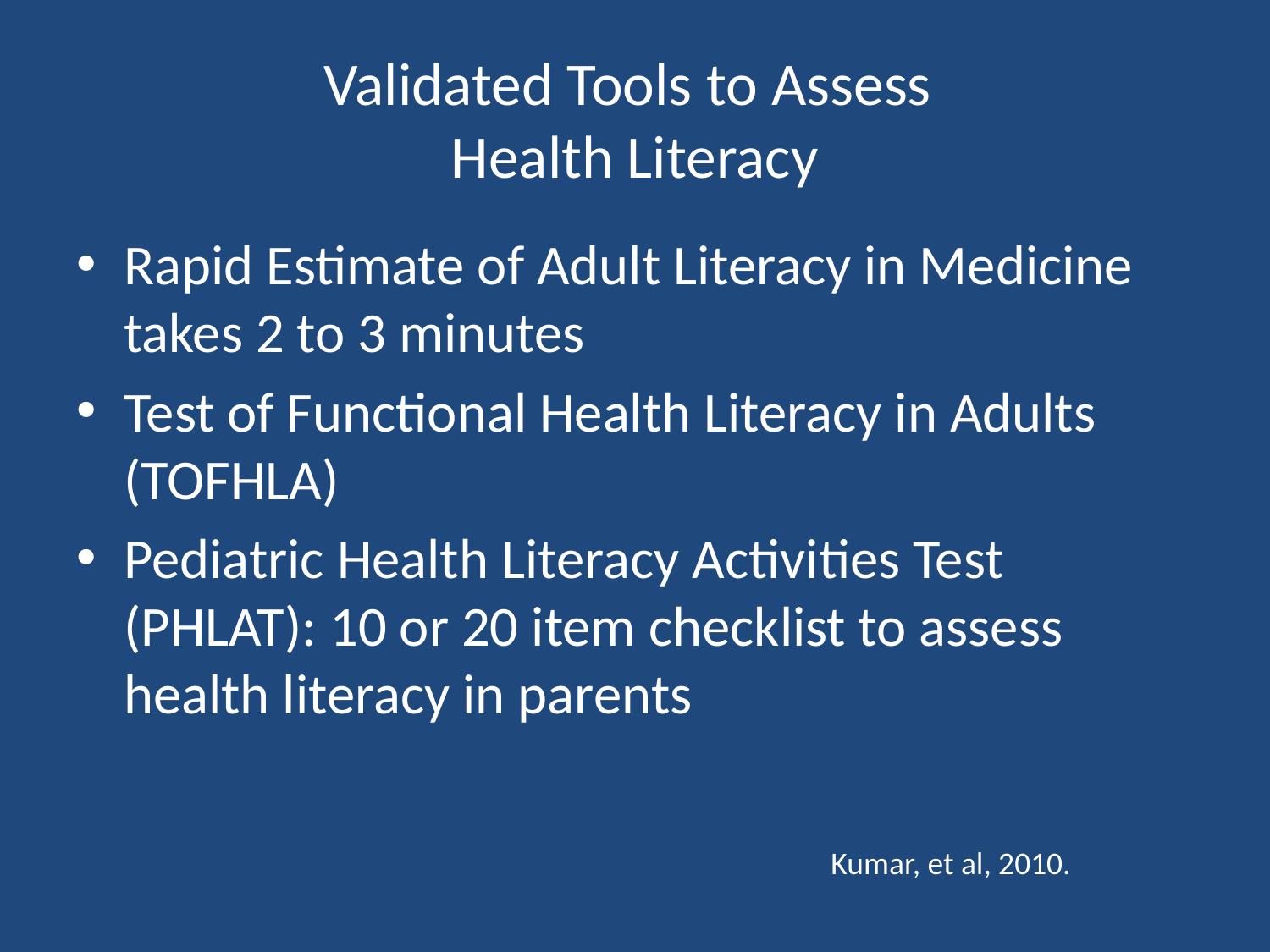

# Validated Tools to Assess Health Literacy
Rapid Estimate of Adult Literacy in Medicine takes 2 to 3 minutes
Test of Functional Health Literacy in Adults (TOFHLA)
Pediatric Health Literacy Activities Test (PHLAT): 10 or 20 item checklist to assess health literacy in parents
Kumar, et al, 2010.

## Slide 21
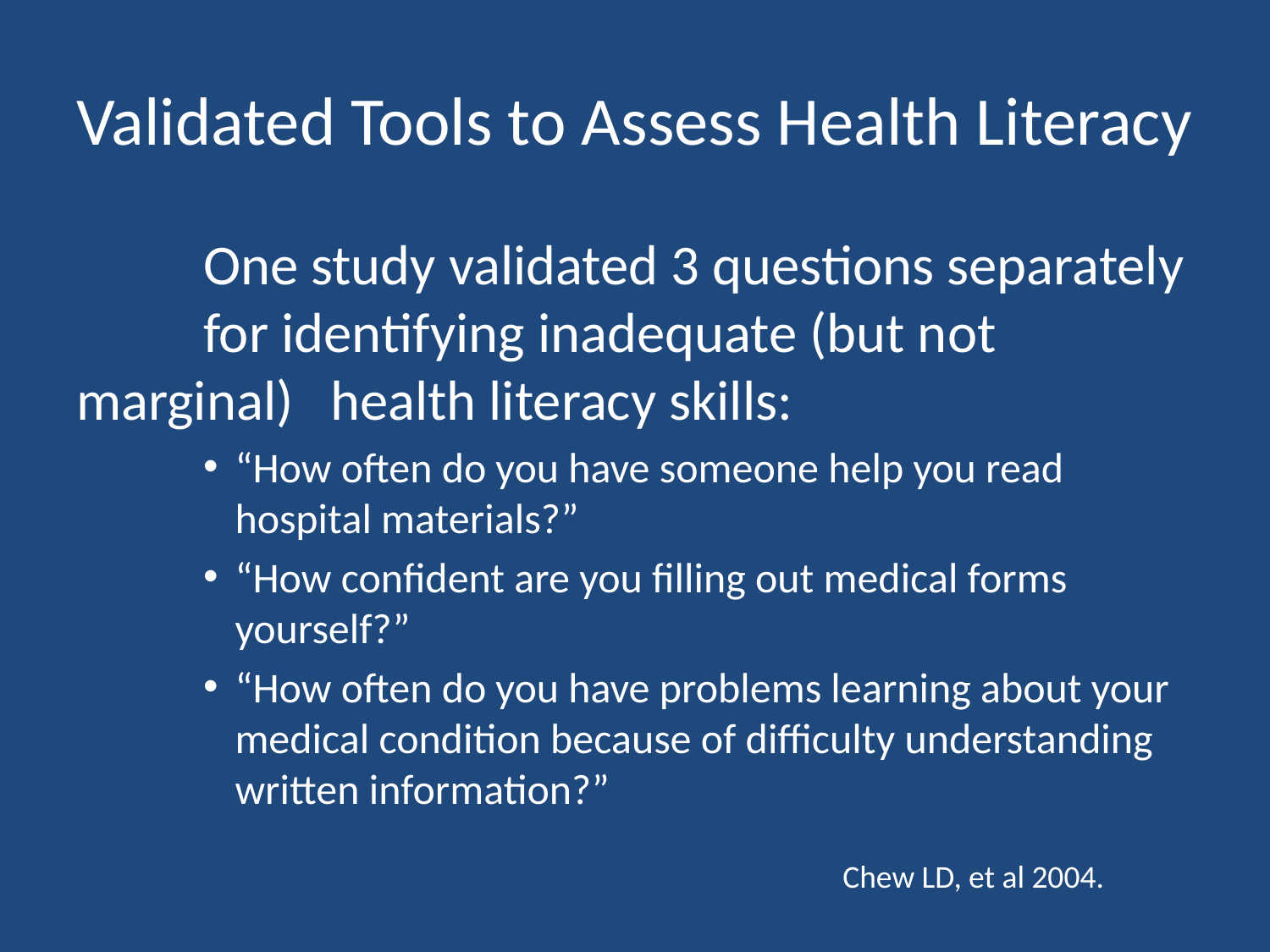

# Validated Tools to Assess Health Literacy
	One study validated 3 questions separately 	for identifying inadequate (but not marginal) 	health literacy skills:
“How often do you have someone help you read hospital materials?”
“How confident are you filling out medical forms yourself?”
“How often do you have problems learning about your medical condition because of difficulty understanding written information?”
Chew LD, et al 2004.

## Slide 22
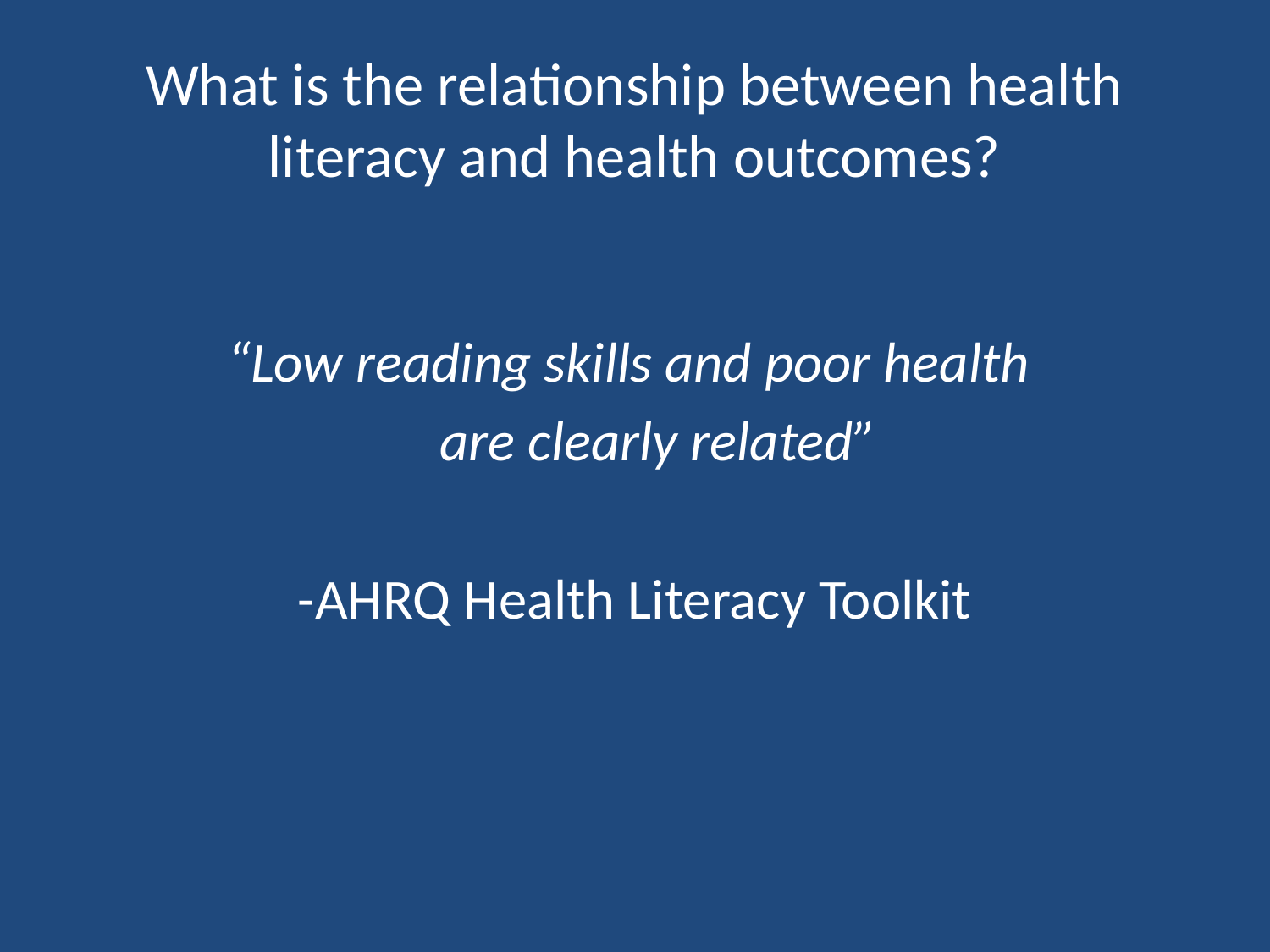

# What is the relationship between health literacy and health outcomes?
“Low reading skills and poor health
	are clearly related”
-AHRQ Health Literacy Toolkit

## Slide 23
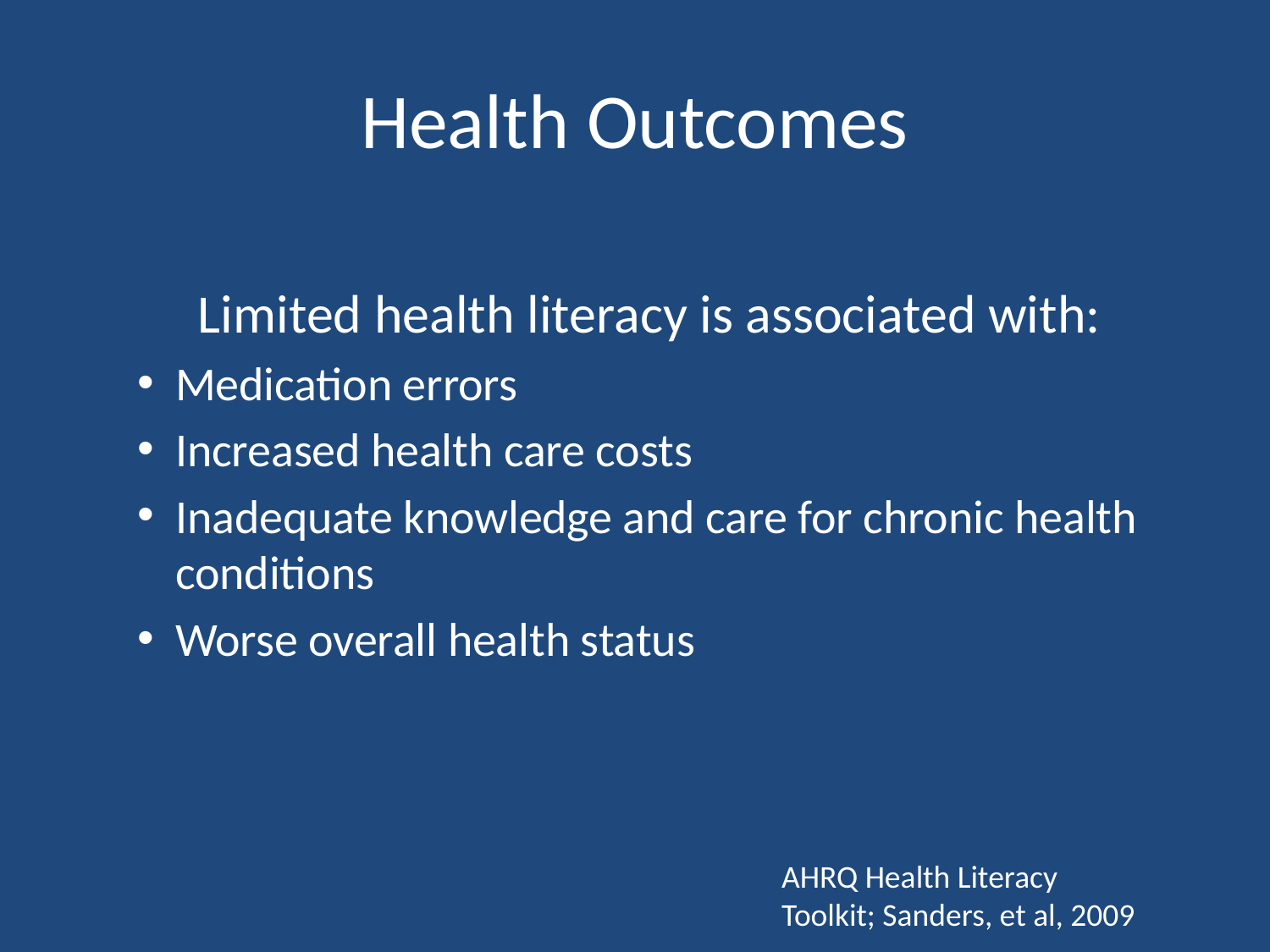

# Health Outcomes
	Limited health literacy is associated with:
Medication errors
Increased health care costs
Inadequate knowledge and care for chronic health conditions
Worse overall health status
AHRQ Health Literacy Toolkit; Sanders, et al, 2009

## Slide 24
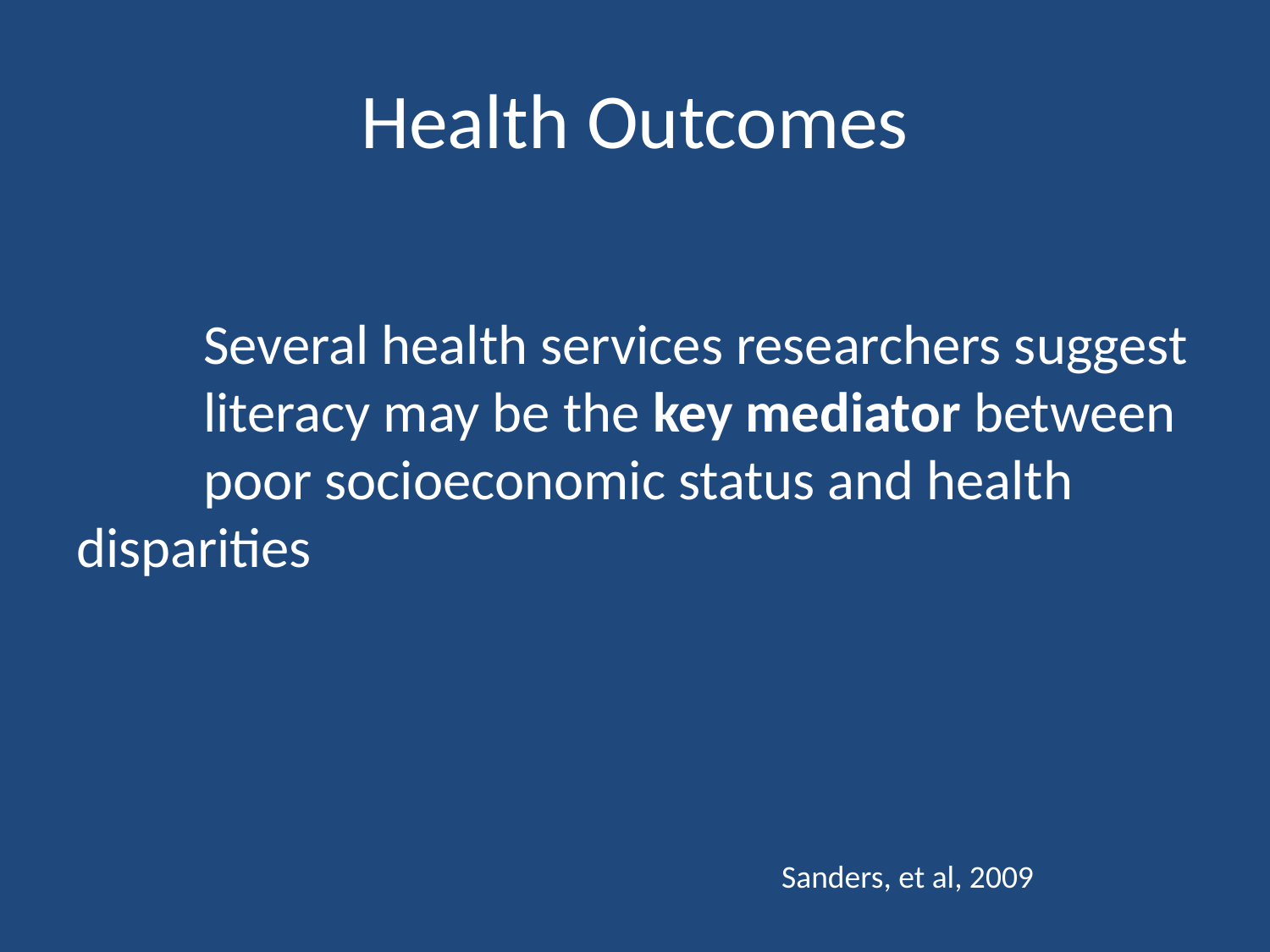

# Health Outcomes
	Several health services researchers suggest 	literacy may be the key mediator between 	poor socioeconomic status and health 	disparities
Sanders, et al, 2009

## Slide 25
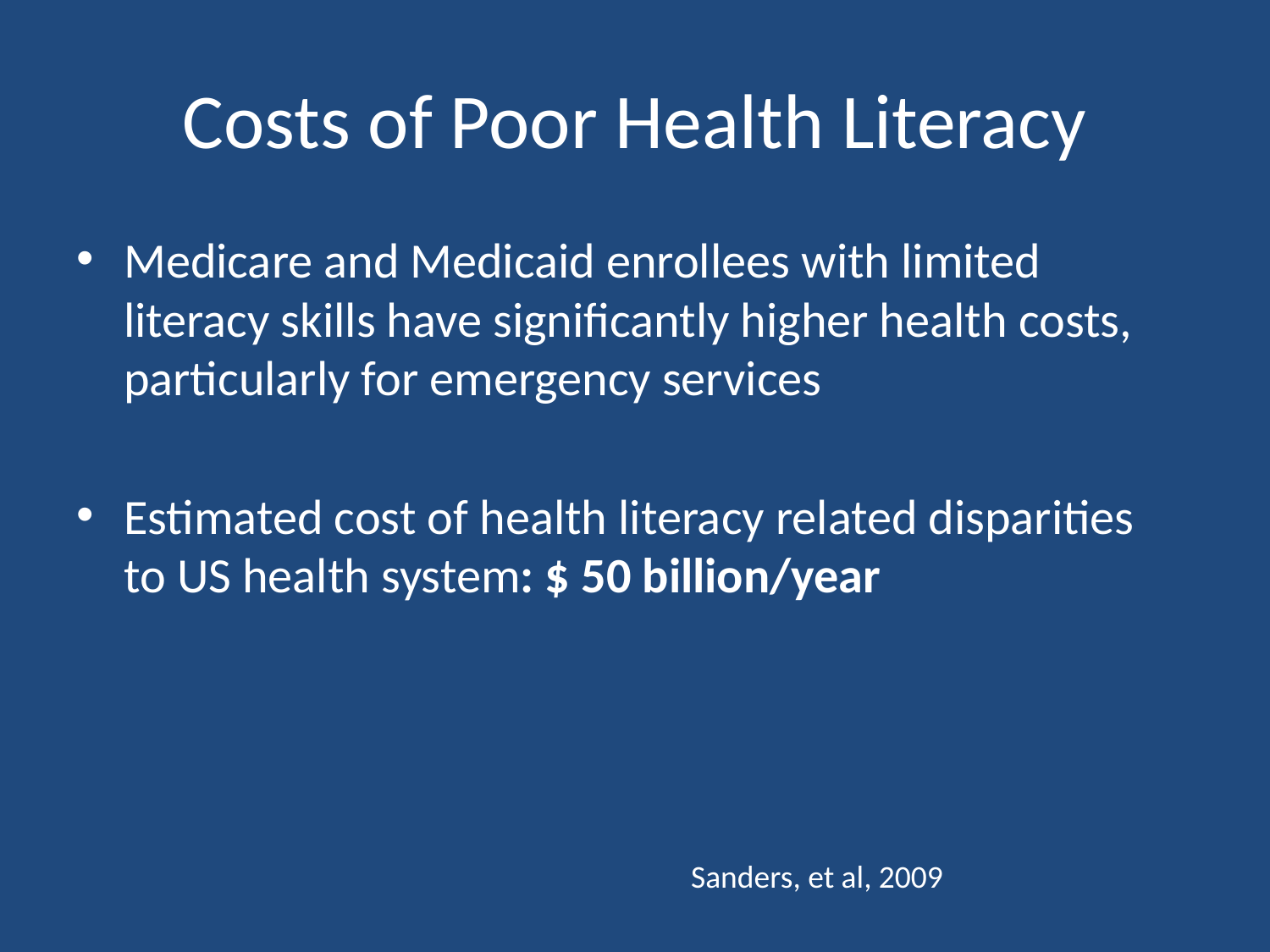

# Costs of Poor Health Literacy
Medicare and Medicaid enrollees with limited literacy skills have significantly higher health costs, particularly for emergency services
Estimated cost of health literacy related disparities to US health system: $ 50 billion/year
Sanders, et al, 2009

## Slide 26
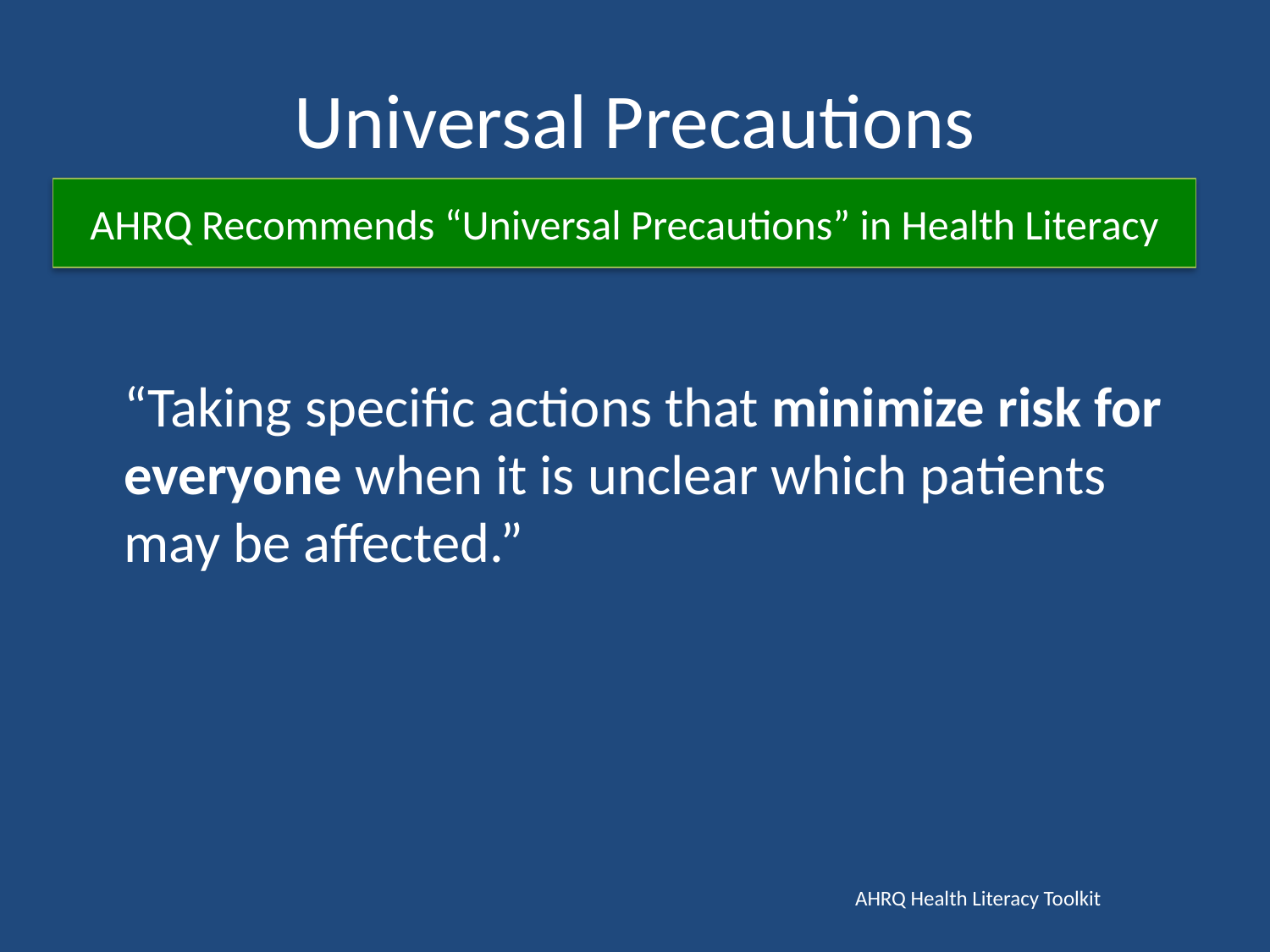

# Universal Precautions
AHRQ Recommends “Universal Precautions” in Health Literacy
	“Taking specific actions that minimize risk for everyone when it is unclear which patients may be affected.”
AHRQ Health Literacy Toolkit

## Slide 27
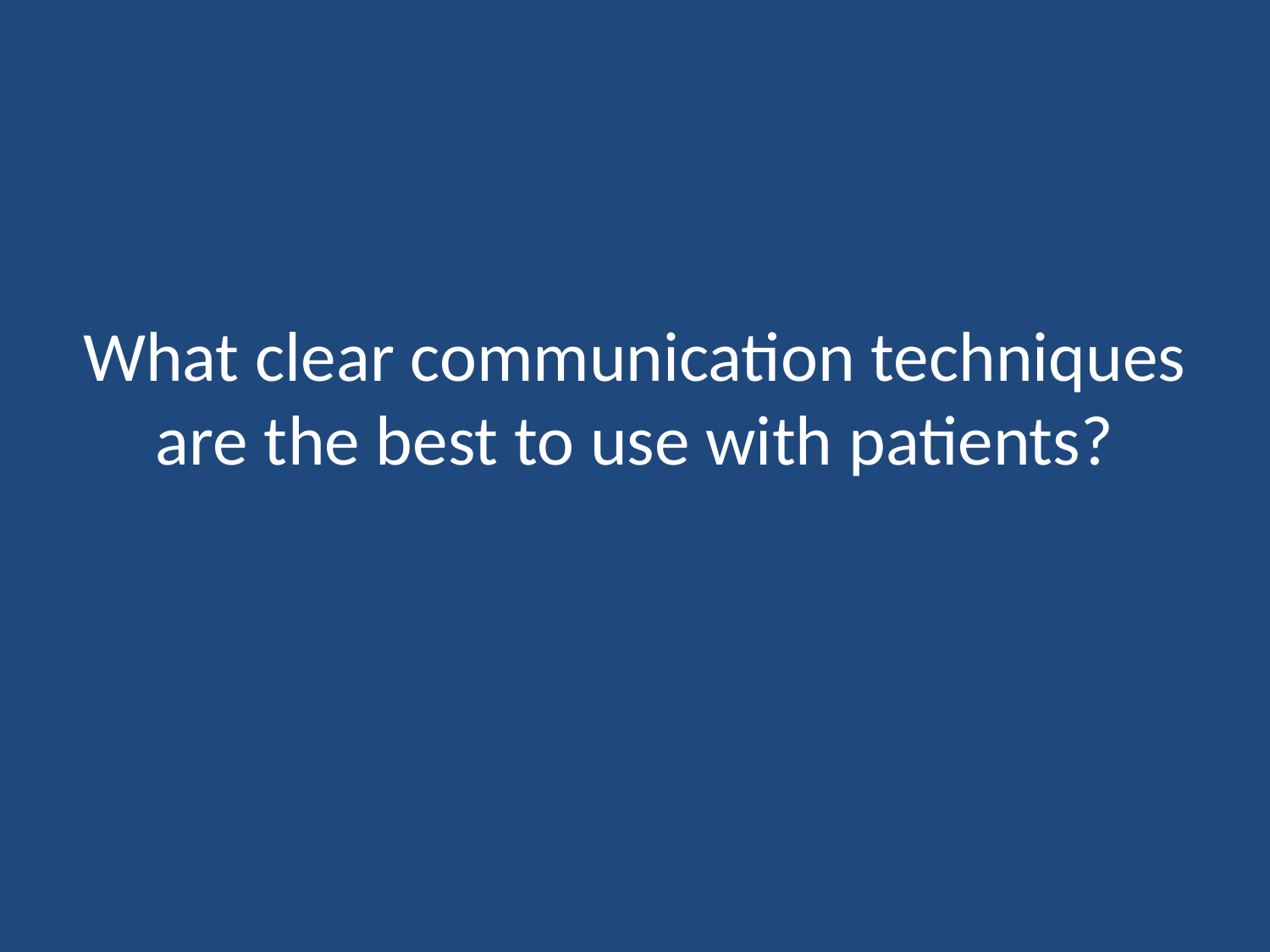

What clear communication techniques are the best to use with patients?

## Slide 28
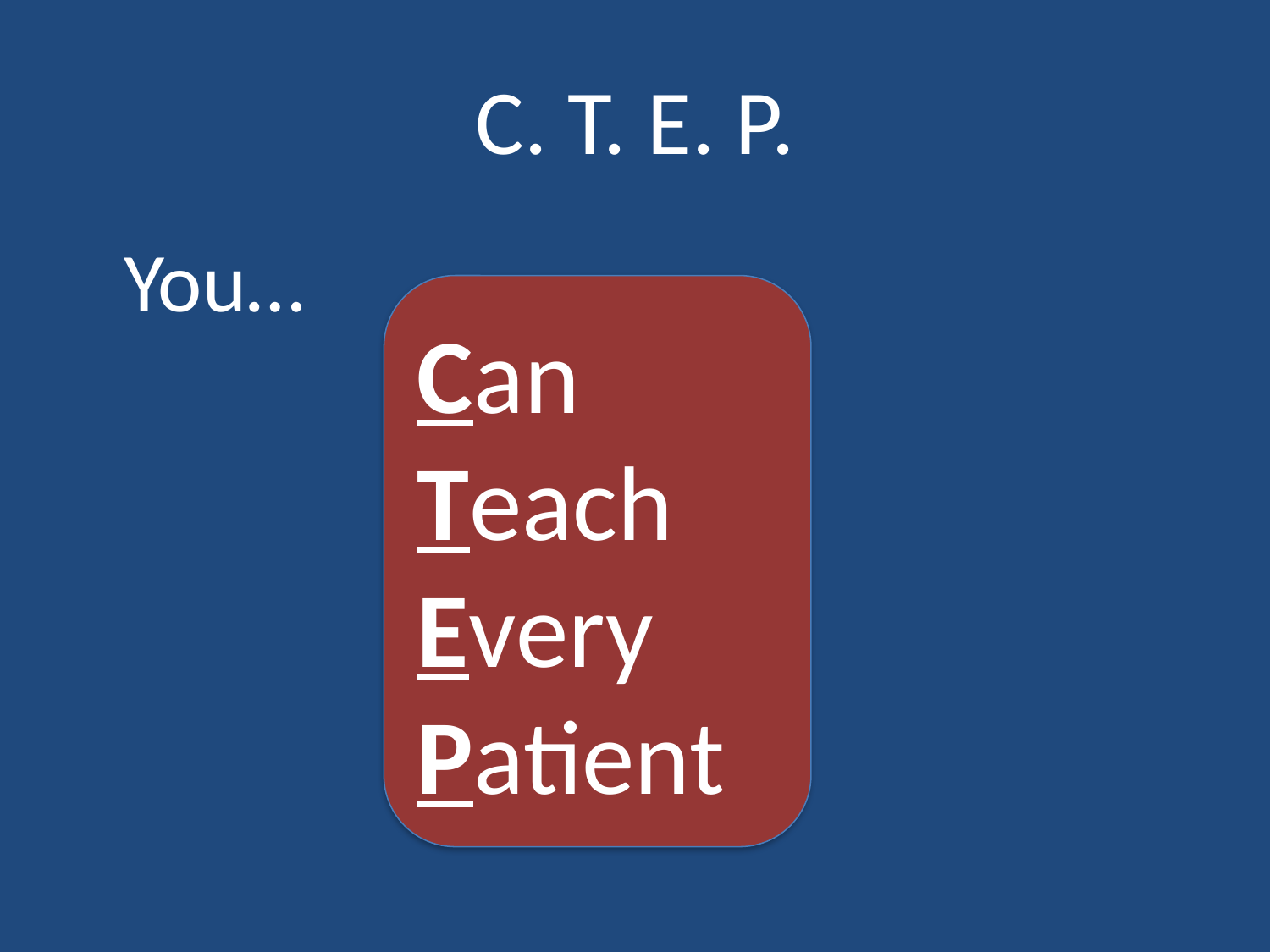

# C. T. E. P.
	You…
Can
Teach
Every
Patient

## Slide 29
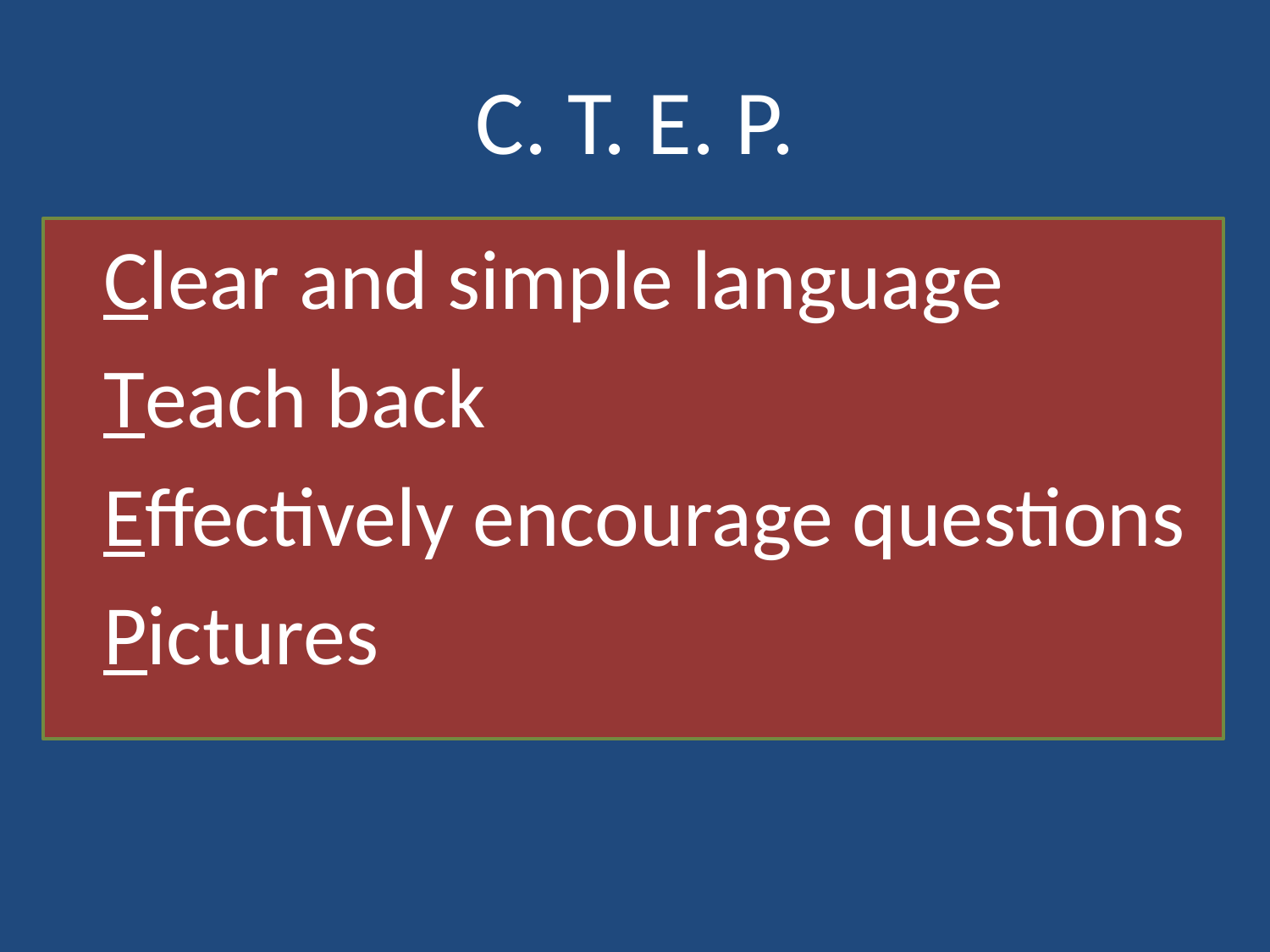

# C. T. E. P.
	Clear and simple language
	Teach back
	Effectively encourage questions
	Pictures

## Slide 30
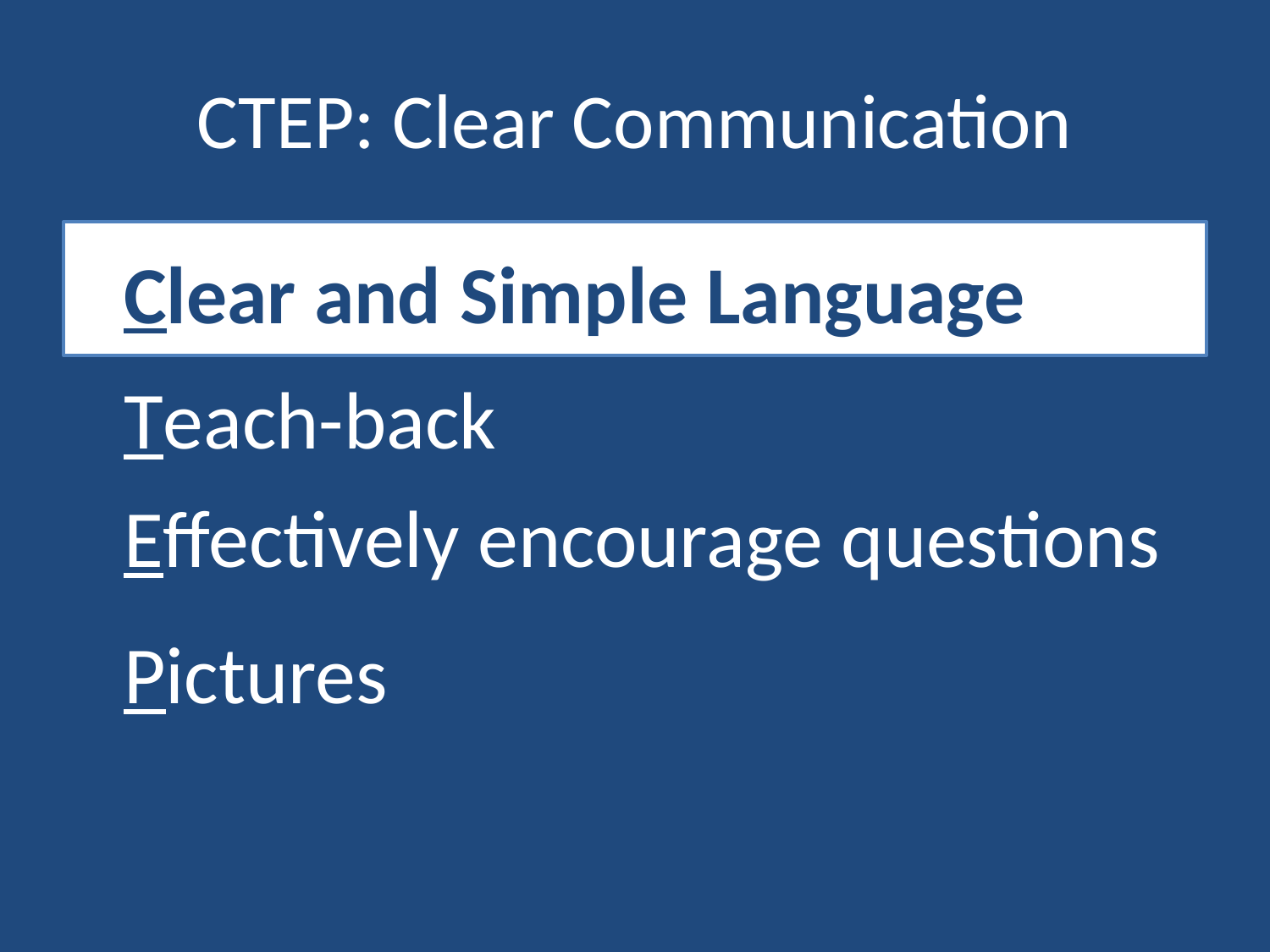

# CTEP: Clear Communication
	Clear and Simple Language
	Teach-back
	Effectively encourage questions
	Pictures

## Slide 31
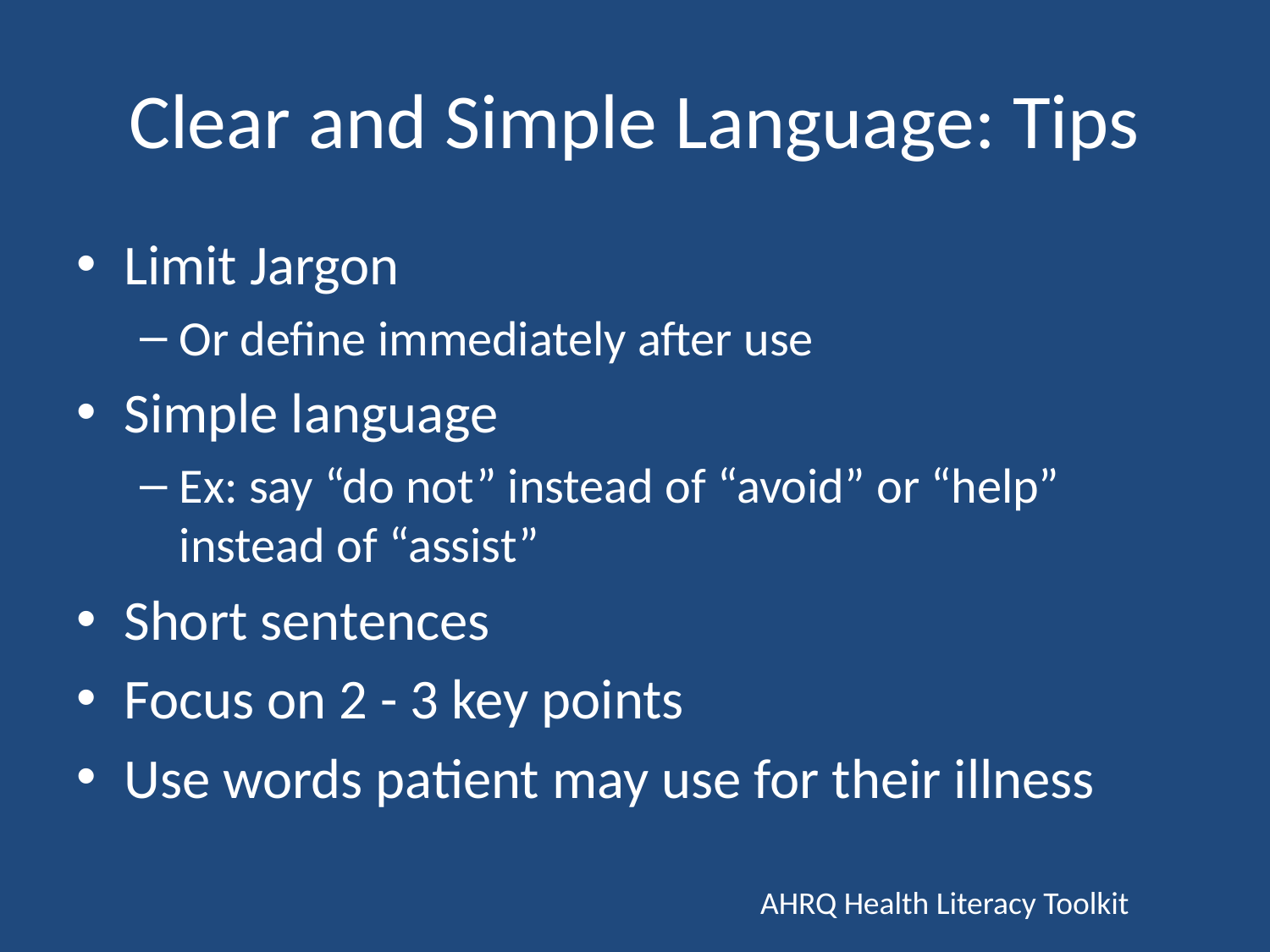

# Clear and Simple Language: Tips
Limit Jargon
Or define immediately after use
Simple language
Ex: say “do not” instead of “avoid” or “help” instead of “assist”
Short sentences
Focus on 2 - 3 key points
Use words patient may use for their illness
AHRQ Health Literacy Toolkit

## Slide 32
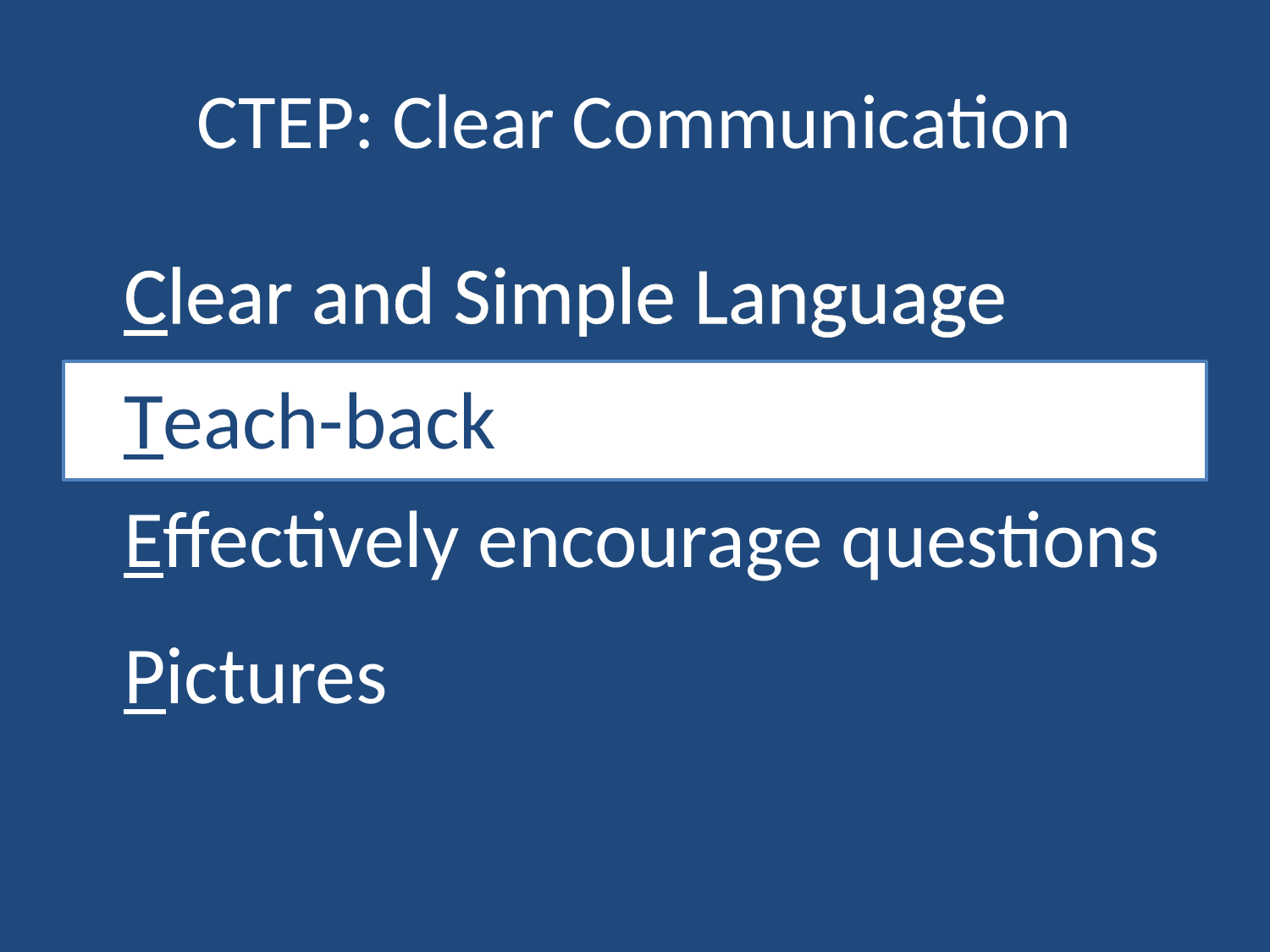

# CTEP: Clear Communication
	Clear and Simple Language
	Teach-back
	Effectively encourage questions
	Pictures

## Slide 33
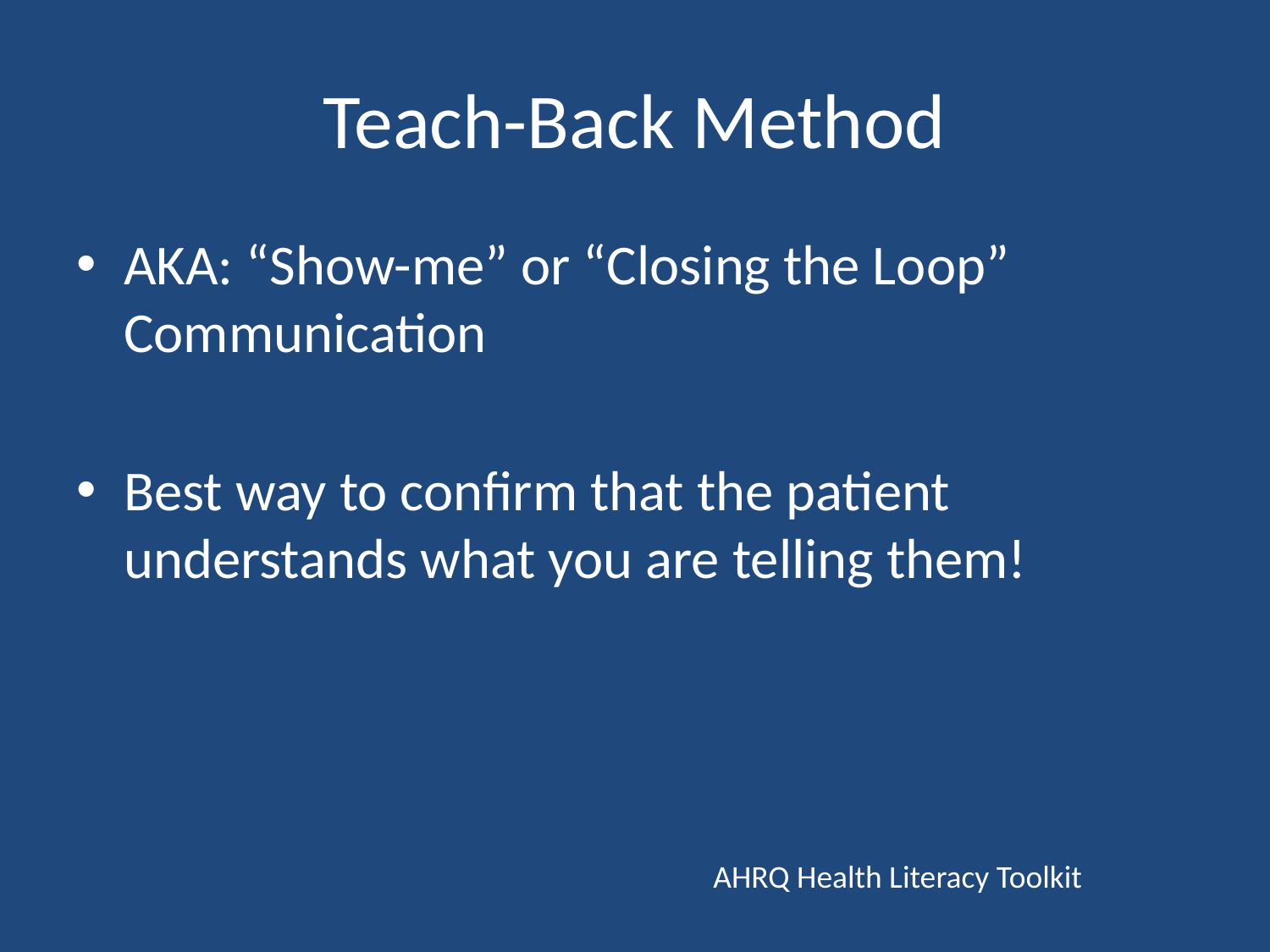

# Teach-Back Method
AKA: “Show-me” or “Closing the Loop” Communication
Best way to confirm that the patient understands what you are telling them!
AHRQ Health Literacy Toolkit

## Slide 34
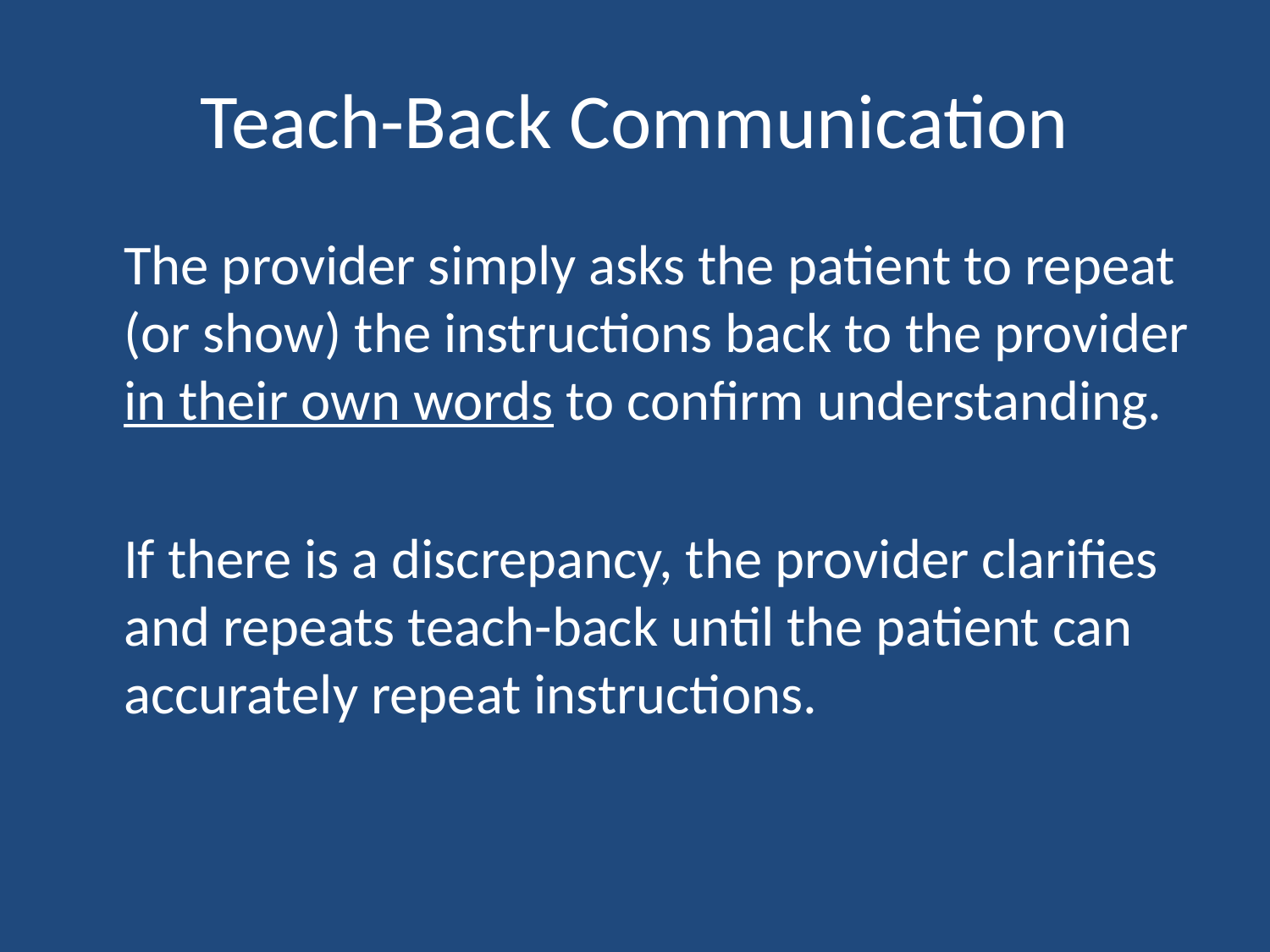

# Teach-Back Communication
	The provider simply asks the patient to repeat (or show) the instructions back to the provider in their own words to confirm understanding.
	If there is a discrepancy, the provider clarifies and repeats teach-back until the patient can accurately repeat instructions.

## Slide 35
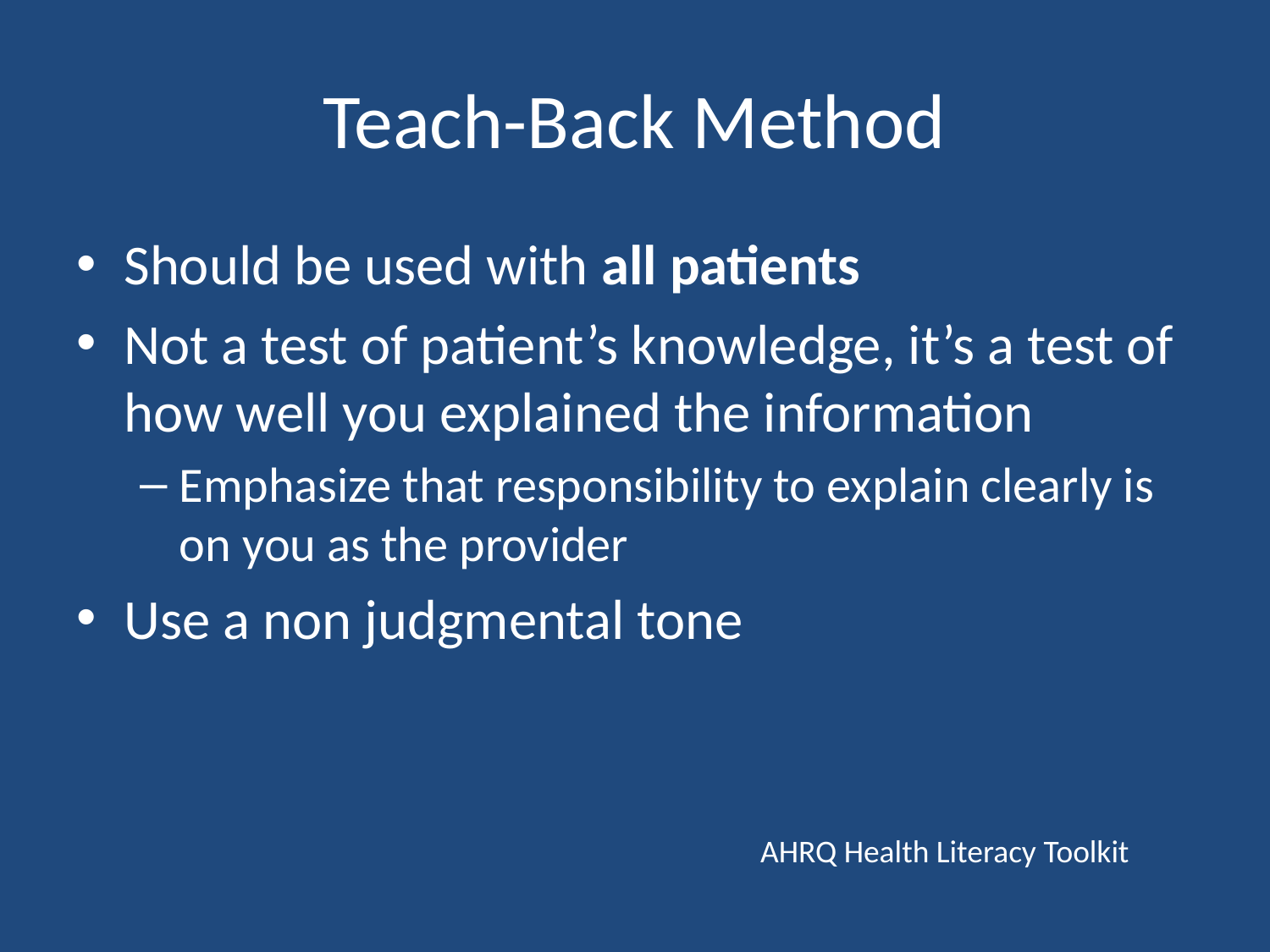

# Teach-Back Method
Should be used with all patients
Not a test of patient’s knowledge, it’s a test of how well you explained the information
Emphasize that responsibility to explain clearly is on you as the provider
Use a non judgmental tone
AHRQ Health Literacy Toolkit

## Slide 36
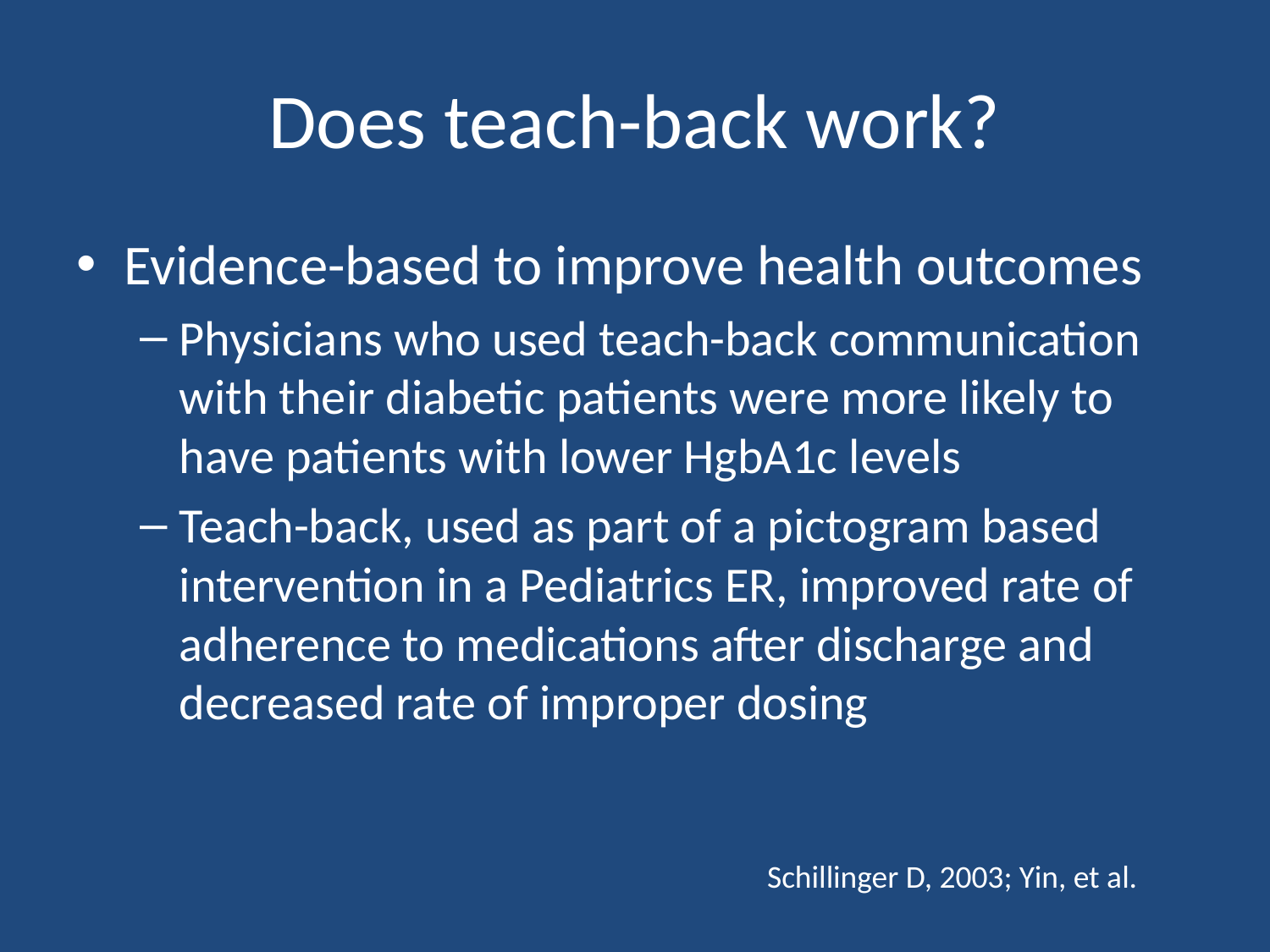

# Does teach-back work?
Evidence-based to improve health outcomes
Physicians who used teach-back communication with their diabetic patients were more likely to have patients with lower HgbA1c levels
Teach-back, used as part of a pictogram based intervention in a Pediatrics ER, improved rate of adherence to medications after discharge and decreased rate of improper dosing
Schillinger D, 2003; Yin, et al.

## Slide 37
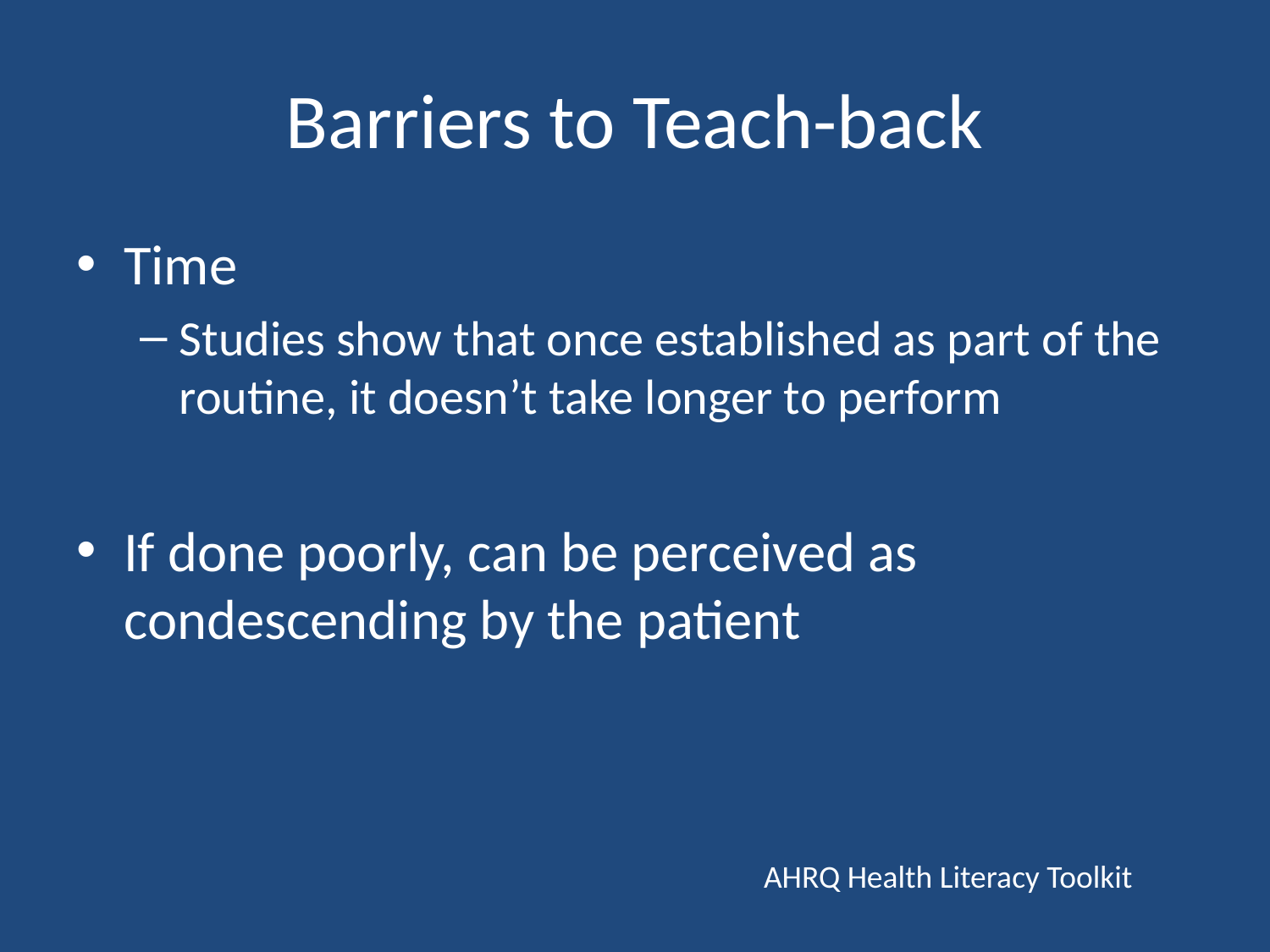

# Barriers to Teach-back
Time
Studies show that once established as part of the routine, it doesn’t take longer to perform
If done poorly, can be perceived as condescending by the patient
AHRQ Health Literacy Toolkit

## Slide 38
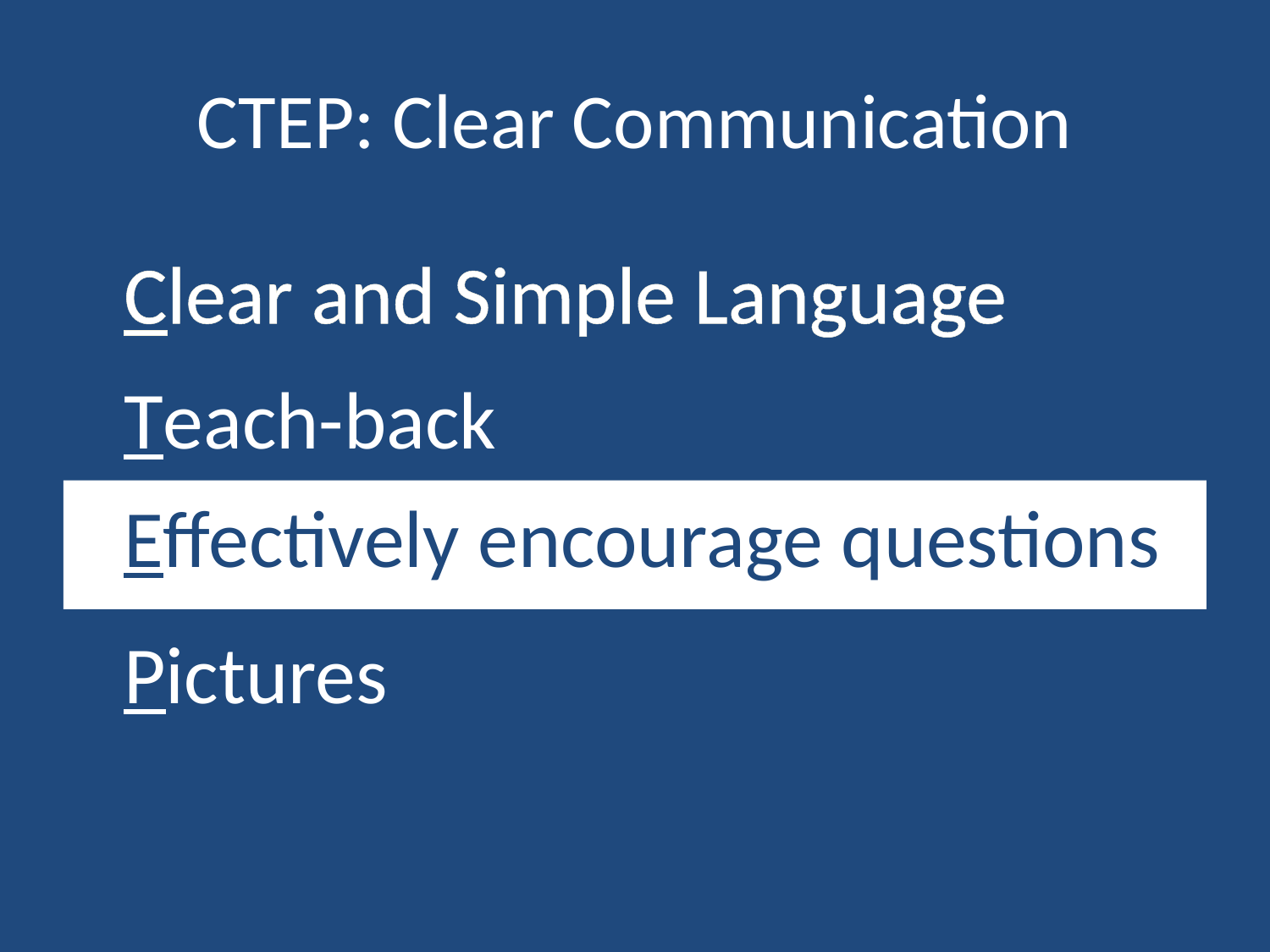

# CTEP: Clear Communication
	Clear and Simple Language
	Teach-back
	Effectively encourage questions
	Pictures

## Slide 39
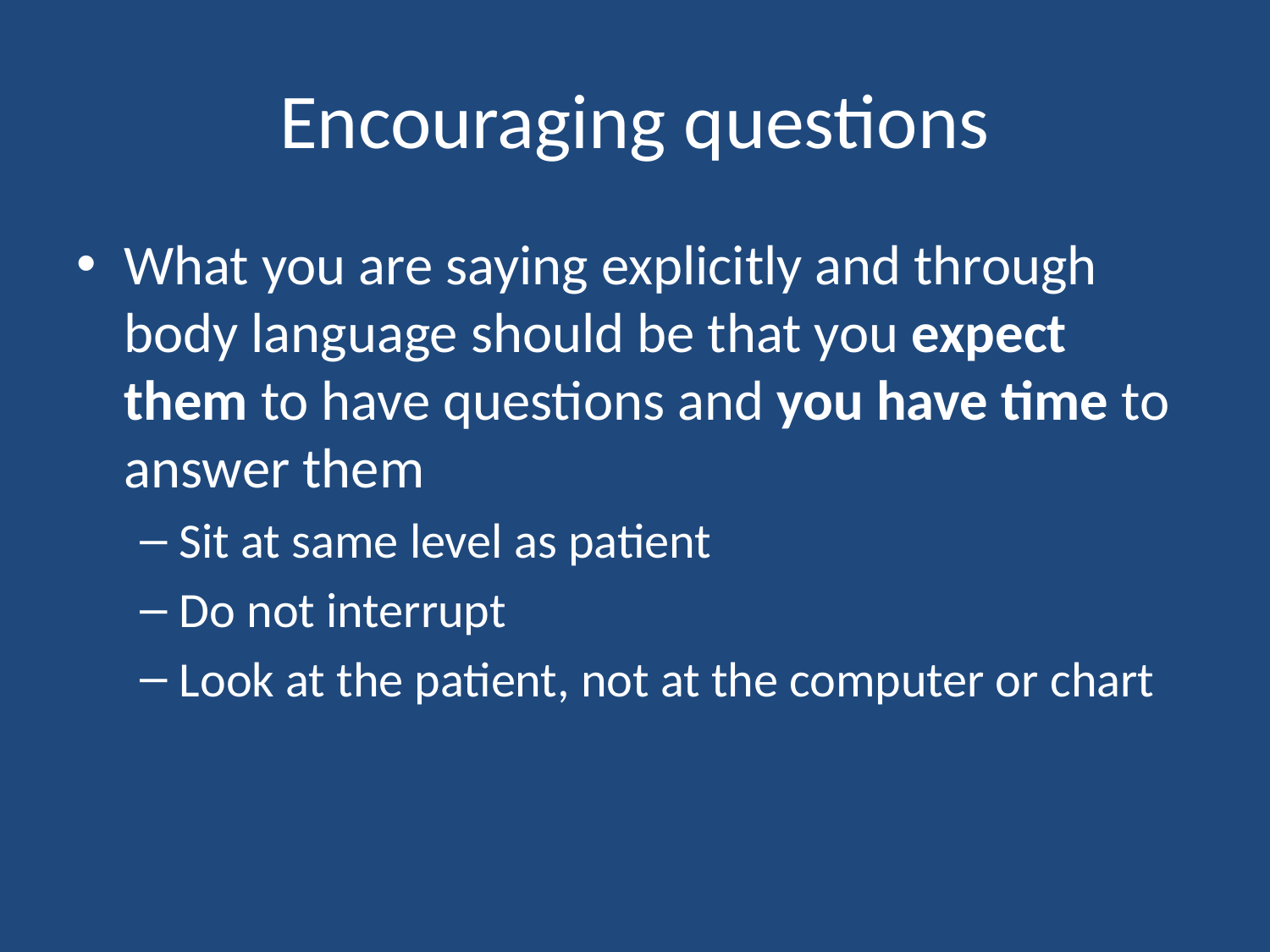

# Encouraging questions
What you are saying explicitly and through body language should be that you expect them to have questions and you have time to answer them
Sit at same level as patient
Do not interrupt
Look at the patient, not at the computer or chart

## Slide 40
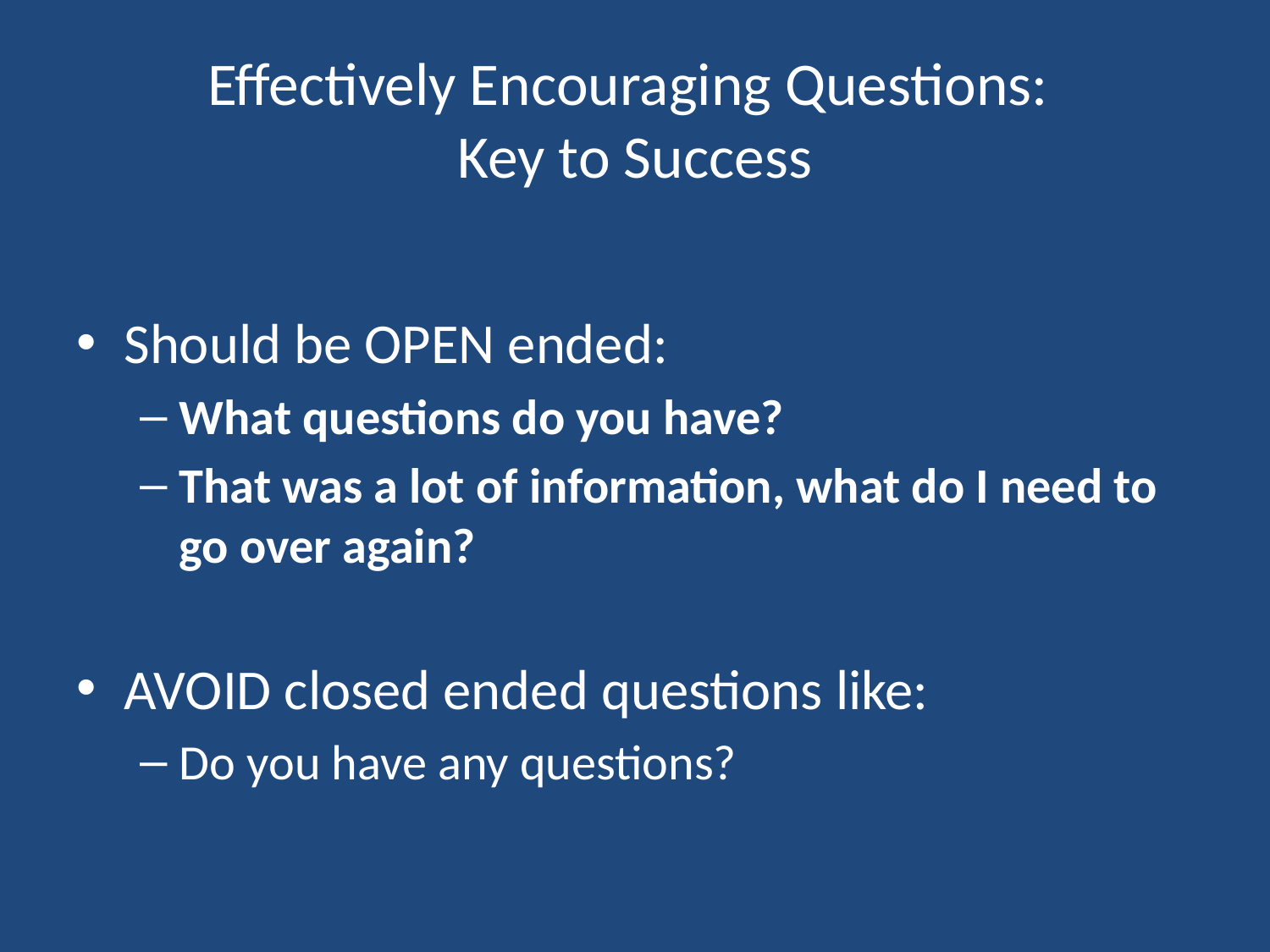

# Effectively Encouraging Questions: Key to Success
Should be OPEN ended:
What questions do you have?
That was a lot of information, what do I need to go over again?
AVOID closed ended questions like:
Do you have any questions?

## Slide 41
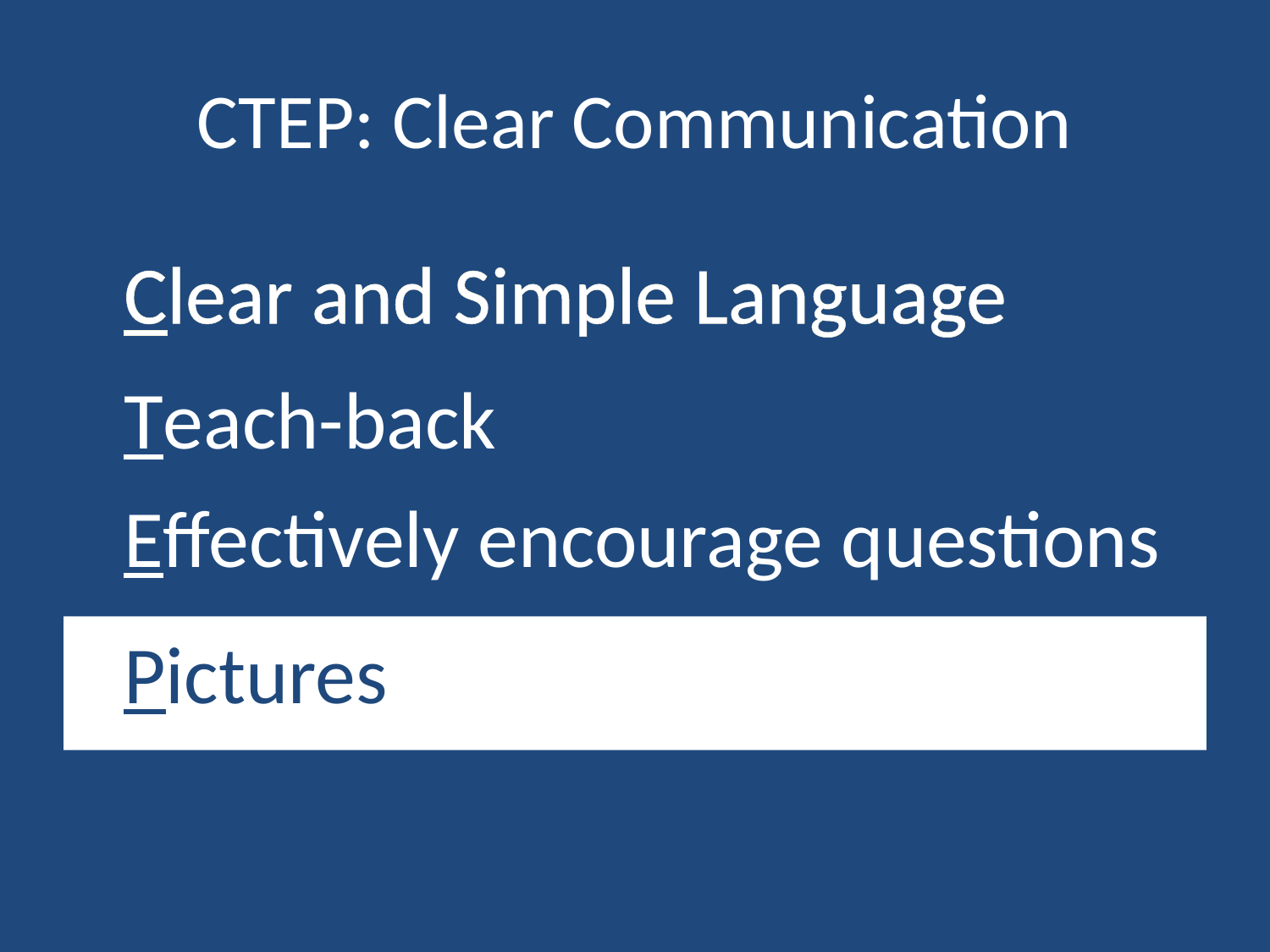

# CTEP: Clear Communication
	Clear and Simple Language
	Teach-back
	Effectively encourage questions
	Pictures

## Slide 42
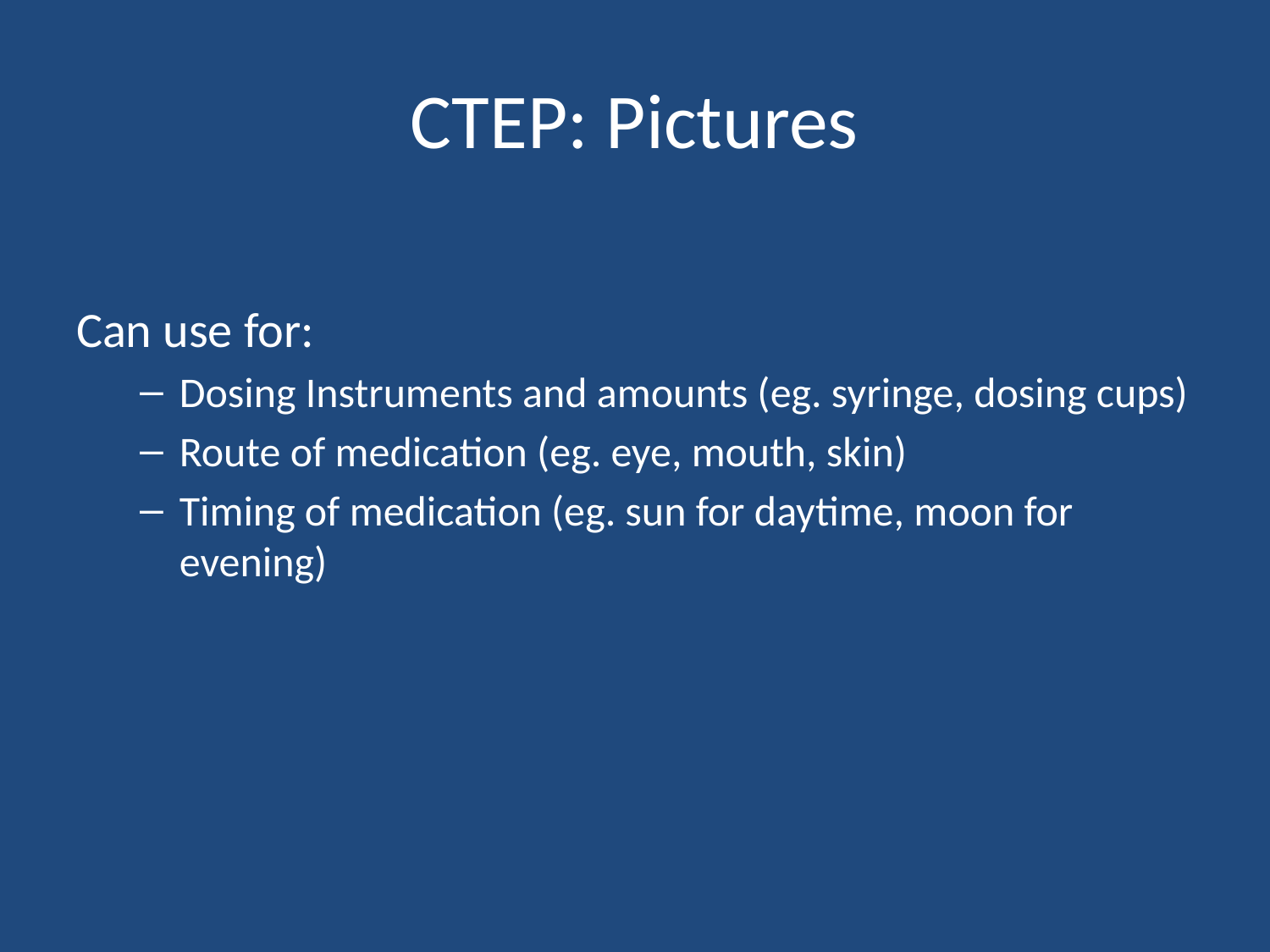

# CTEP: Pictures
Can use for:
Dosing Instruments and amounts (eg. syringe, dosing cups)
Route of medication (eg. eye, mouth, skin)
Timing of medication (eg. sun for daytime, moon for evening)

## Slide 43
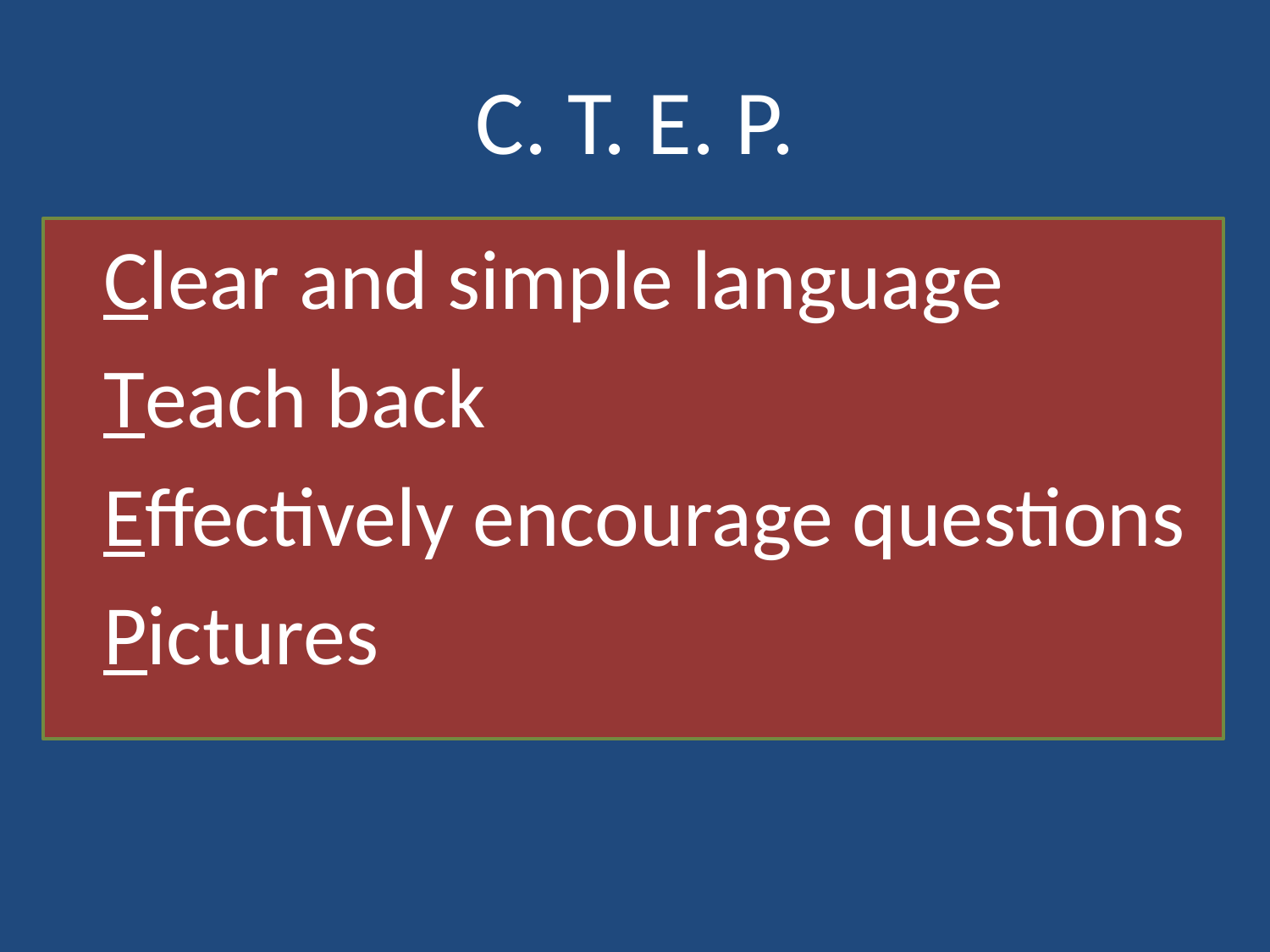

# C. T. E. P.
	Clear and simple language
	Teach back
	Effectively encourage questions
	Pictures

## Slide 44
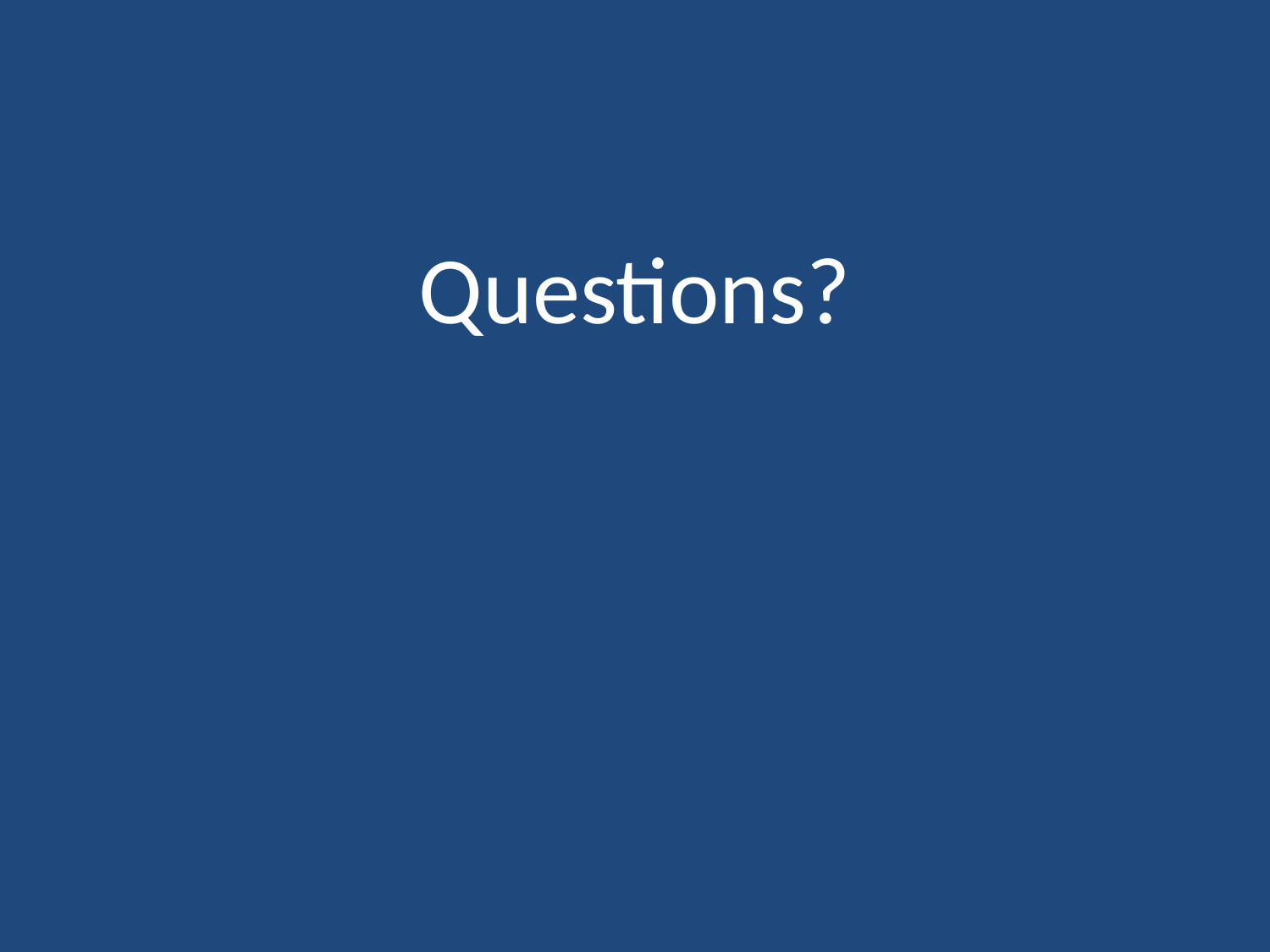

Questions?

## Slide 45
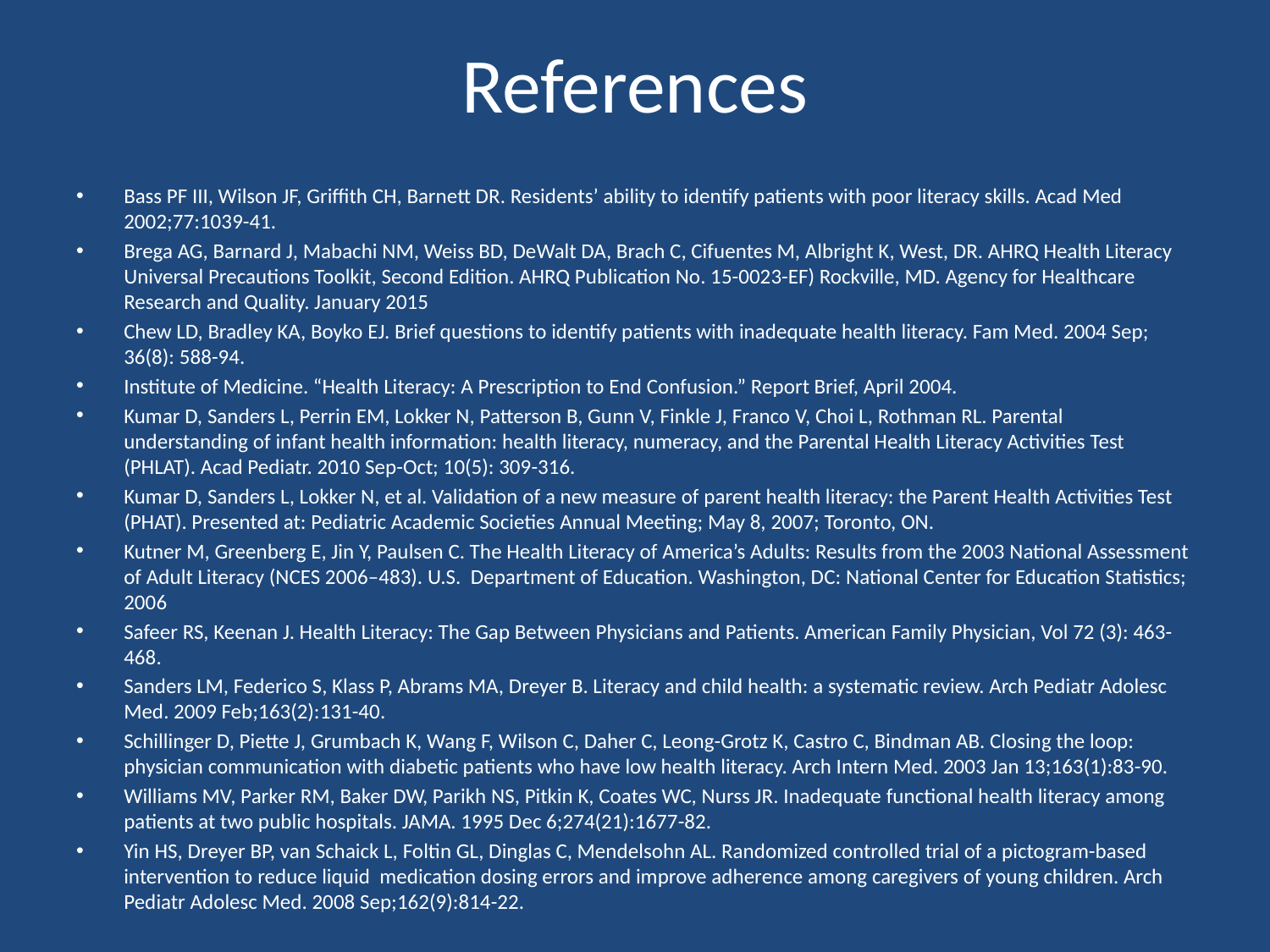

# References
Bass PF III, Wilson JF, Griffith CH, Barnett DR. Residents’ ability to identify patients with poor literacy skills. Acad Med 2002;77:1039-41.
Brega AG, Barnard J, Mabachi NM, Weiss BD, DeWalt DA, Brach C, Cifuentes M, Albright K, West, DR. AHRQ Health Literacy Universal Precautions Toolkit, Second Edition. AHRQ Publication No. 15-0023-EF) Rockville, MD. Agency for Healthcare Research and Quality. January 2015
Chew LD, Bradley KA, Boyko EJ. Brief questions to identify patients with inadequate health literacy. Fam Med. 2004 Sep; 36(8): 588-94.
Institute of Medicine. “Health Literacy: A Prescription to End Confusion.” Report Brief, April 2004.
Kumar D, Sanders L, Perrin EM, Lokker N, Patterson B, Gunn V, Finkle J, Franco V, Choi L, Rothman RL. Parental understanding of infant health information: health literacy, numeracy, and the Parental Health Literacy Activities Test (PHLAT). Acad Pediatr. 2010 Sep-Oct; 10(5): 309-316.
Kumar D, Sanders L, Lokker N, et al. Validation of a new measure of parent health literacy: the Parent Health Activities Test (PHAT). Presented at: Pediatric Academic Societies Annual Meeting; May 8, 2007; Toronto, ON.
Kutner M, Greenberg E, Jin Y, Paulsen C. The Health Literacy of America’s Adults: Results from the 2003 National Assessment of Adult Literacy (NCES 2006–483). U.S. Department of Education. Washington, DC: National Center for Education Statistics; 2006
Safeer RS, Keenan J. Health Literacy: The Gap Between Physicians and Patients. American Family Physician, Vol 72 (3): 463-468.
Sanders LM, Federico S, Klass P, Abrams MA, Dreyer B. Literacy and child health: a systematic review. Arch Pediatr Adolesc Med. 2009 Feb;163(2):131-40.
Schillinger D, Piette J, Grumbach K, Wang F, Wilson C, Daher C, Leong-Grotz K, Castro C, Bindman AB. Closing the loop: physician communication with diabetic patients who have low health literacy. Arch Intern Med. 2003 Jan 13;163(1):83-90.
Williams MV, Parker RM, Baker DW, Parikh NS, Pitkin K, Coates WC, Nurss JR. Inadequate functional health literacy among patients at two public hospitals. JAMA. 1995 Dec 6;274(21):1677-82.
Yin HS, Dreyer BP, van Schaick L, Foltin GL, Dinglas C, Mendelsohn AL. Randomized controlled trial of a pictogram-based intervention to reduce liquid medication dosing errors and improve adherence among caregivers of young children. Arch Pediatr Adolesc Med. 2008 Sep;162(9):814-22.
