# Supplementary material for: You Can Teach Every Patient: A Health Literacy and Clear Communication Curriculum for Pediatric Clerkship Students
Source: MedEdPORTAL. 2021 Jan 22;17:11086. doi: 10.15766/mep_2374-8265.11086 (PMC7821440; doi:10.15766/mep_2374-8265.11086)
Supplement: Supplementary file 1 — HLCC Didactic PowerPoint.pptxWorkshop PowerPoint.pptxCTEP Card.docxVideo for Critique.m4vClear Language Cases Students.docxClear Language Cases Instructors Guide.docxTeach-back Cases Students.docxTeach-back Cases Instructors Guide.docxPicture Cases Students.docxPicture Cases Instructors Guide.docxCTEP Cases Students.docxCTEP Cases Instructors Guide.docxCommunication Checklist.docxStudent Survey.docx [file mep_2374-8265.11086-s001.zip › B. Workshop PowerPoint.pptx]

## Slide 1
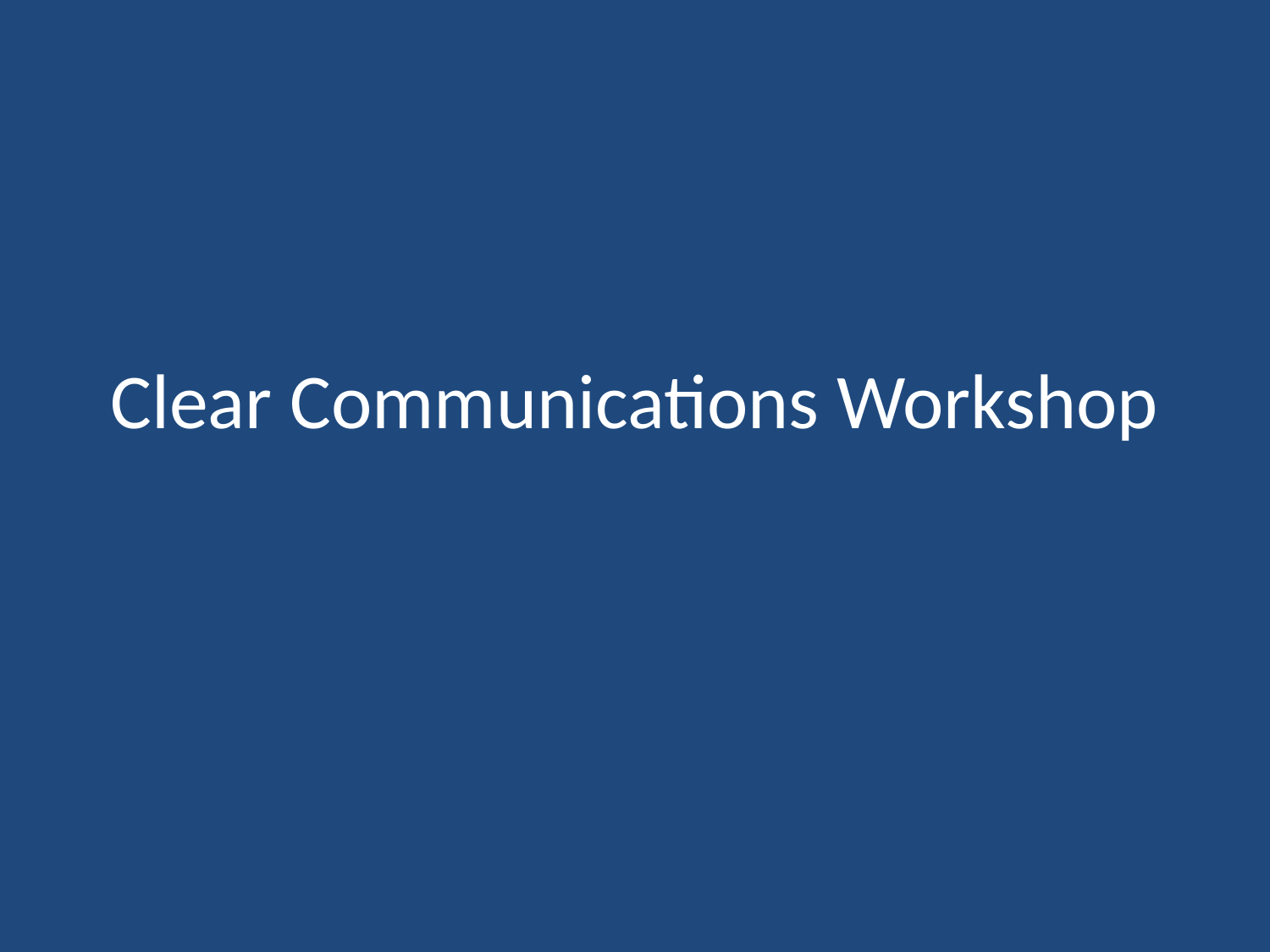

# Clear Communications Workshop

## Slide 2
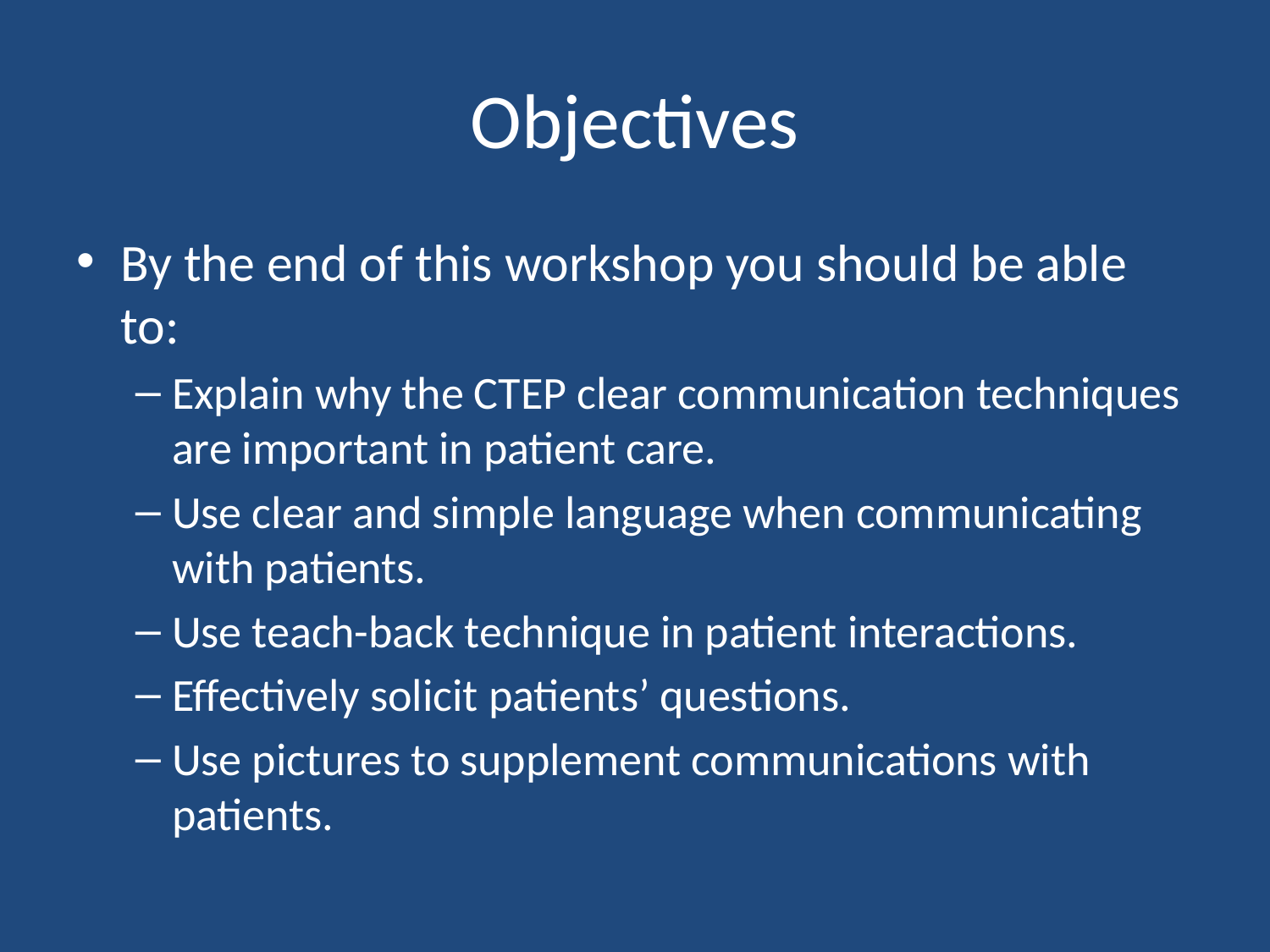

# Objectives
By the end of this workshop you should be able to:
Explain why the CTEP clear communication techniques are important in patient care.
Use clear and simple language when communicating with patients.
Use teach-back technique in patient interactions.
Effectively solicit patients’ questions.
Use pictures to supplement communications with patients.

## Slide 3
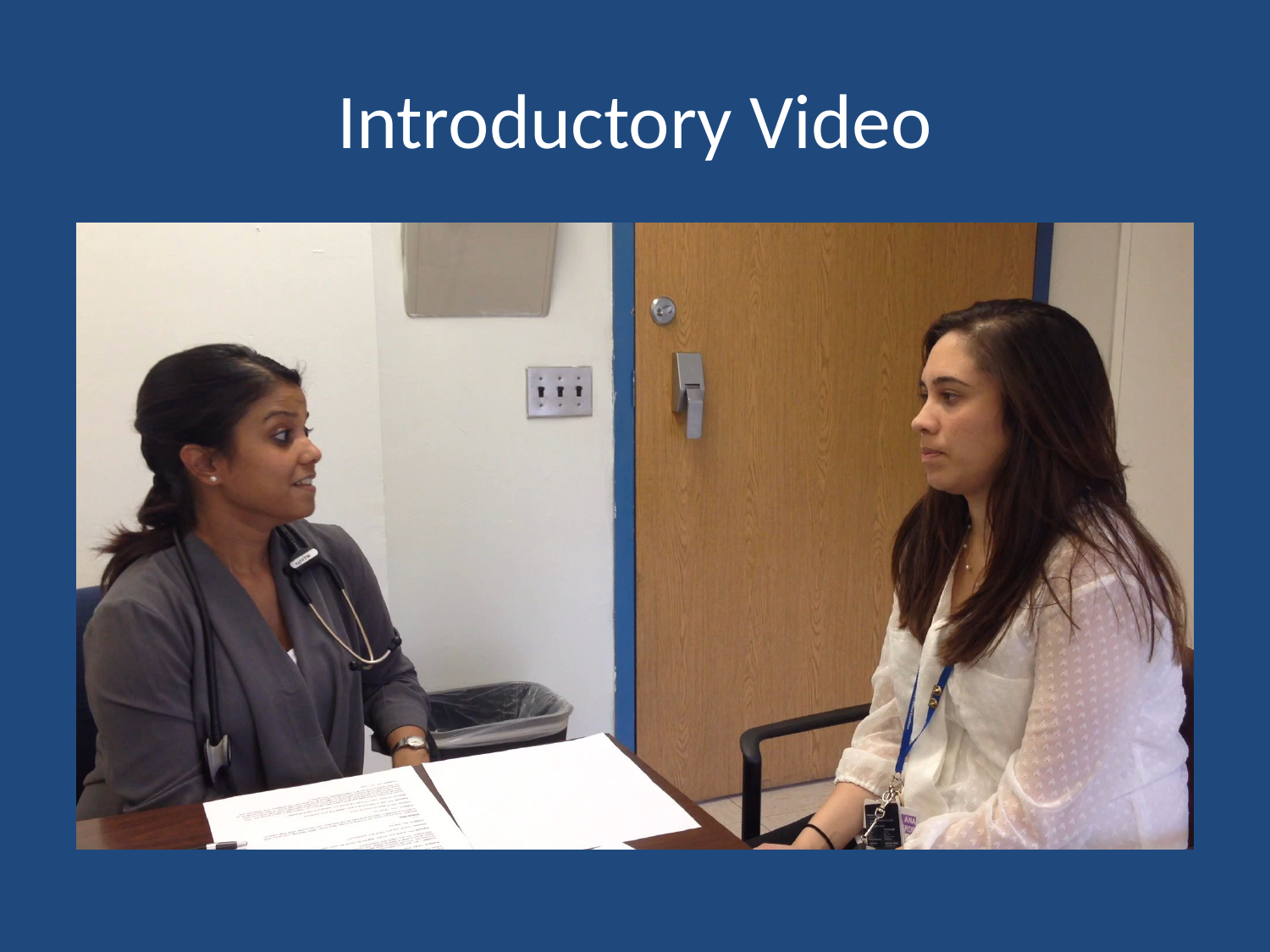

# Introductory Video

## Slide 4
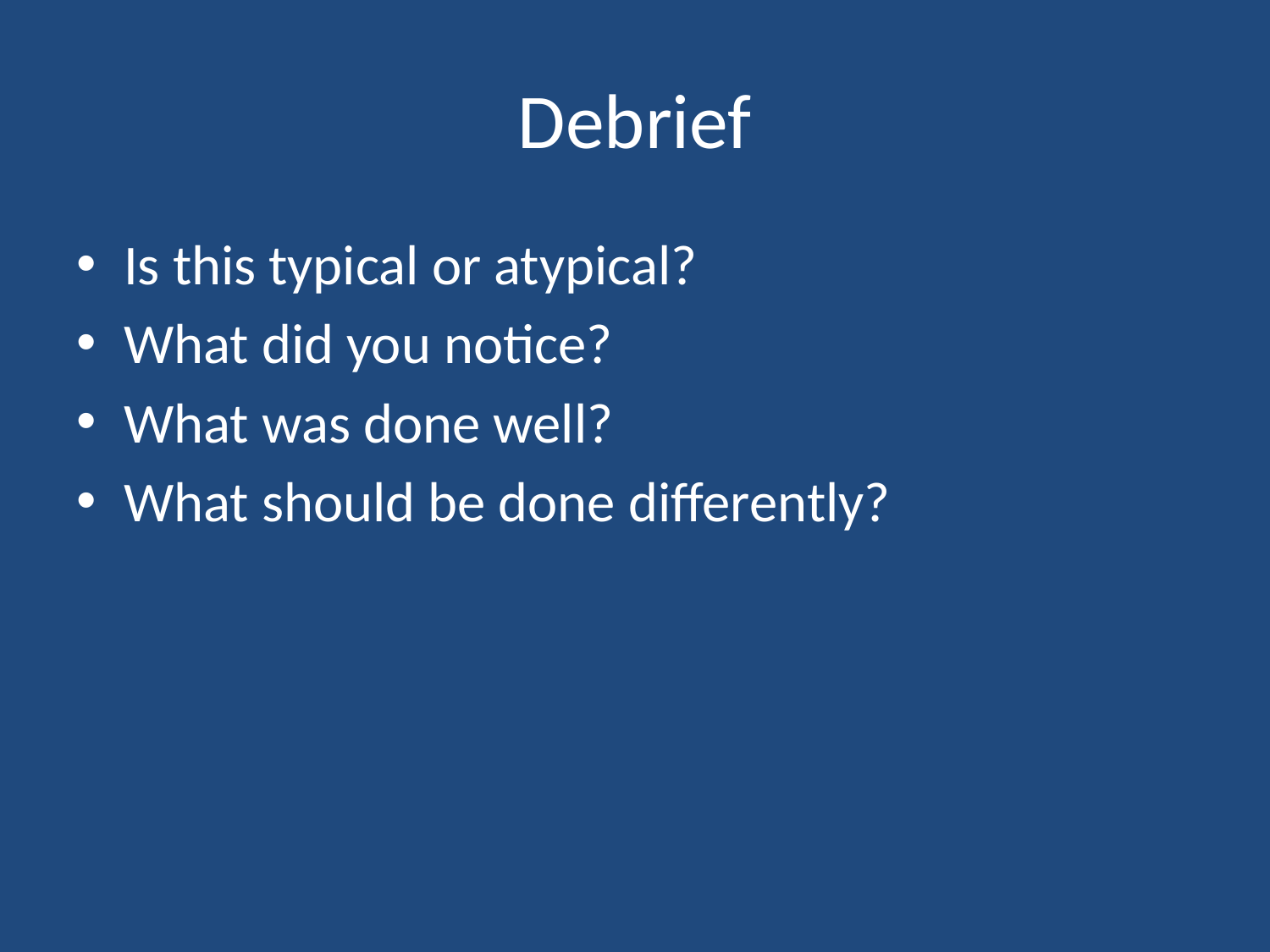

# Debrief
Is this typical or atypical?
What did you notice?
What was done well?
What should be done differently?

## Slide 5
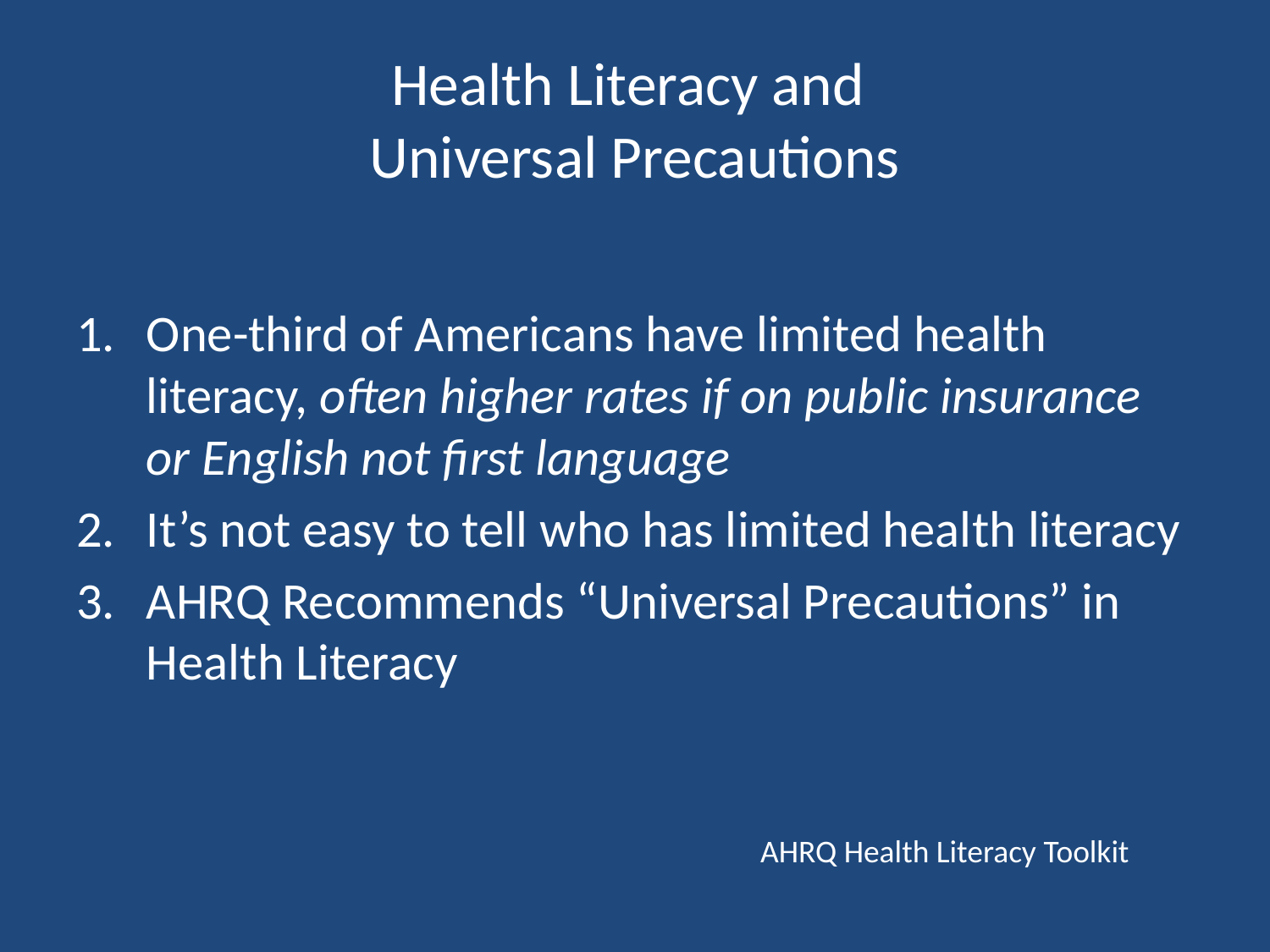

# Health Literacy and Universal Precautions
One-third of Americans have limited health literacy, often higher rates if on public insurance or English not first language
It’s not easy to tell who has limited health literacy
AHRQ Recommends “Universal Precautions” in Health Literacy
AHRQ Health Literacy Toolkit

## Slide 6
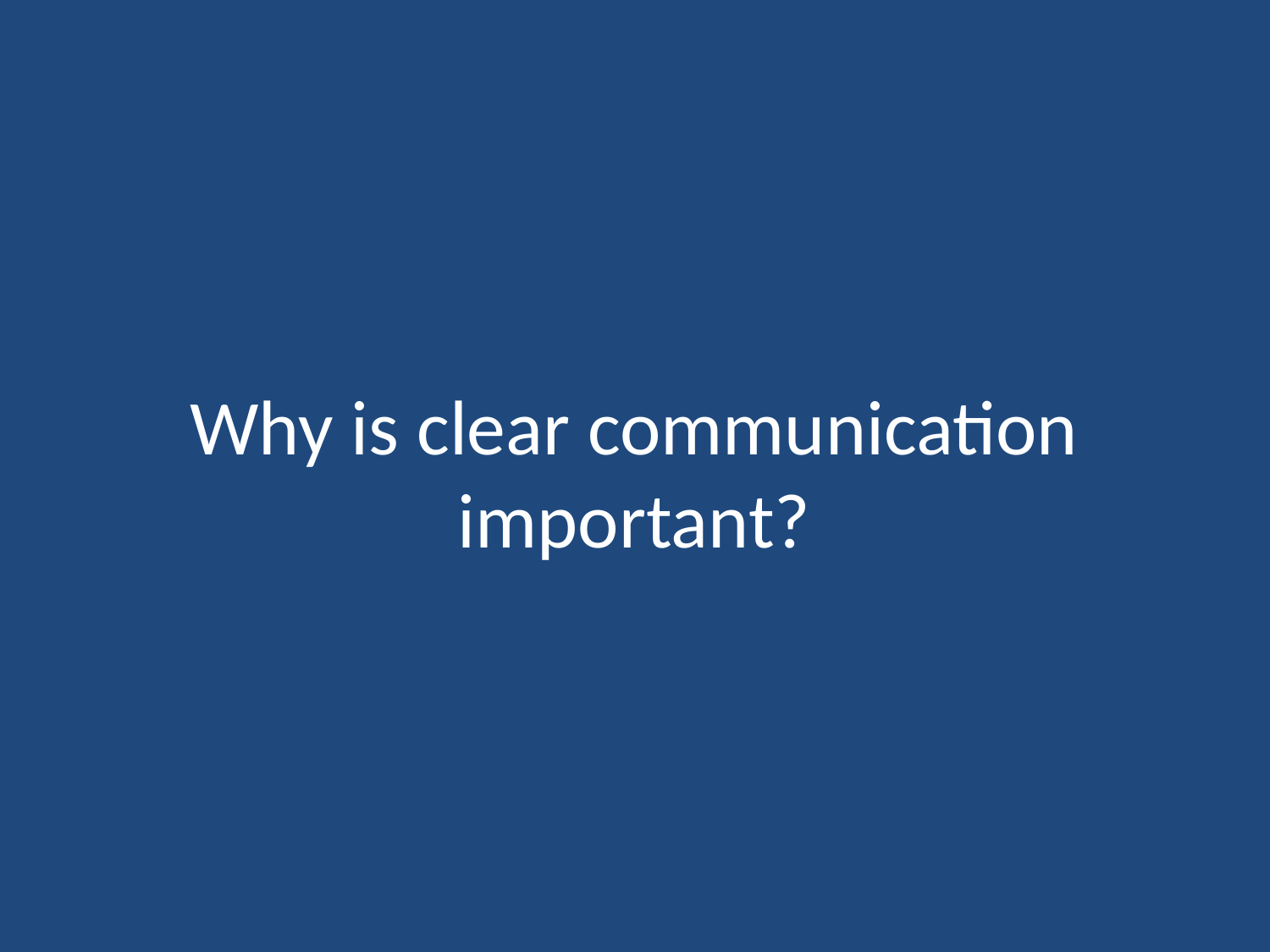

# Why is clear communication important?

## Slide 7
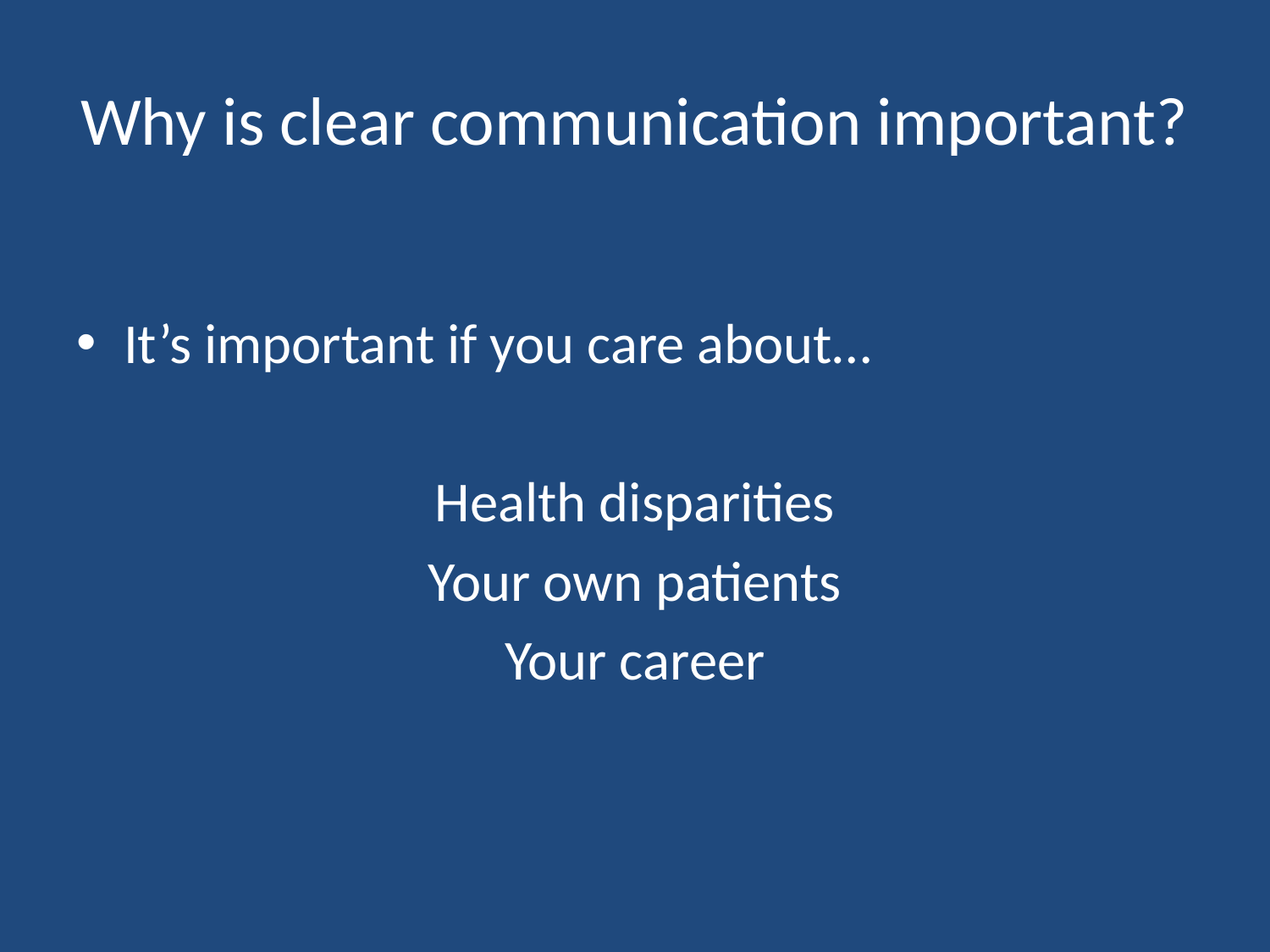

# Why is clear communication important?
It’s important if you care about…
Health disparities
Your own patients
Your career

## Slide 8
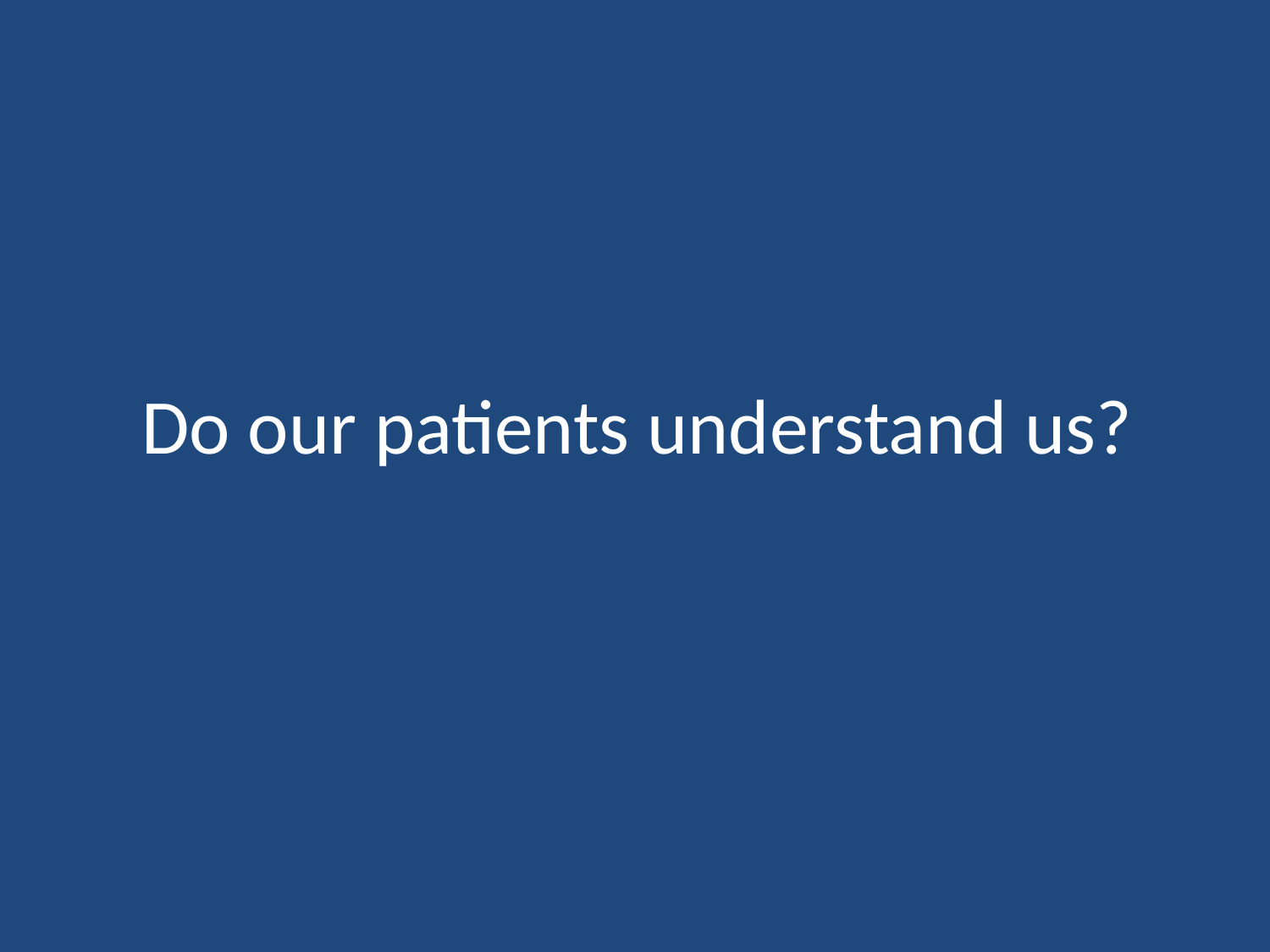

# Do our patients understand us?

## Slide 9
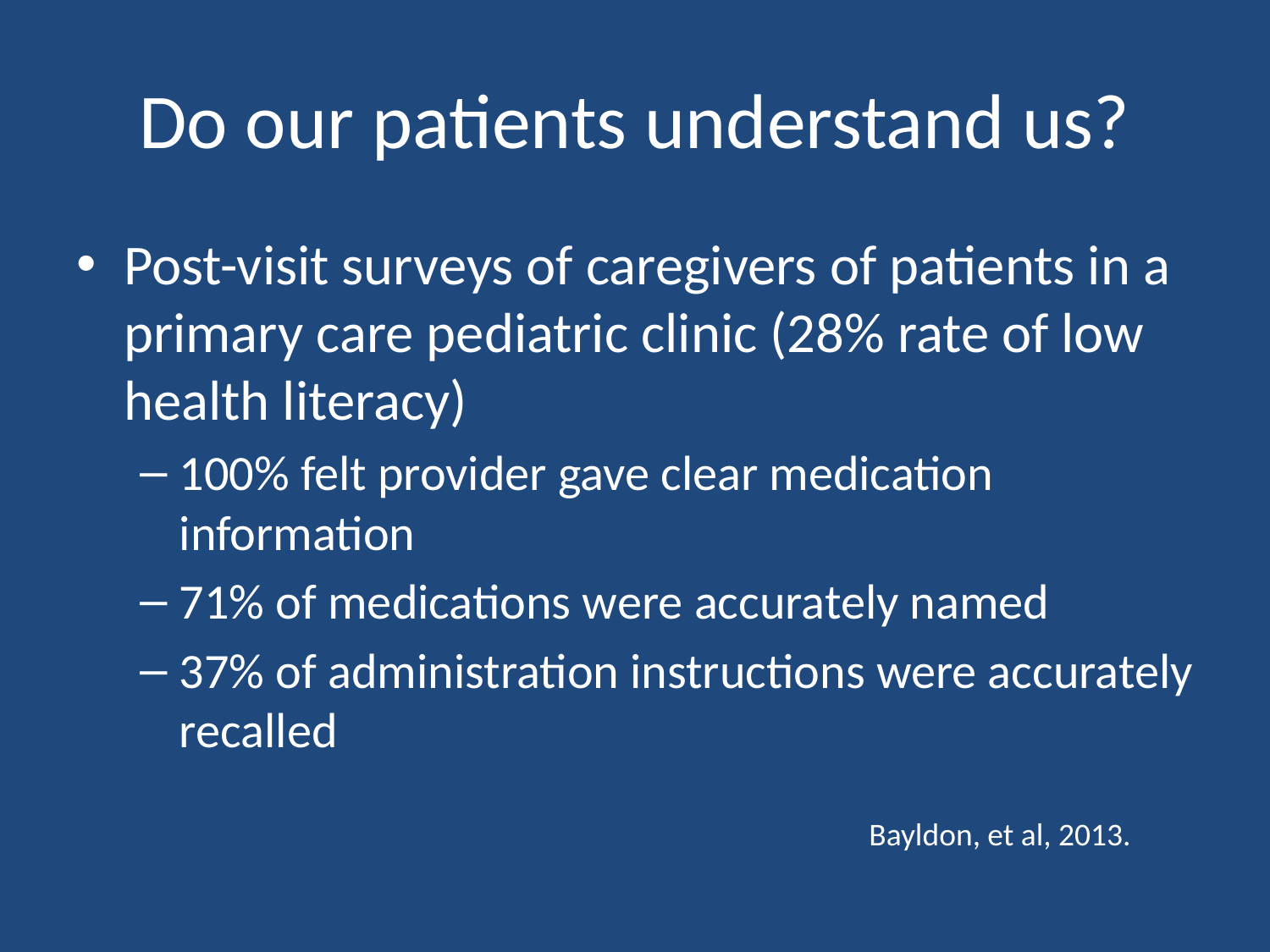

# Do our patients understand us?
Post-visit surveys of caregivers of patients in a primary care pediatric clinic (28% rate of low health literacy)
100% felt provider gave clear medication information
71% of medications were accurately named
37% of administration instructions were accurately recalled
Bayldon, et al, 2013.

## Slide 10
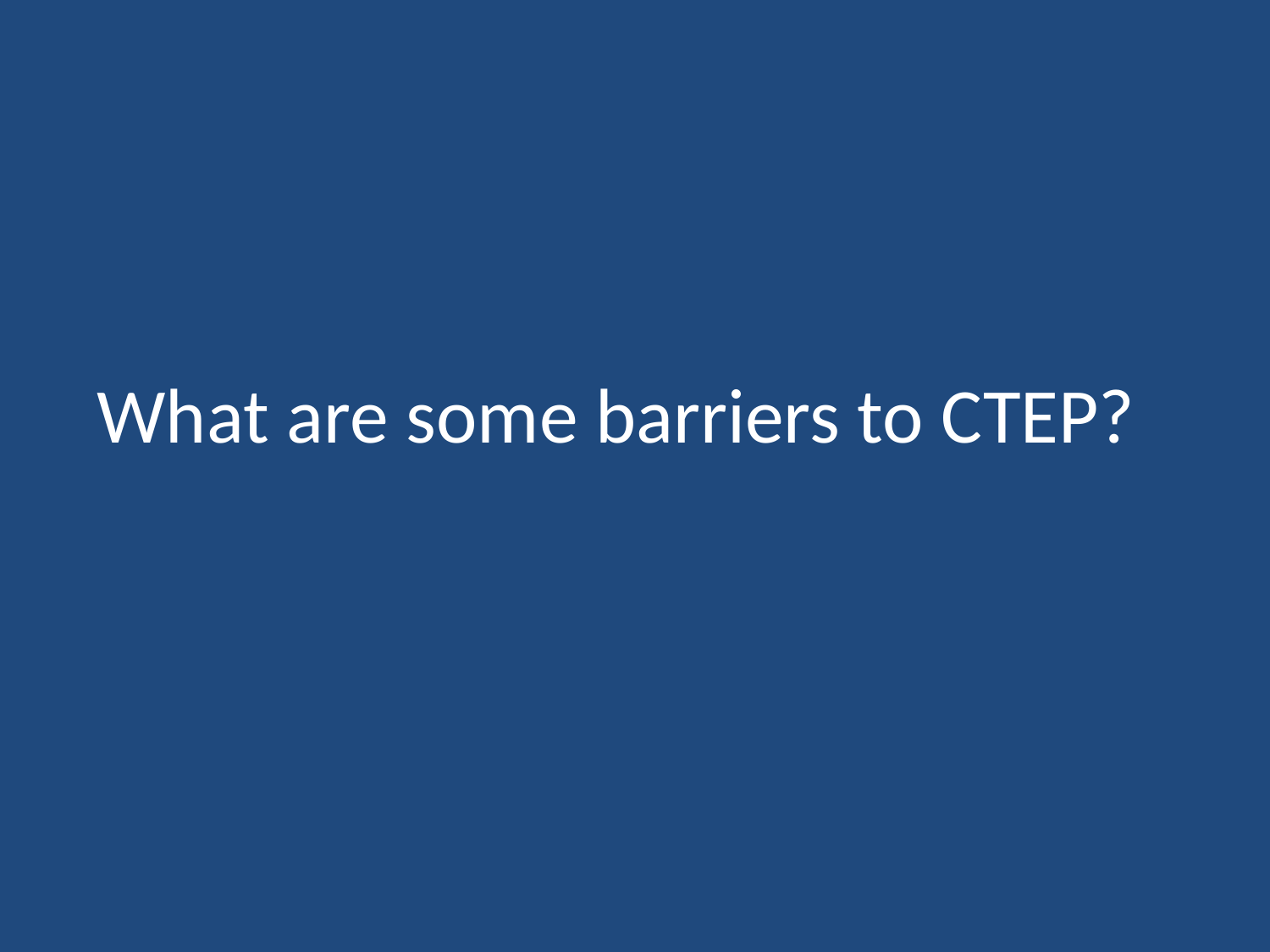

# What are some barriers to CTEP?

## Slide 11
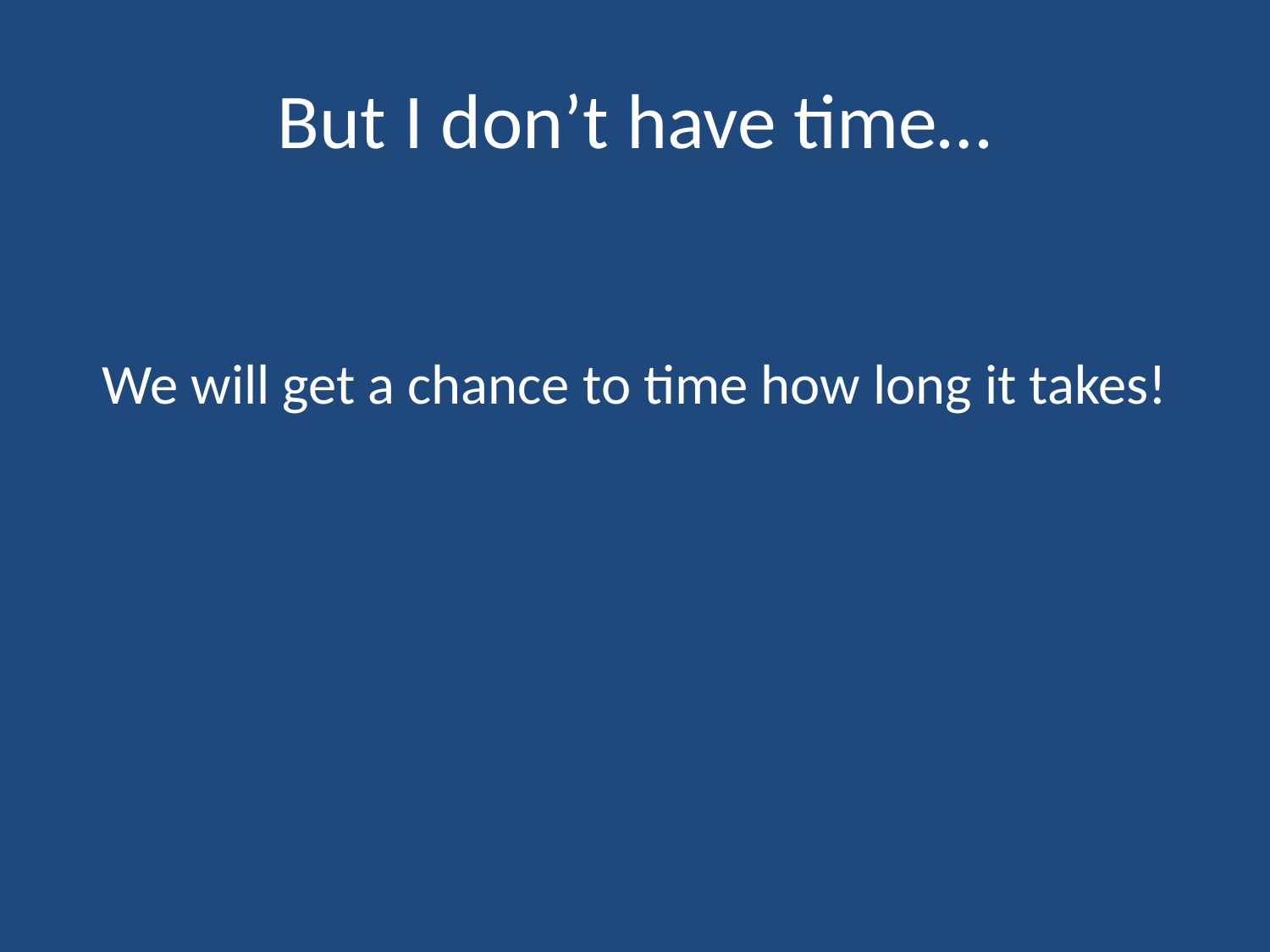

# But I don’t have time…
We will get a chance to time how long it takes!

## Slide 12
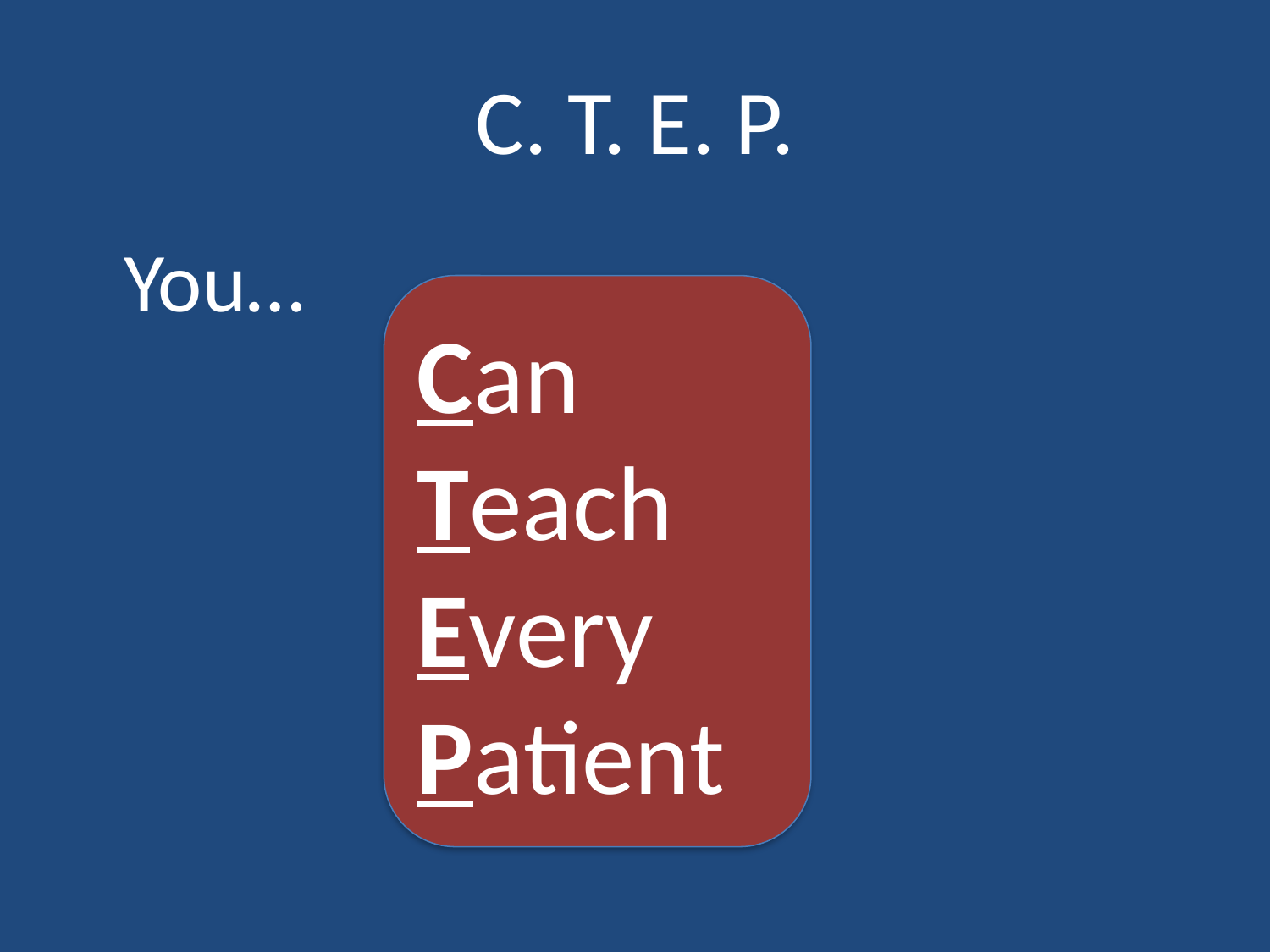

# C. T. E. P.
	You…
Can
Teach
Every
Patient

## Slide 13
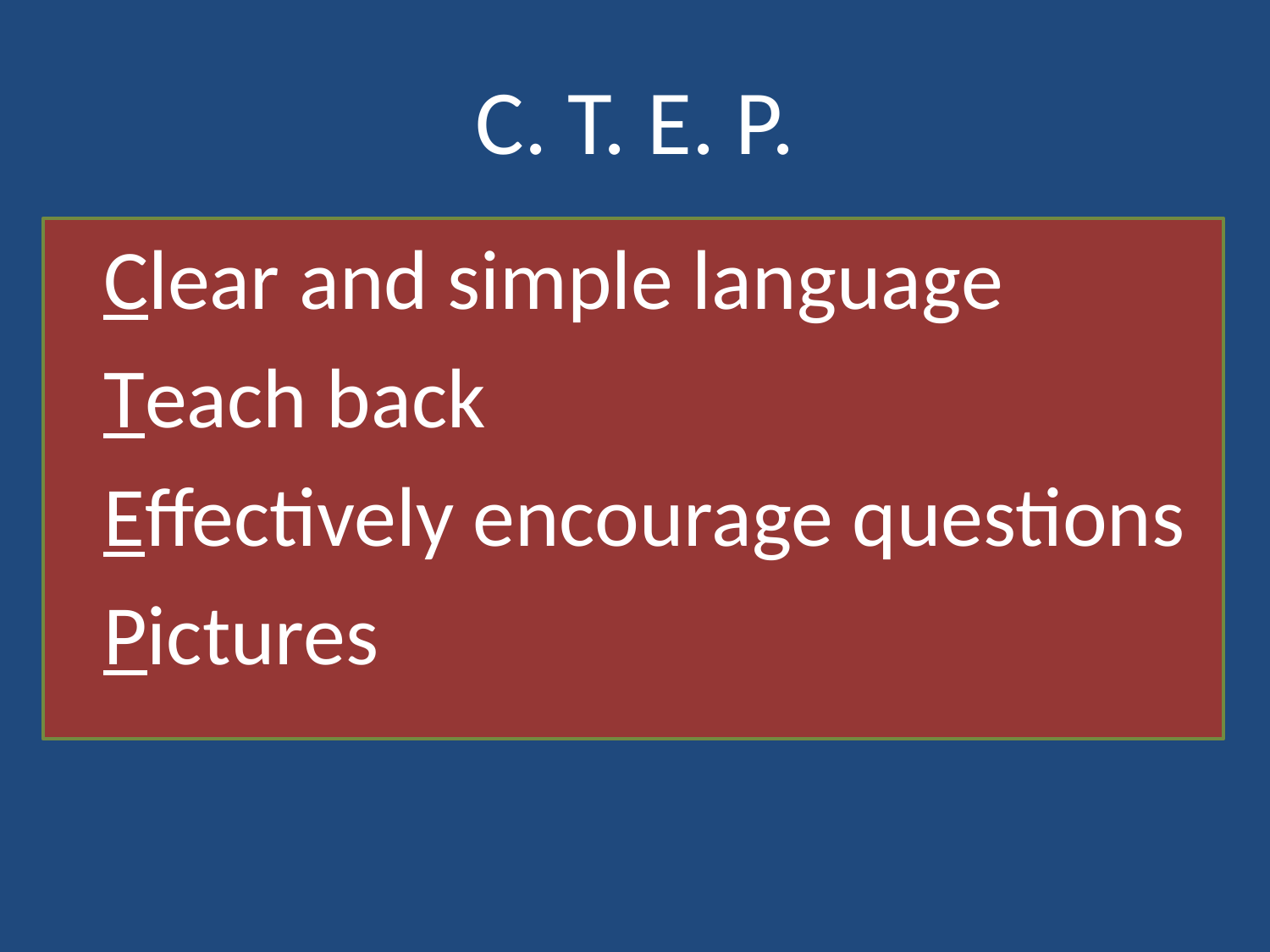

# C. T. E. P.
	Clear and simple language
	Teach back
	Effectively encourage questions
	Pictures

## Slide 14
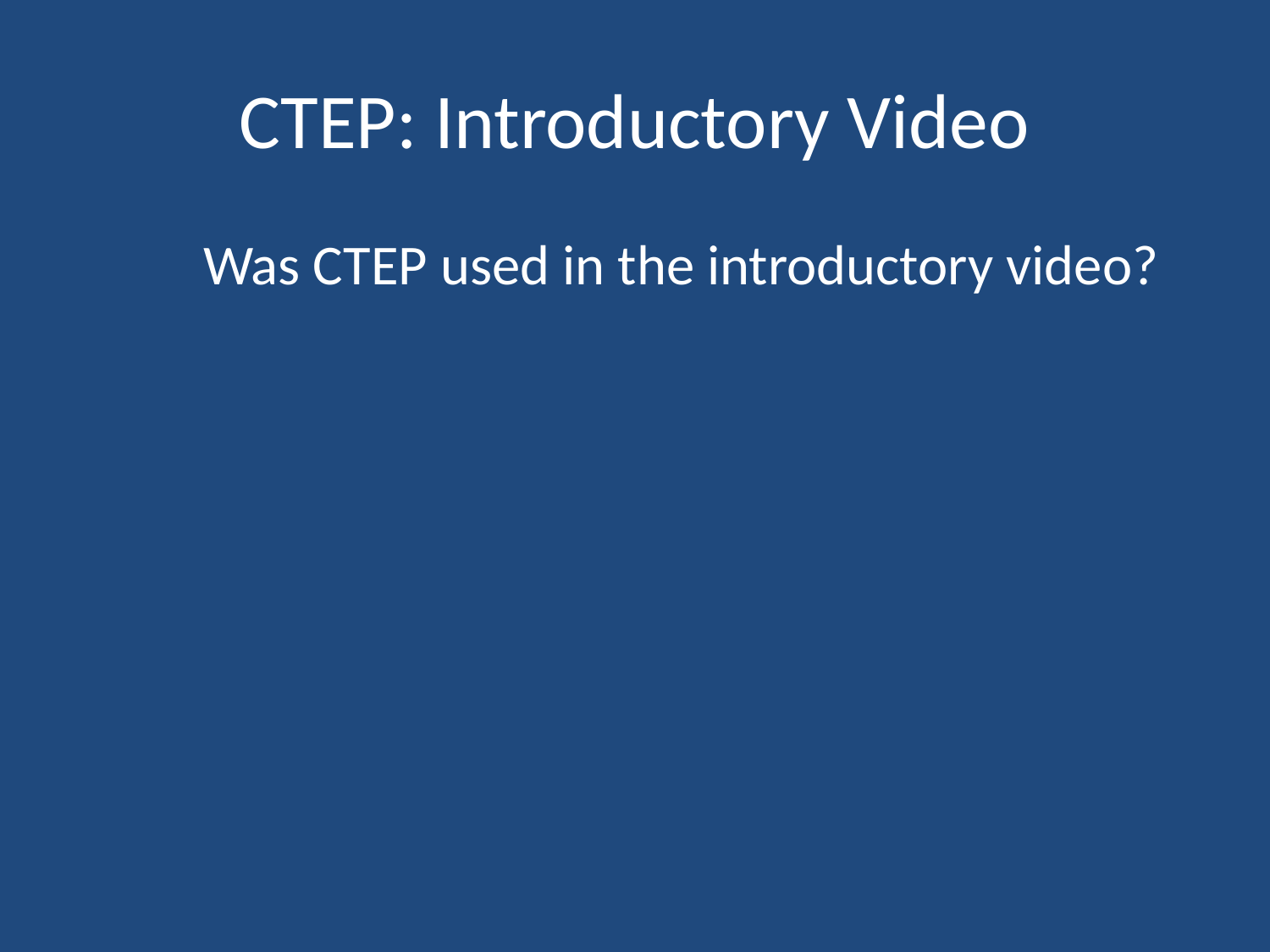

# CTEP: Introductory Video
	Was CTEP used in the introductory video?

## Slide 15
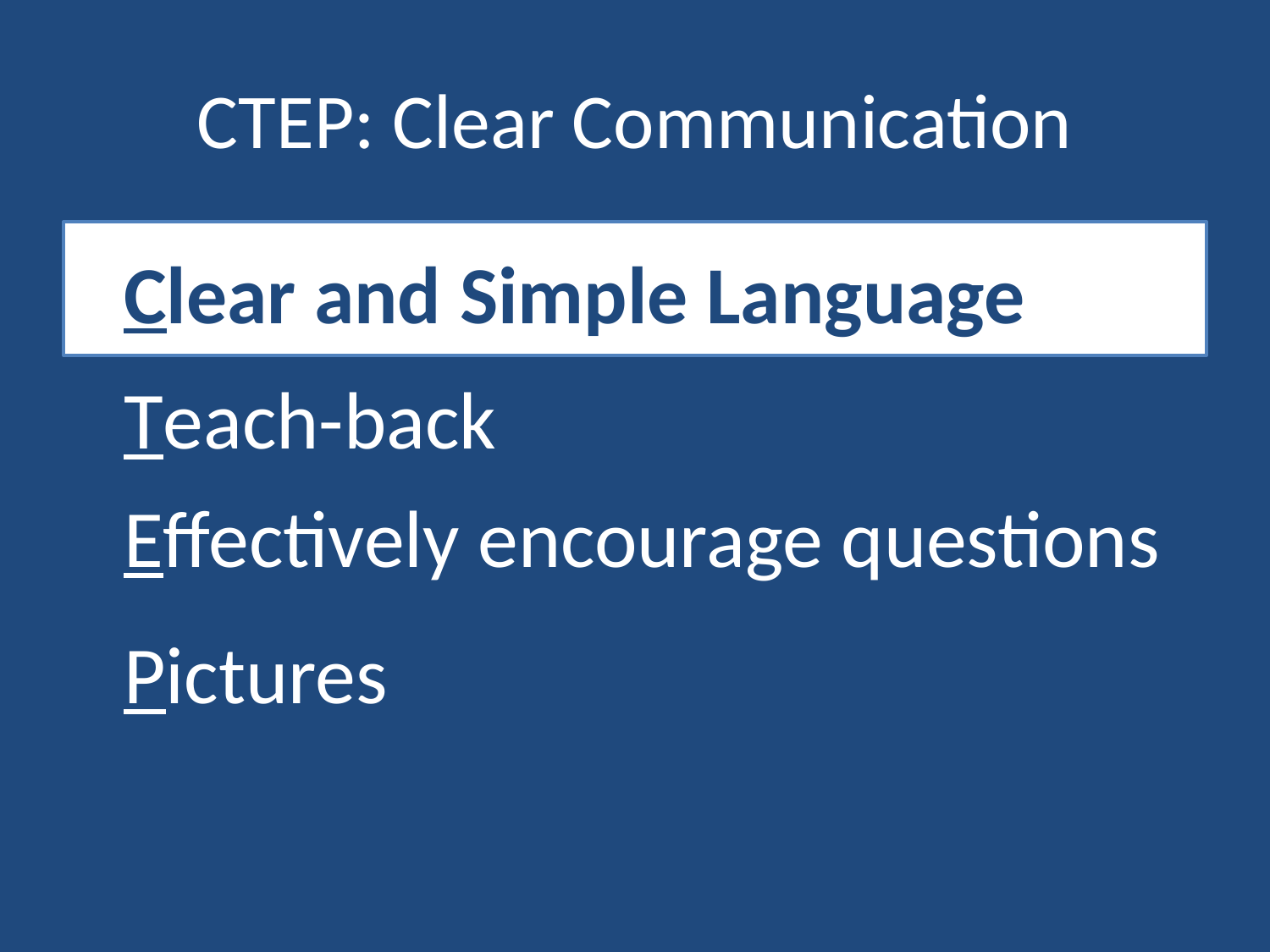

# CTEP: Clear Communication
	Clear and Simple Language
	Teach-back
	Effectively encourage questions
	Pictures

## Slide 16
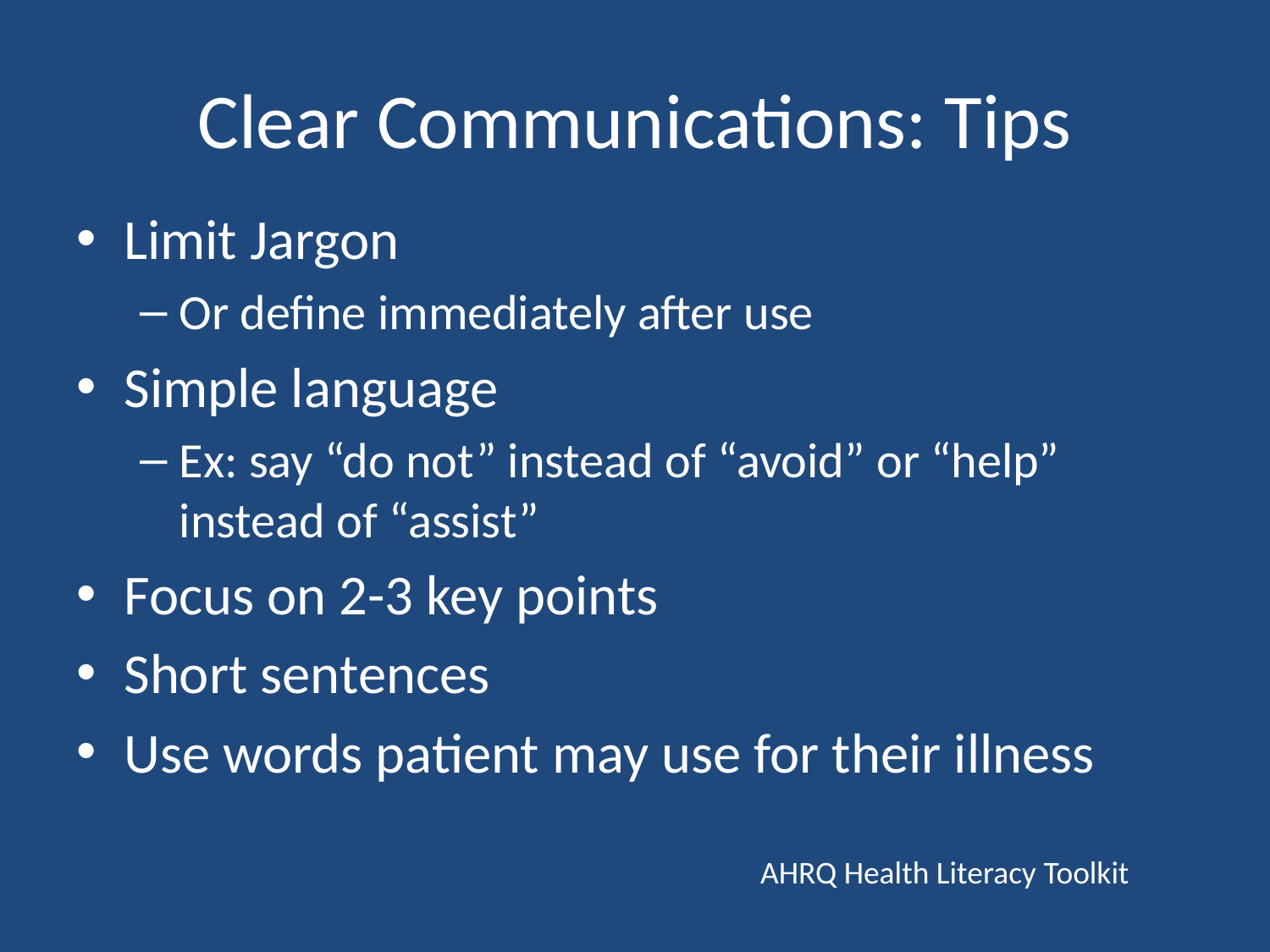

# Clear Communications: Tips
Limit Jargon
Or define immediately after use
Simple language
Ex: say “do not” instead of “avoid” or “help” instead of “assist”
Focus on 2-3 key points
Short sentences
Use words patient may use for their illness
AHRQ Health Literacy Toolkit

## Slide 17
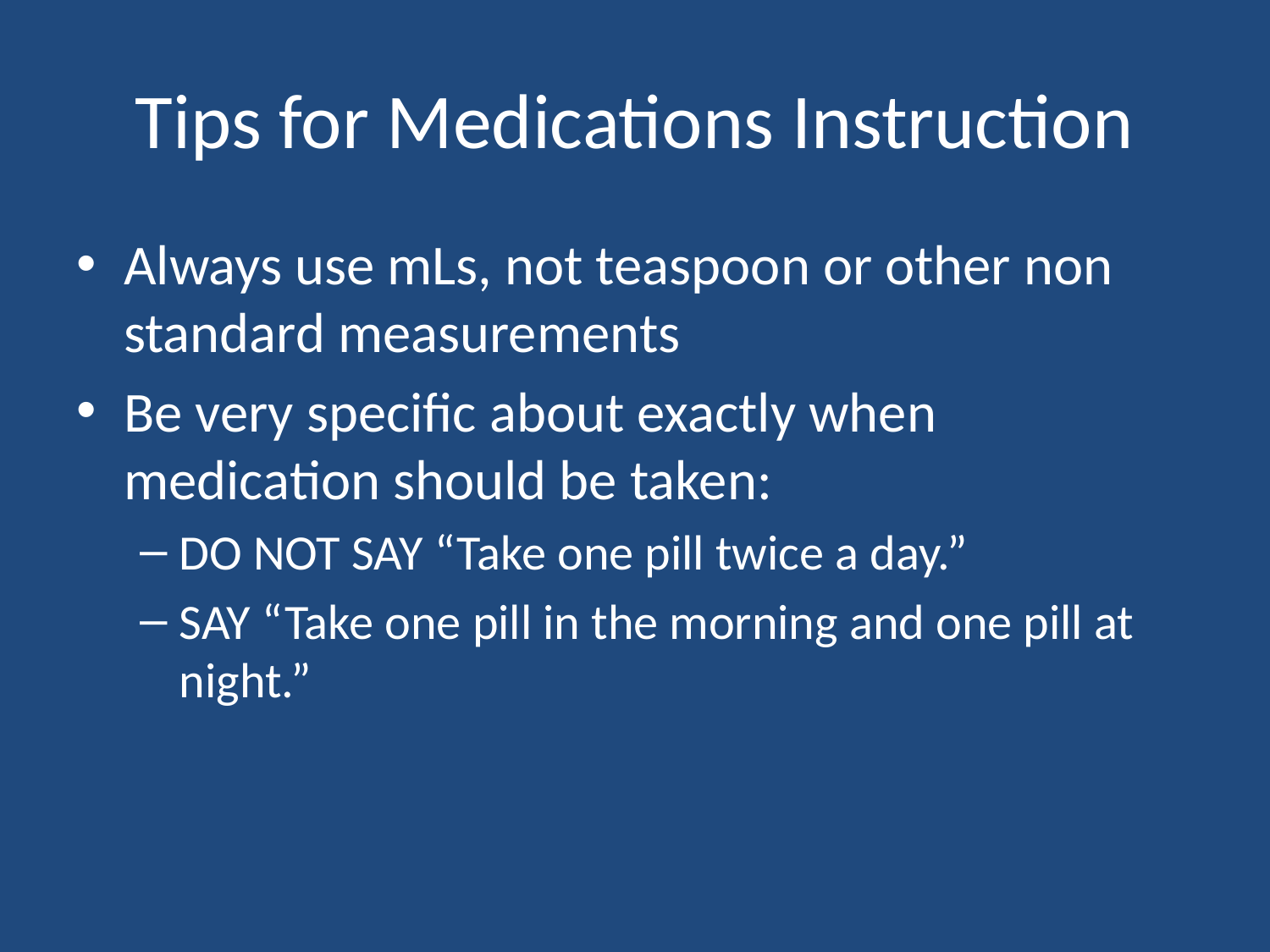

# Tips for Medications Instruction
Always use mLs, not teaspoon or other non standard measurements
Be very specific about exactly when medication should be taken:
DO NOT SAY “Take one pill twice a day.”
SAY “Take one pill in the morning and one pill at night.”

## Slide 18
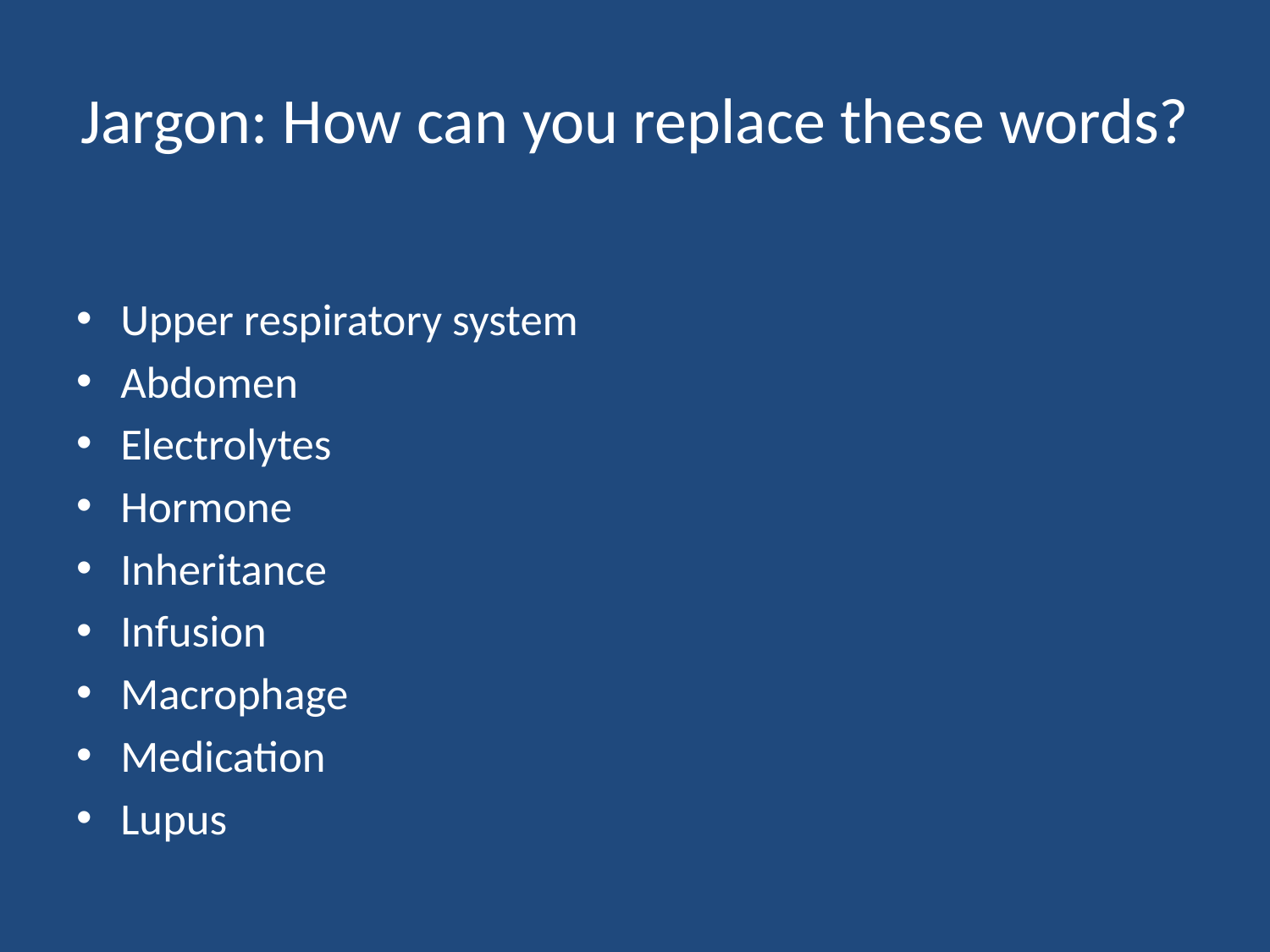

# Jargon: How can you replace these words?
Upper respiratory system
Abdomen
Electrolytes
Hormone
Inheritance
Infusion
Macrophage
Medication
Lupus

## Slide 19
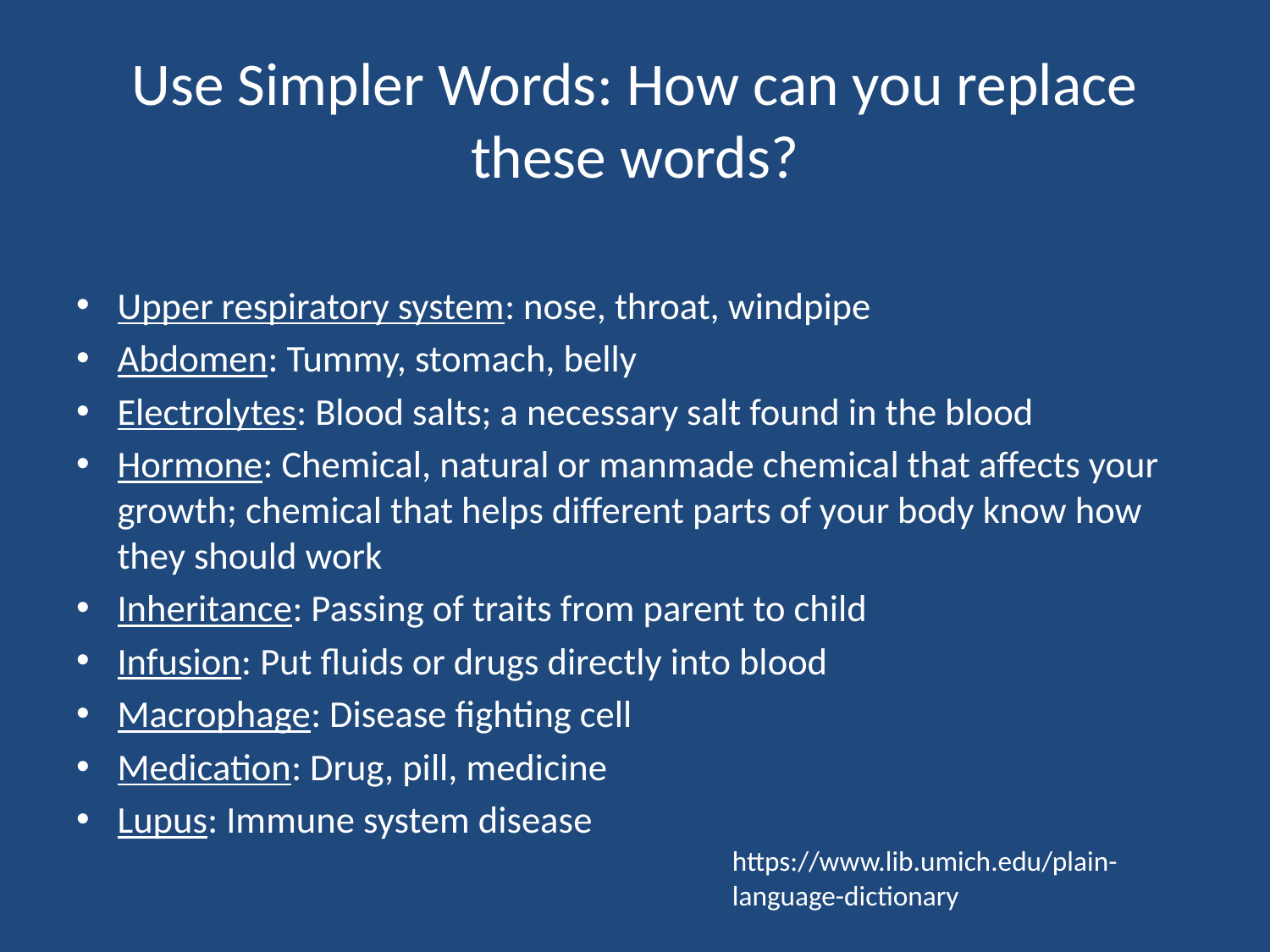

# Use Simpler Words: How can you replace these words?
Upper respiratory system: nose, throat, windpipe
Abdomen: Tummy, stomach, belly
Electrolytes: Blood salts; a necessary salt found in the blood
Hormone: Chemical, natural or manmade chemical that affects your growth; chemical that helps different parts of your body know how they should work
Inheritance: Passing of traits from parent to child
Infusion: Put fluids or drugs directly into blood
Macrophage: Disease fighting cell
Medication: Drug, pill, medicine
Lupus: Immune system disease
https://www.lib.umich.edu/plain-language-dictionary

## Slide 20
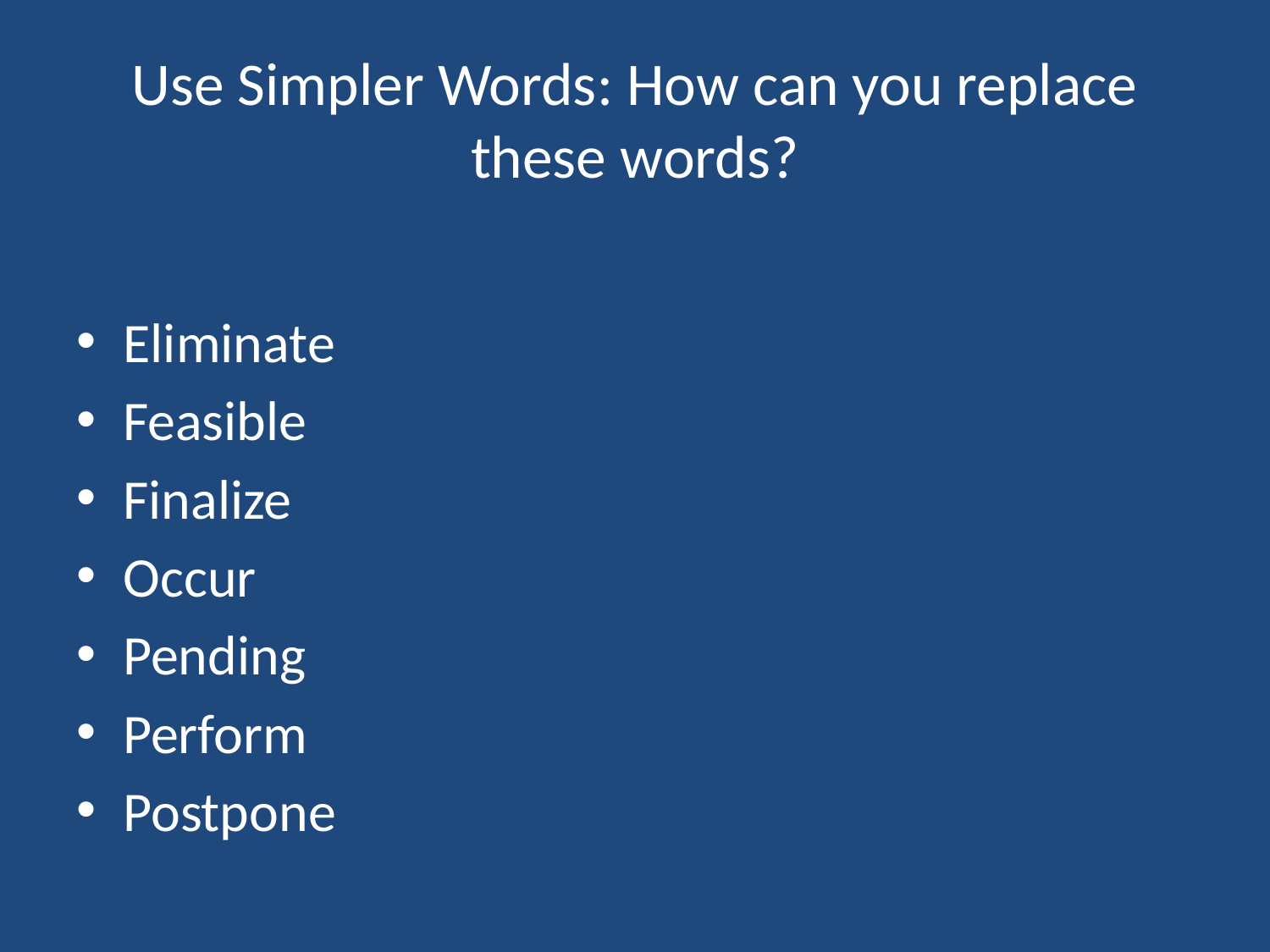

# Use Simpler Words: How can you replace these words?
Eliminate
Feasible
Finalize
Occur
Pending
Perform
Postpone

## Slide 21
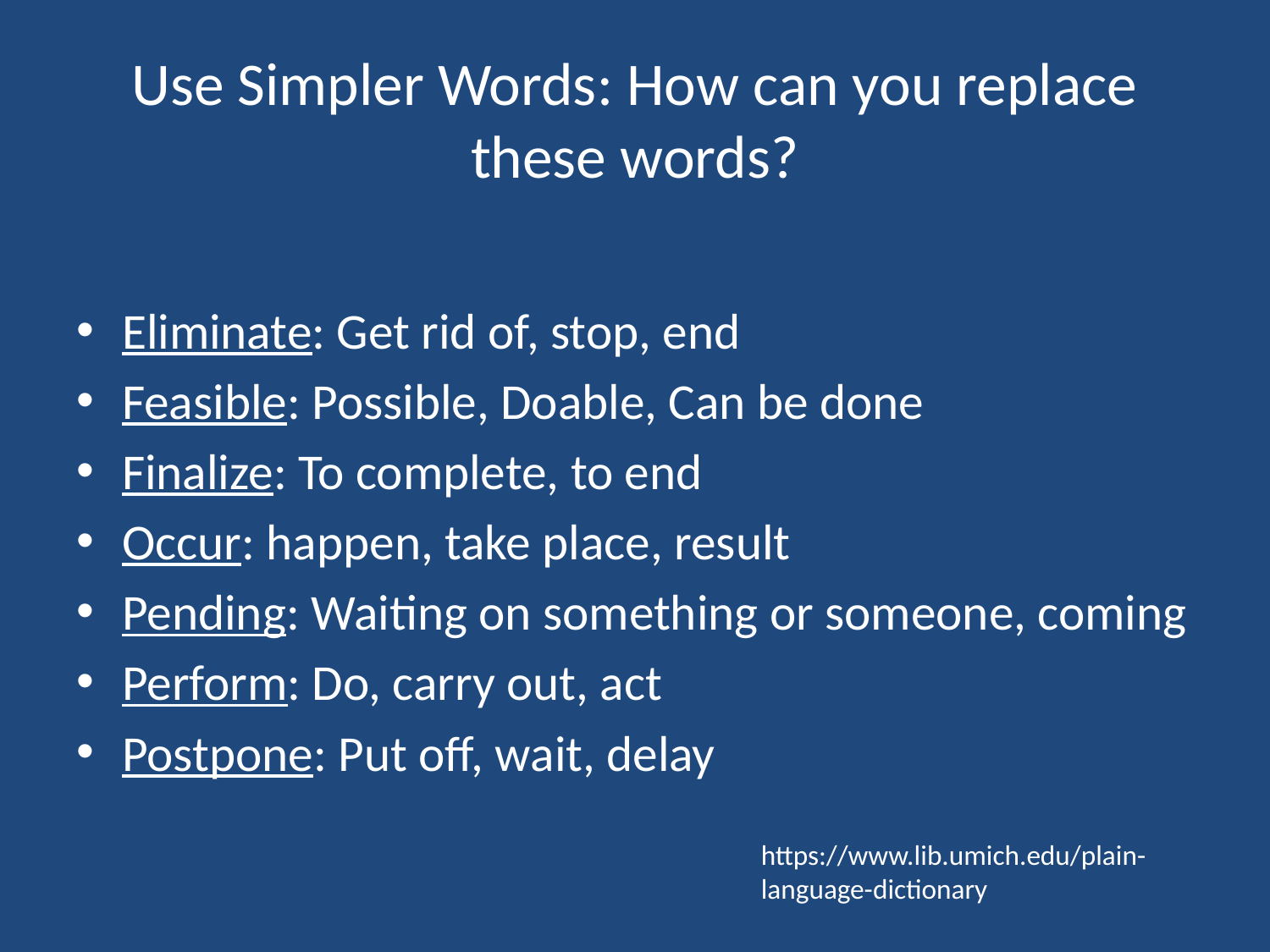

# Use Simpler Words: How can you replace these words?
Eliminate: Get rid of, stop, end
Feasible: Possible, Doable, Can be done
Finalize: To complete, to end
Occur: happen, take place, result
Pending: Waiting on something or someone, coming
Perform: Do, carry out, act
Postpone: Put off, wait, delay
https://www.lib.umich.edu/plain-language-dictionary

## Slide 22
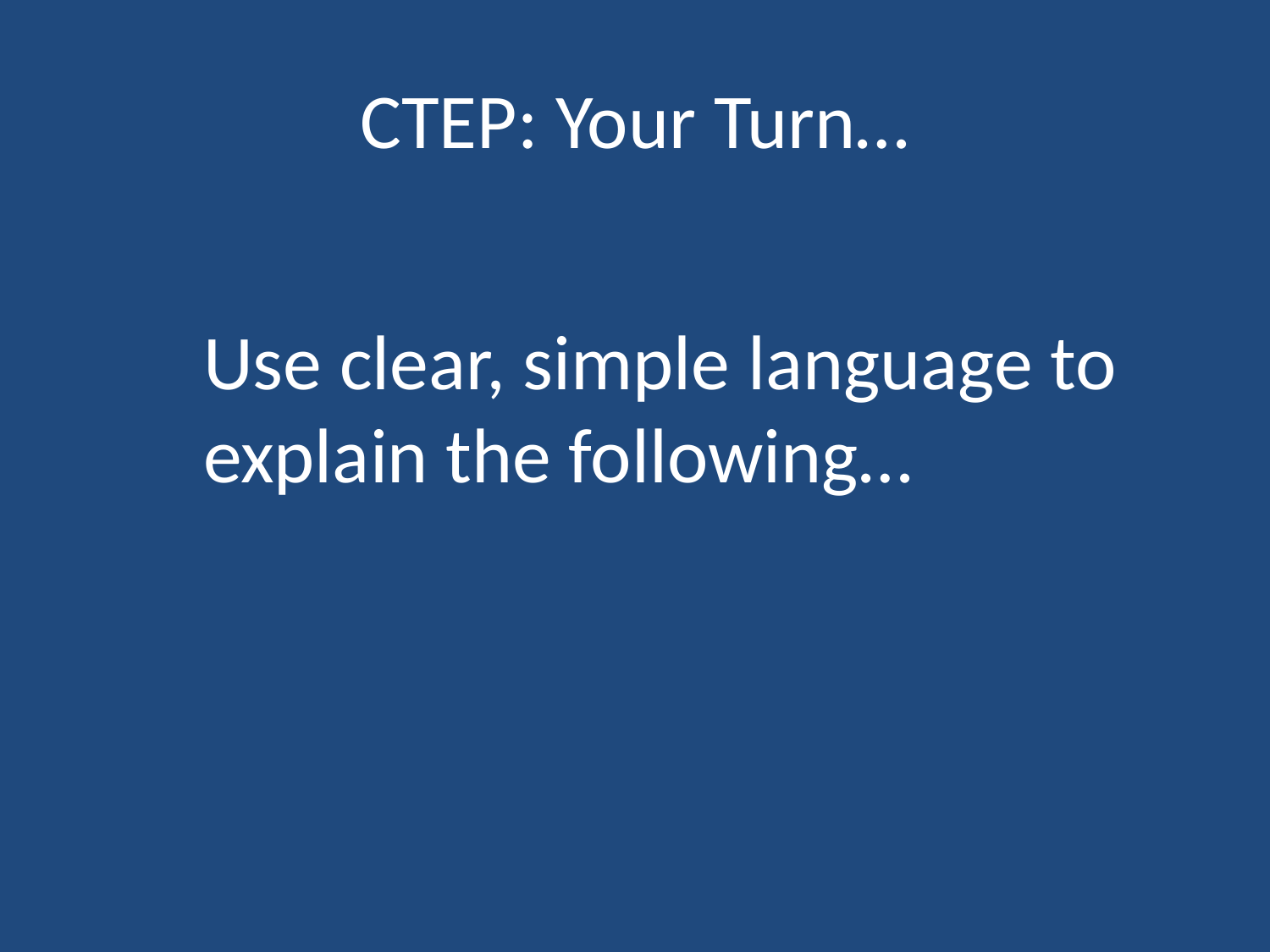

# CTEP: Your Turn…
	Use clear, simple language to 	explain the following…

## Slide 23
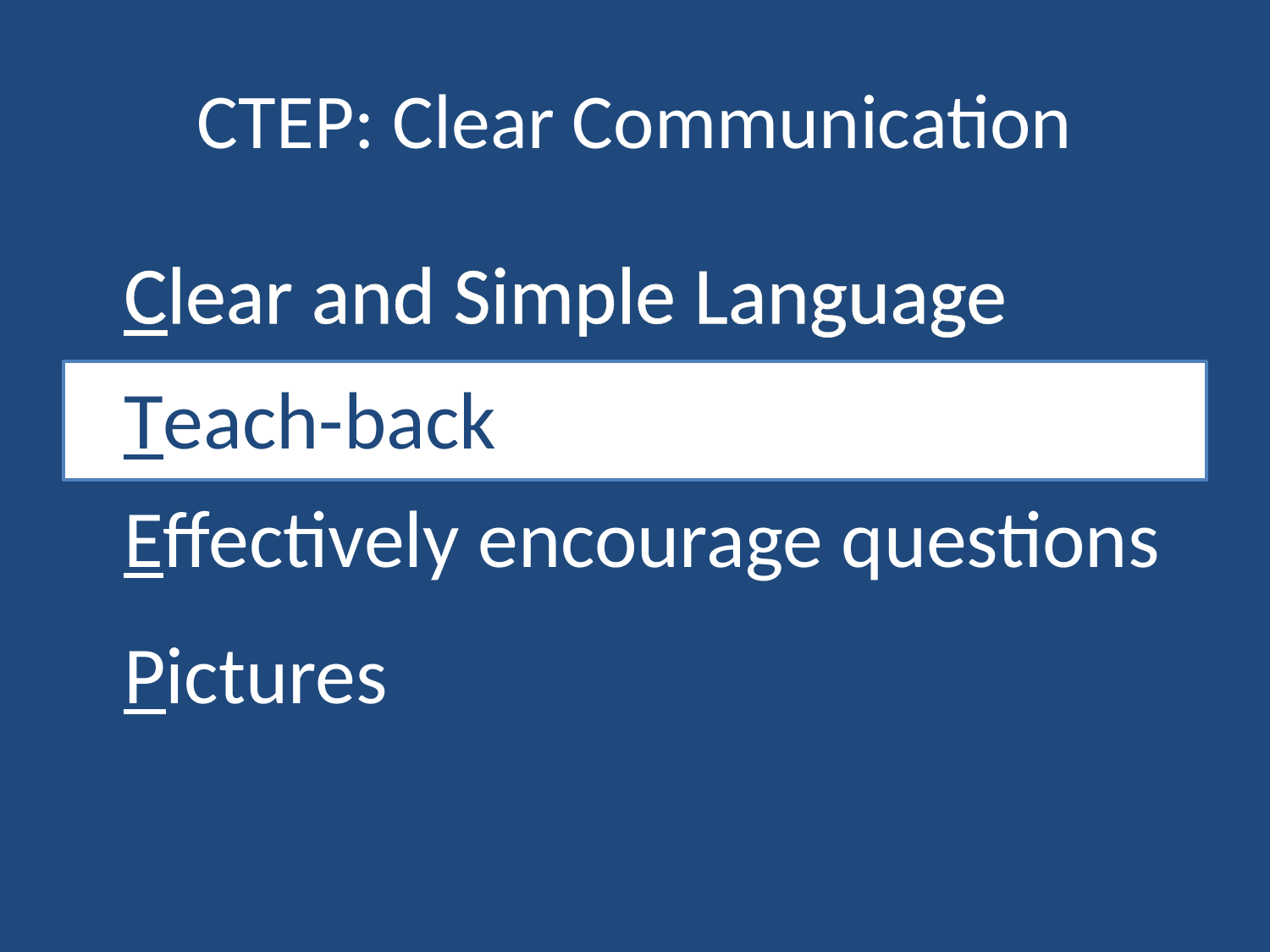

# CTEP: Clear Communication
	Clear and Simple Language
	Teach-back
	Effectively encourage questions
	Pictures

## Slide 24
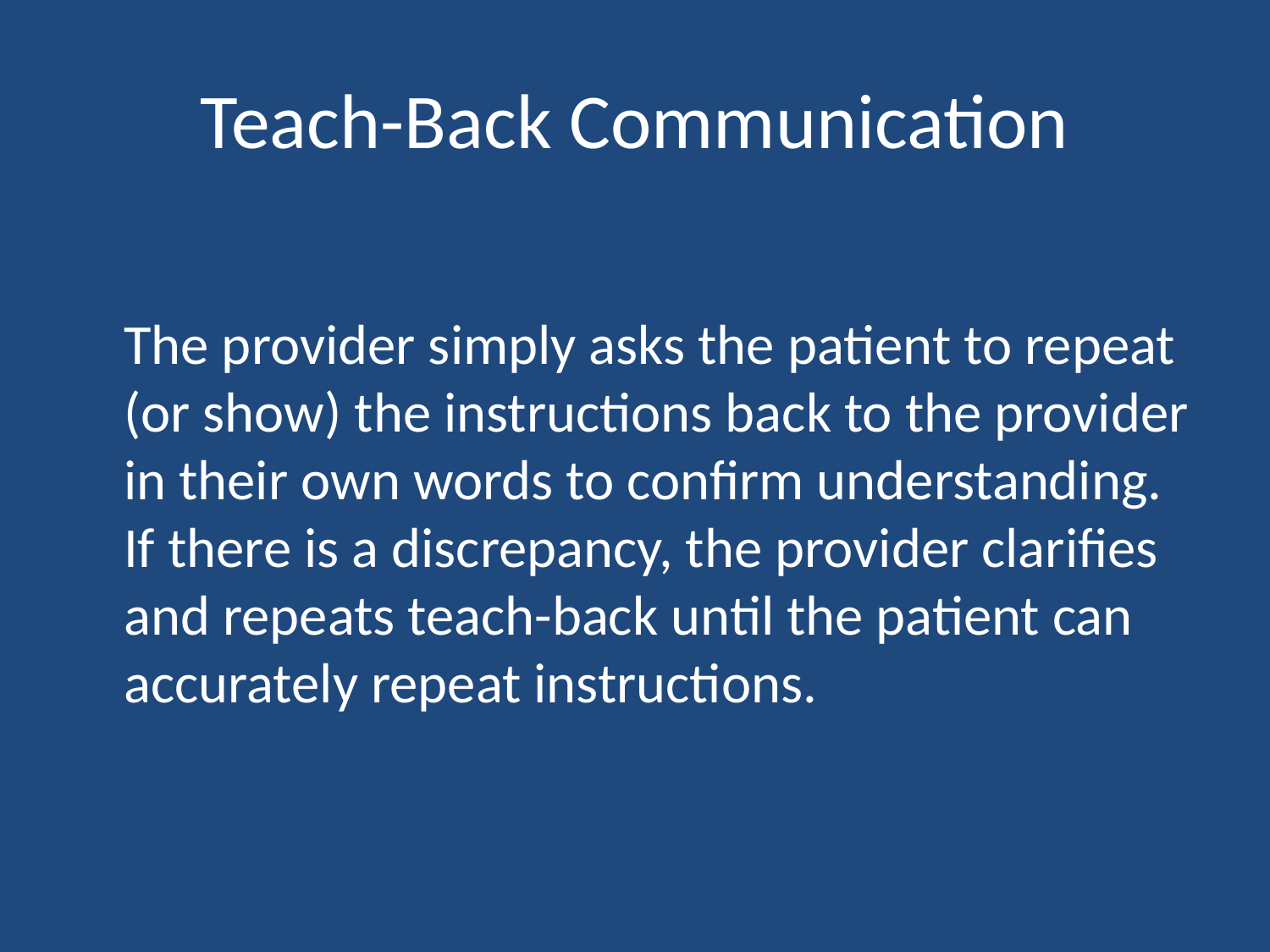

# Teach-Back Communication
	The provider simply asks the patient to repeat (or show) the instructions back to the provider in their own words to confirm understanding. If there is a discrepancy, the provider clarifies and repeats teach-back until the patient can accurately repeat instructions.

## Slide 25
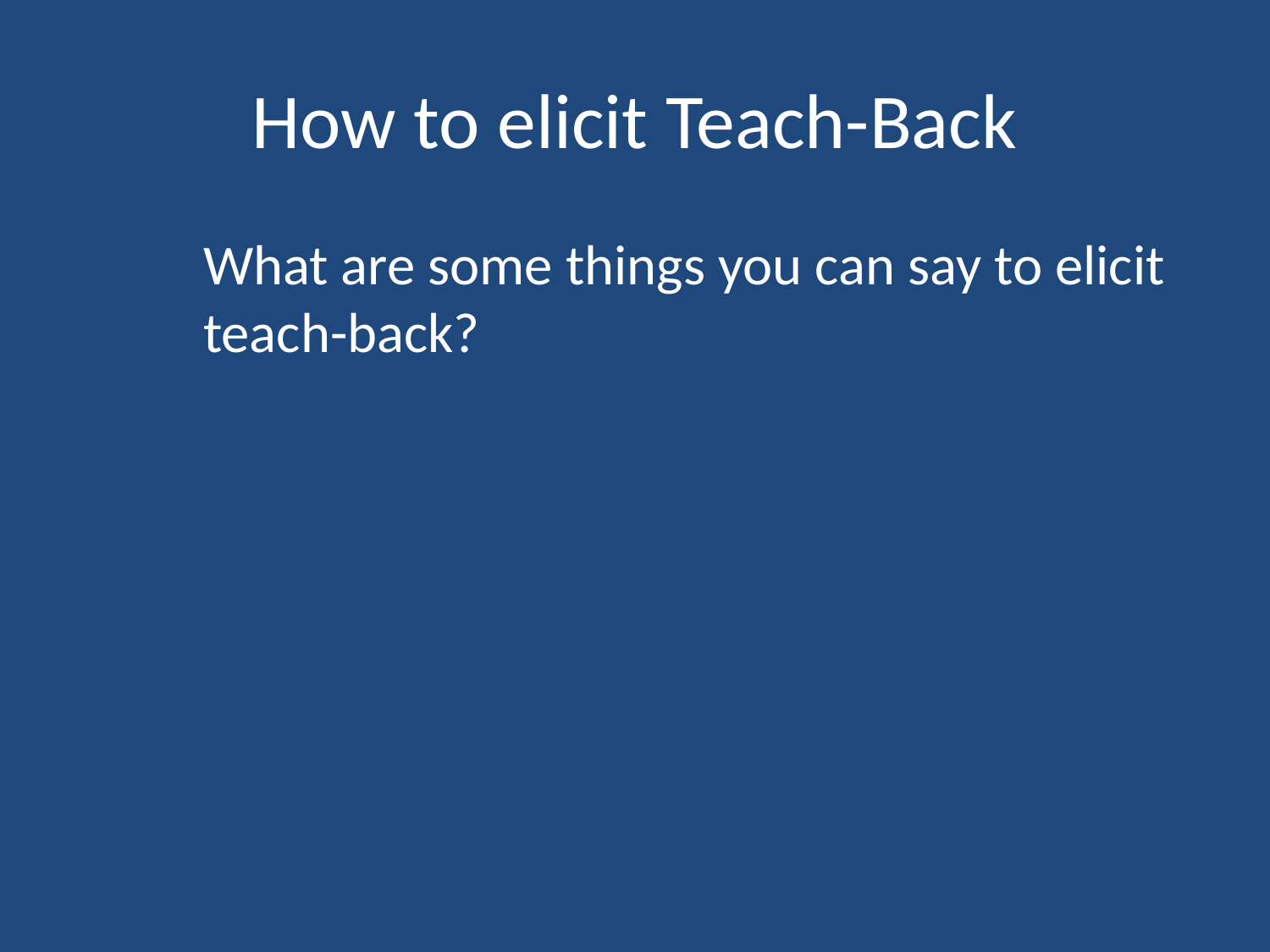

# How to elicit Teach-Back
	What are some things you can say to elicit 	teach-back?

## Slide 26
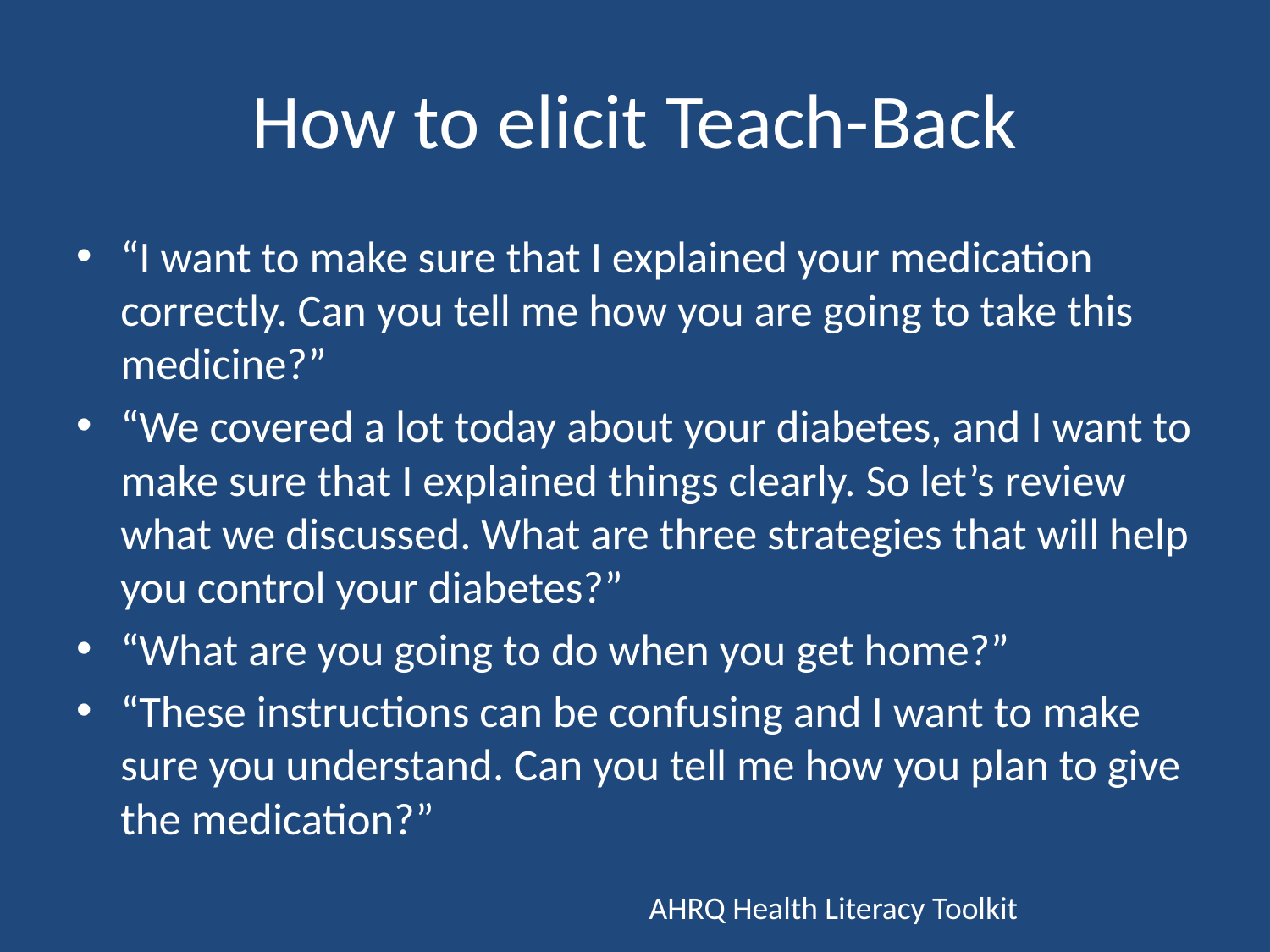

# How to elicit Teach-Back
“I want to make sure that I explained your medication correctly. Can you tell me how you are going to take this medicine?”
“We covered a lot today about your diabetes, and I want to make sure that I explained things clearly. So let’s review what we discussed. What are three strategies that will help you control your diabetes?”
“What are you going to do when you get home?”
“These instructions can be confusing and I want to make sure you understand. Can you tell me how you plan to give the medication?”
AHRQ Health Literacy Toolkit

## Slide 27
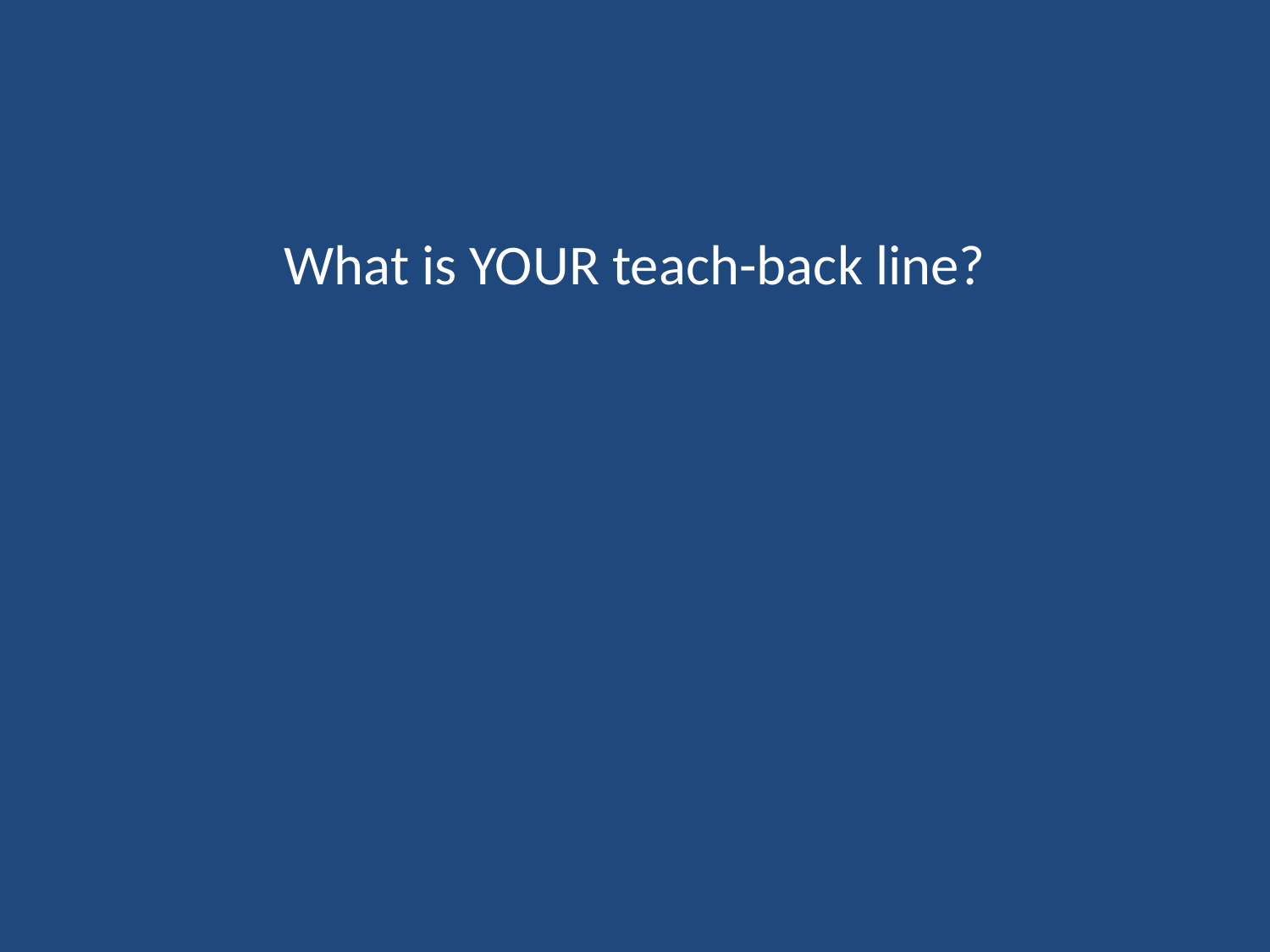

What is YOUR teach-back line?

## Slide 28
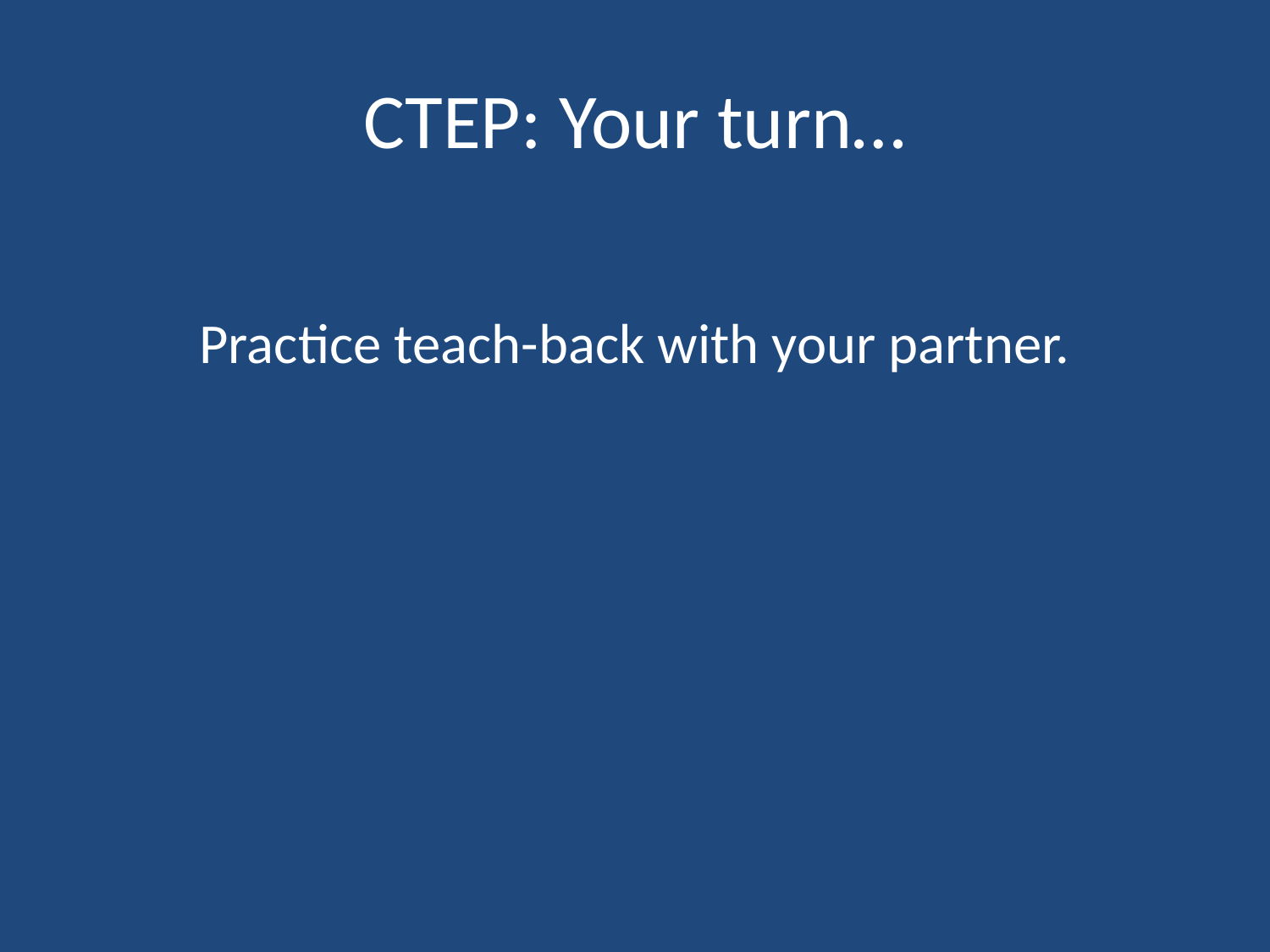

# CTEP: Your turn…
Practice teach-back with your partner.

## Slide 29
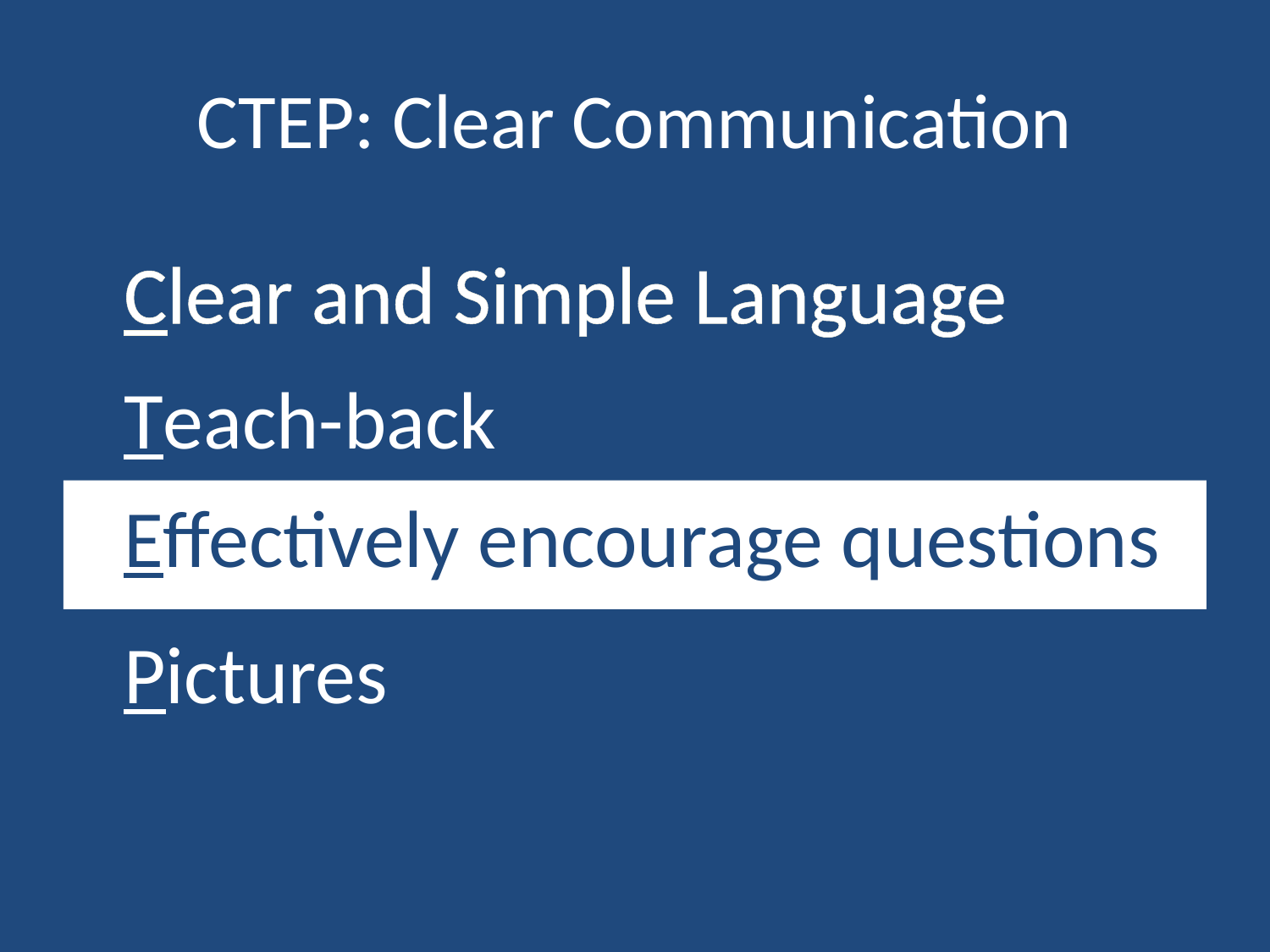

# CTEP: Clear Communication
	Clear and Simple Language
	Teach-back
	Effectively encourage questions
	Pictures

## Slide 30
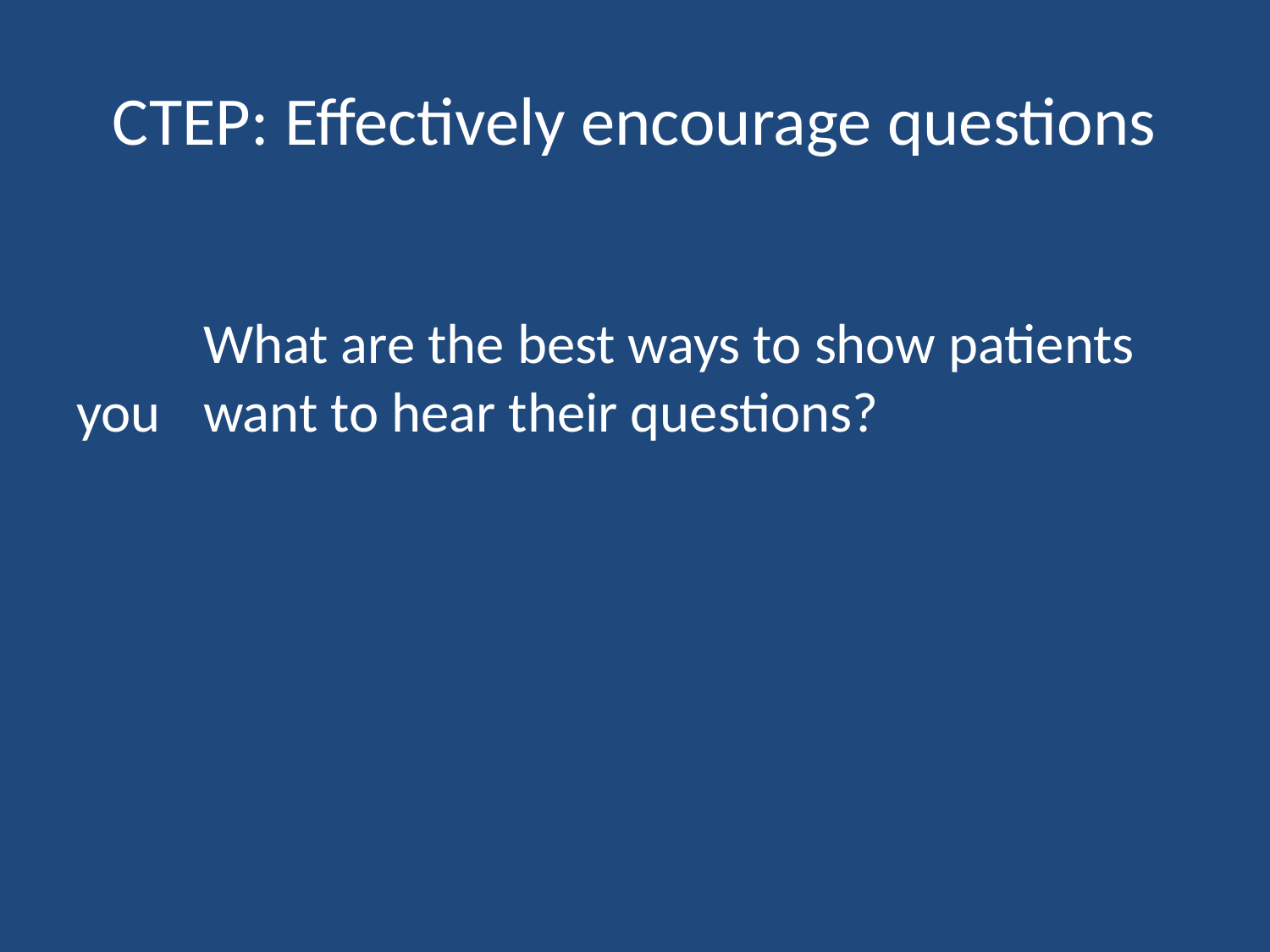

# CTEP: Effectively encourage questions
	What are the best ways to show patients you 	want to hear their questions?

## Slide 31
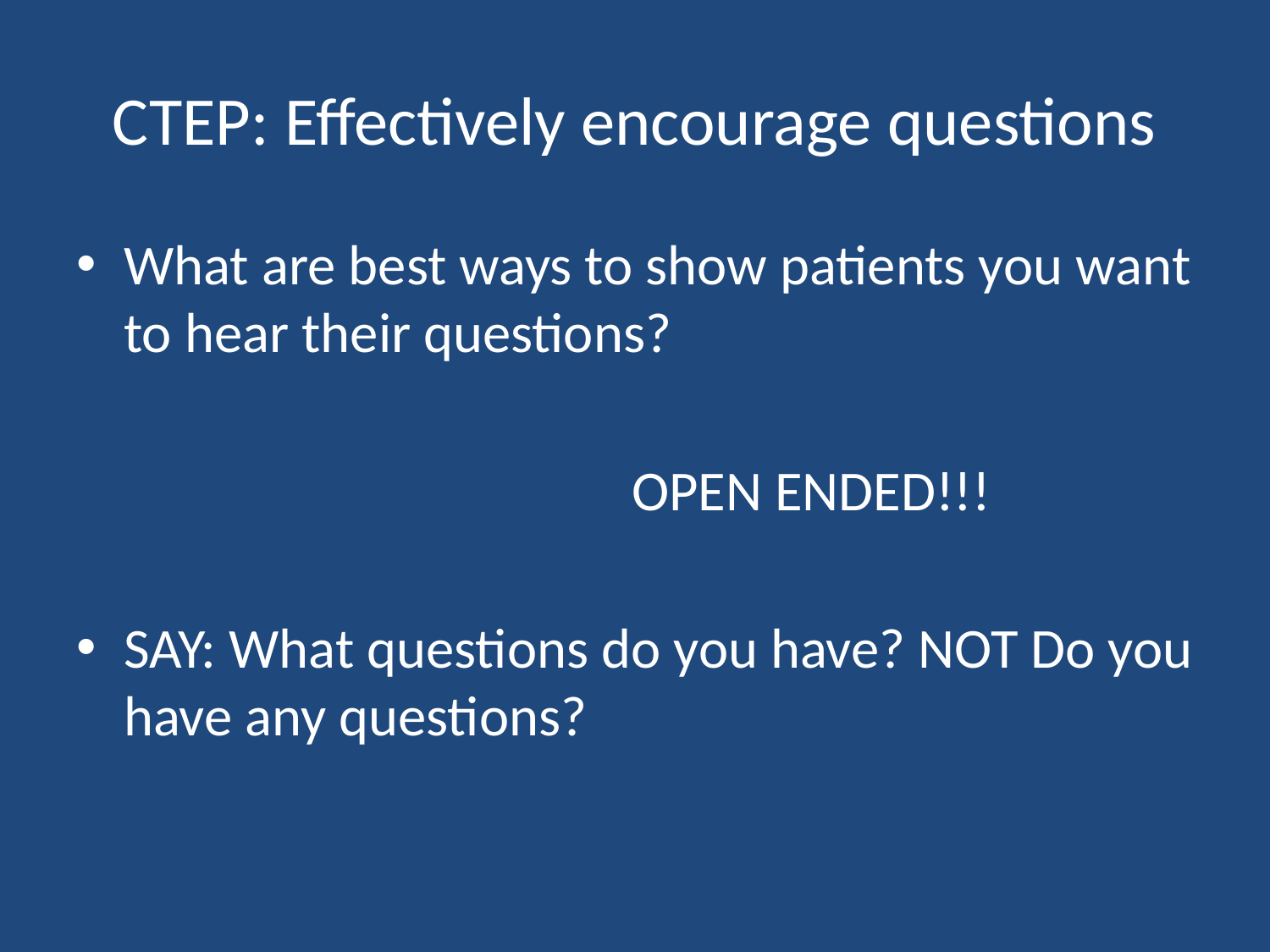

# CTEP: Effectively encourage questions
What are best ways to show patients you want to hear their questions?
					OPEN ENDED!!!
SAY: What questions do you have? NOT Do you have any questions?

## Slide 32
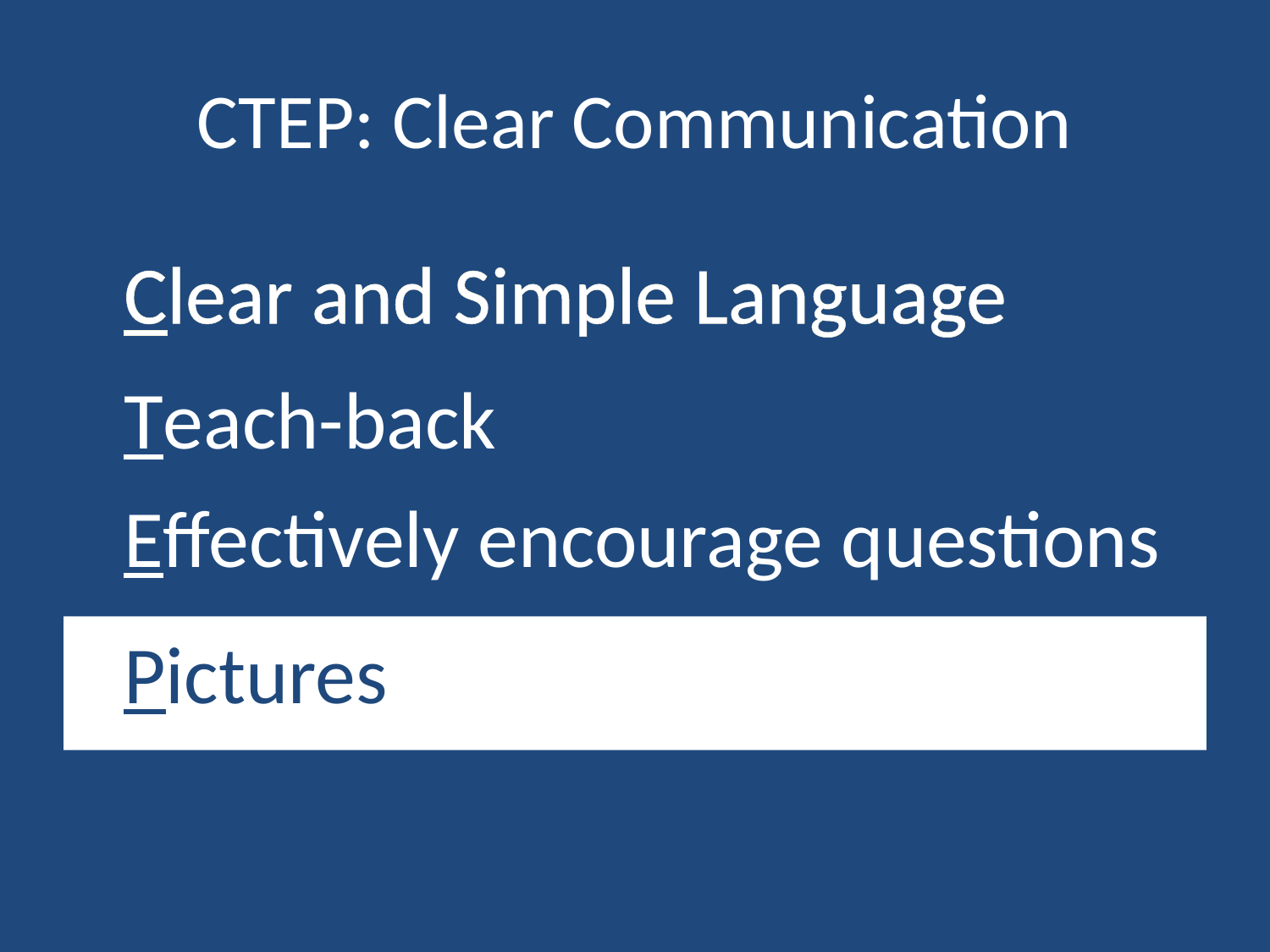

# CTEP: Clear Communication
	Clear and Simple Language
	Teach-back
	Effectively encourage questions
	Pictures

## Slide 33
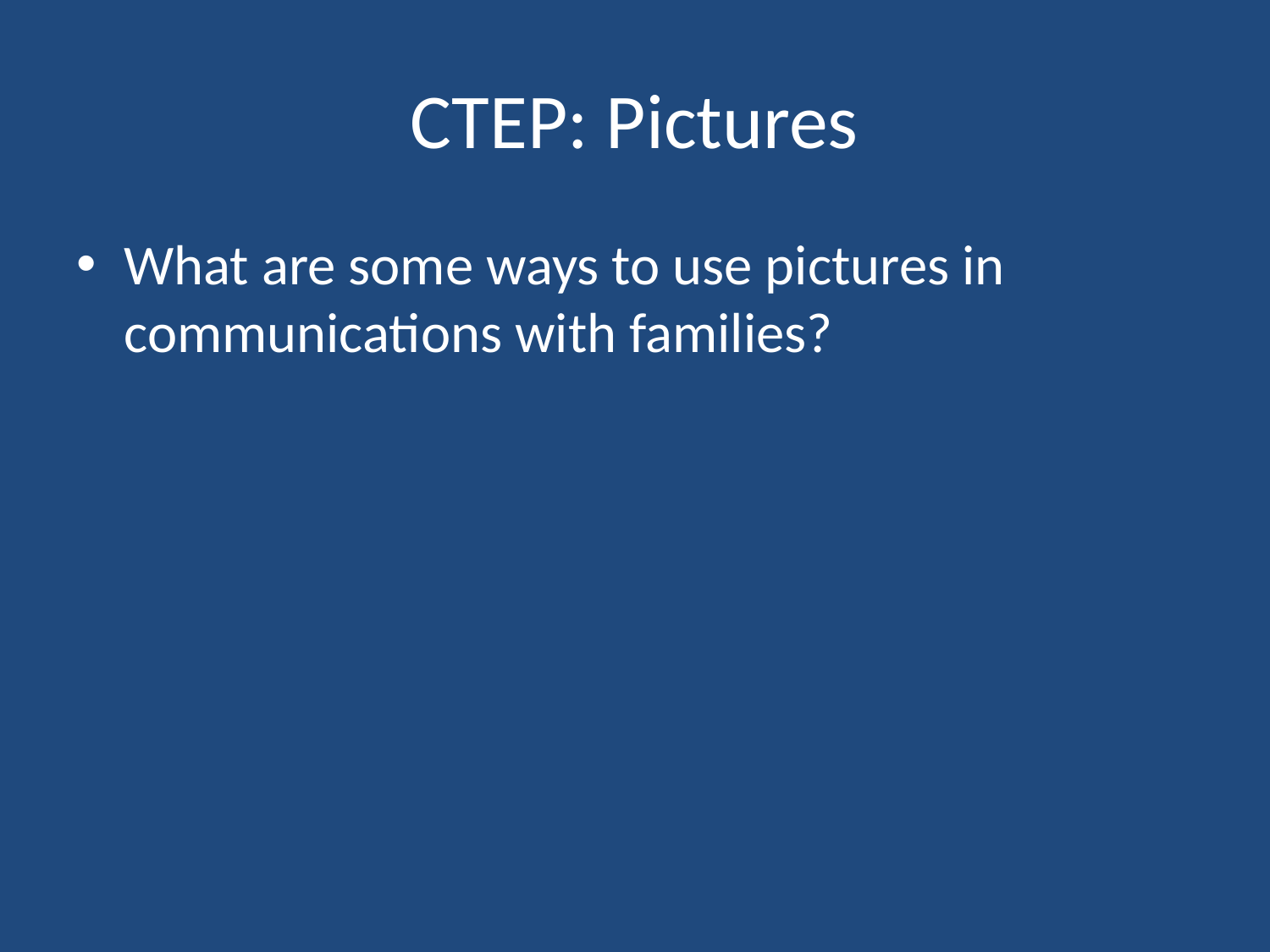

# CTEP: Pictures
What are some ways to use pictures in communications with families?

## Slide 34
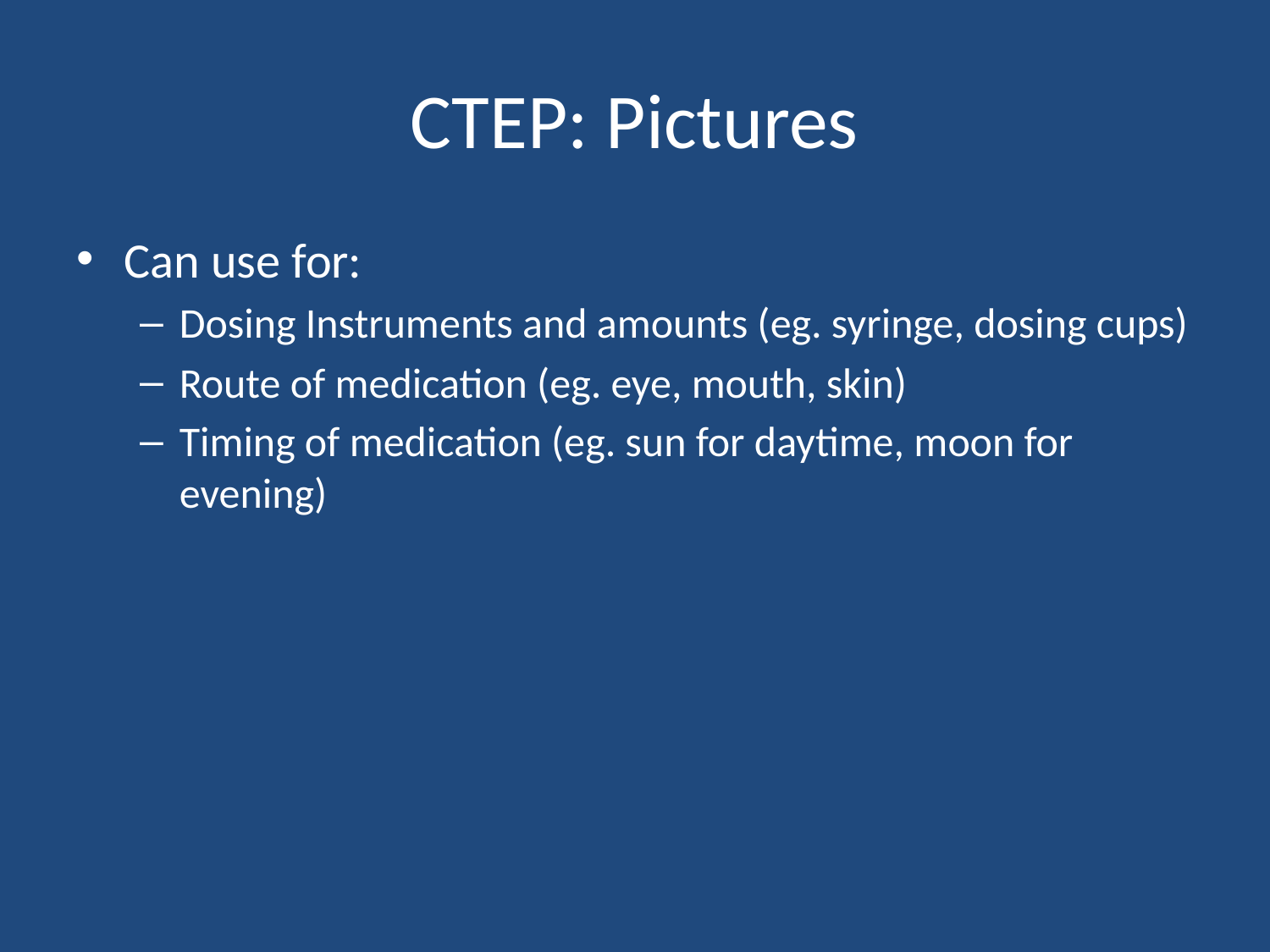

# CTEP: Pictures
Can use for:
Dosing Instruments and amounts (eg. syringe, dosing cups)
Route of medication (eg. eye, mouth, skin)
Timing of medication (eg. sun for daytime, moon for evening)

## Slide 35
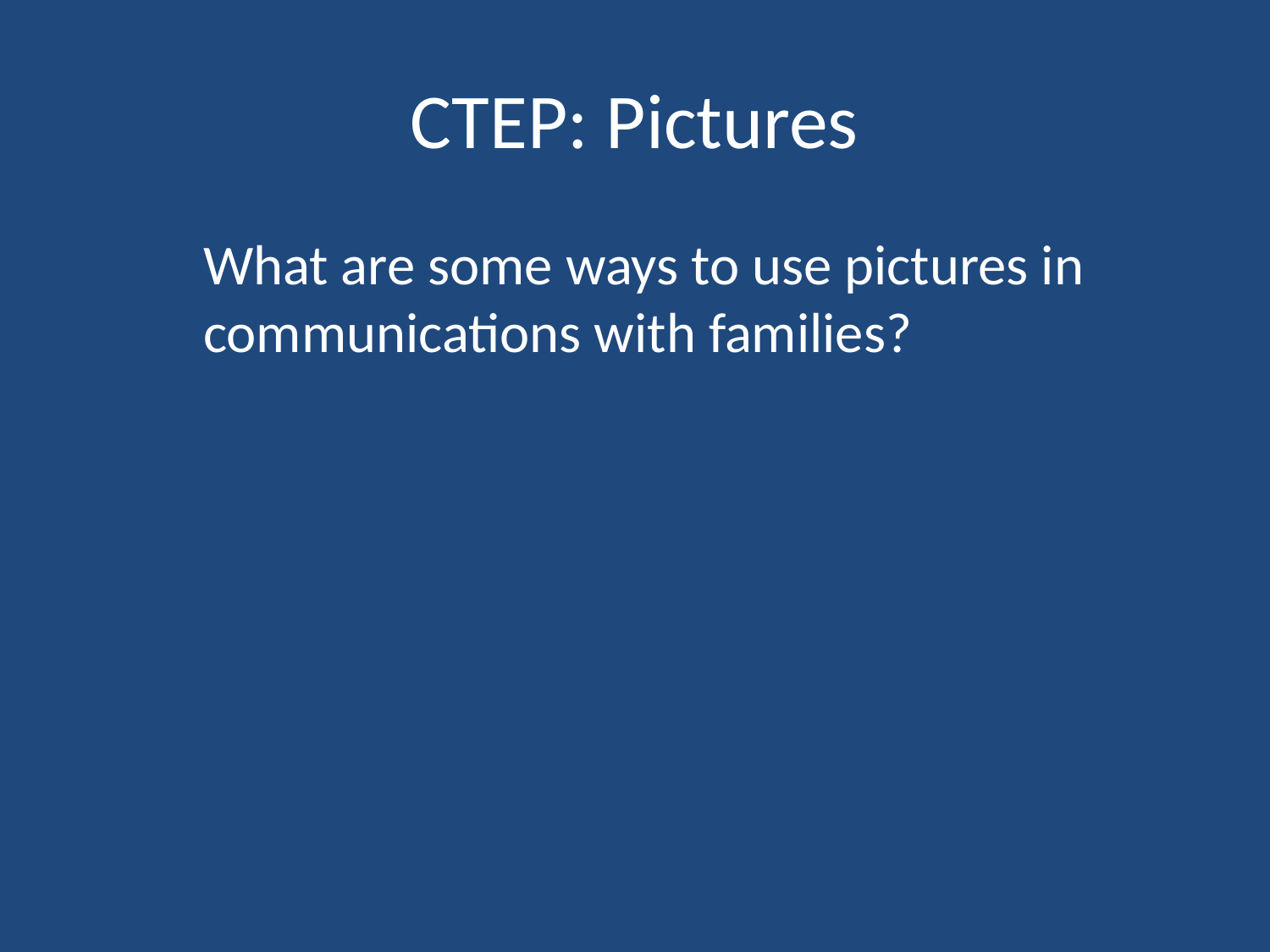

# CTEP: Pictures
	What are some ways to use pictures in 	communications with families?

## Slide 36
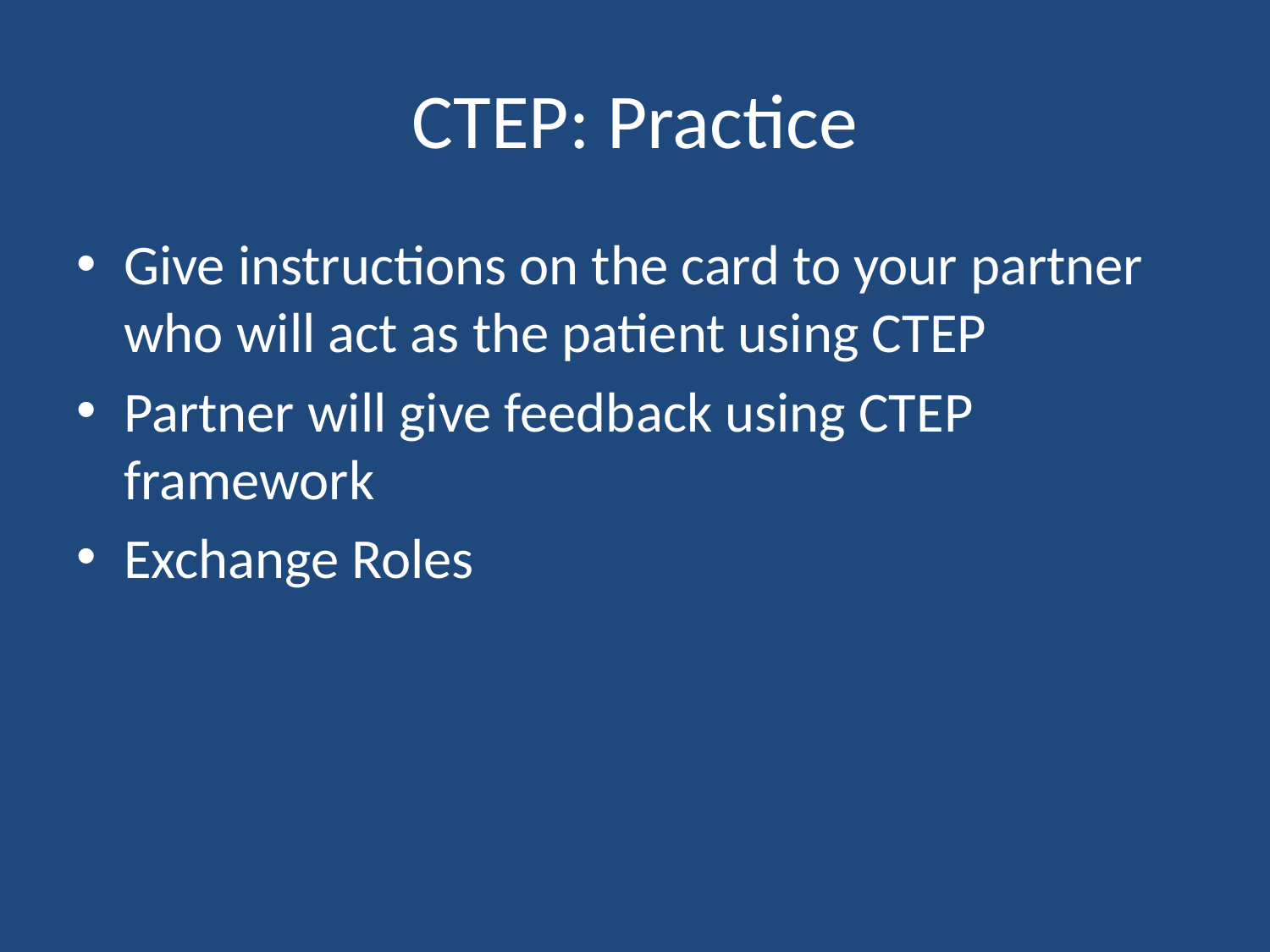

# CTEP: Practice
Give instructions on the card to your partner who will act as the patient using CTEP
Partner will give feedback using CTEP framework
Exchange Roles

## Slide 37
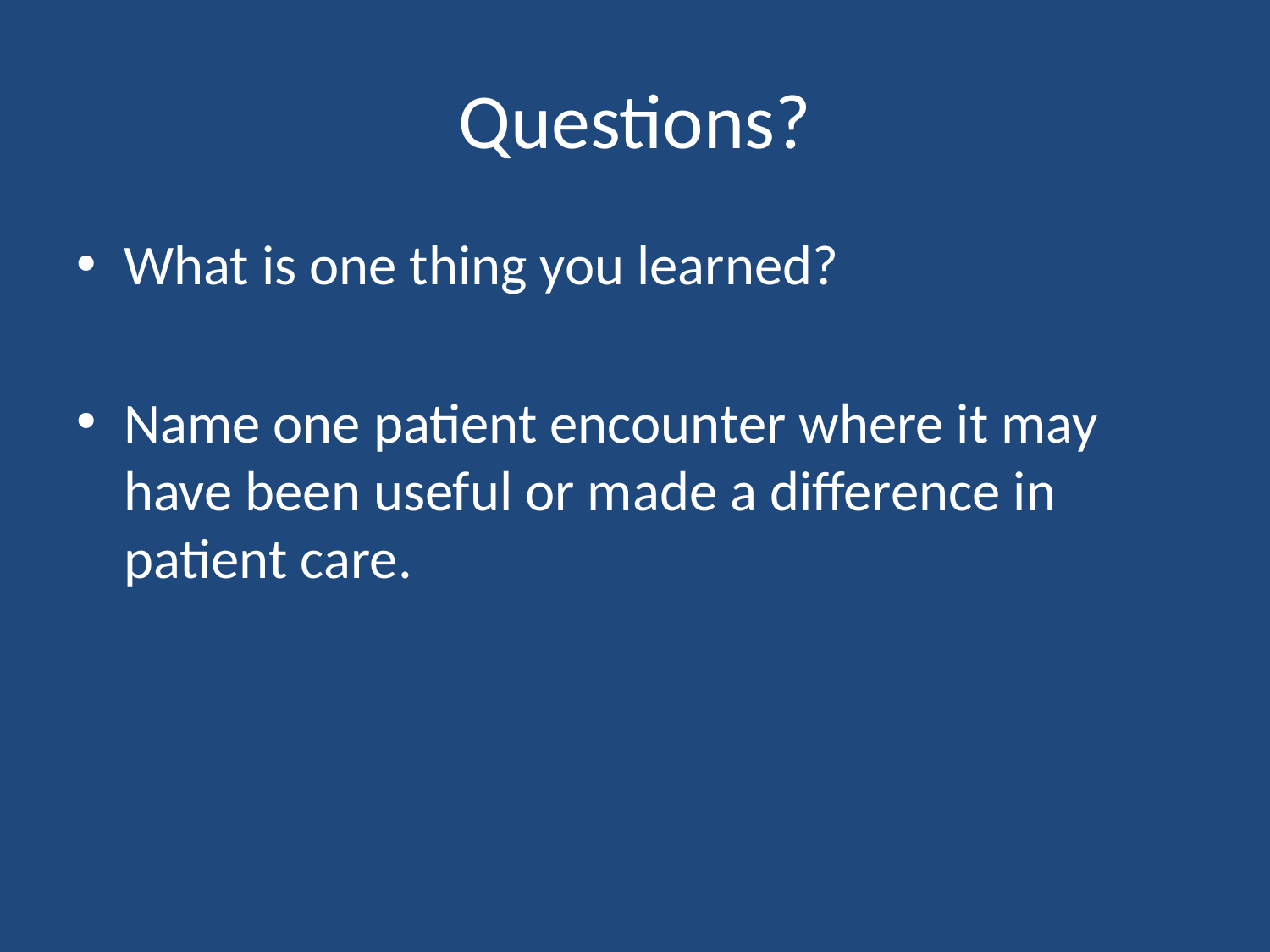

# Questions?
What is one thing you learned?
Name one patient encounter where it may have been useful or made a difference in patient care.

## Slide 38
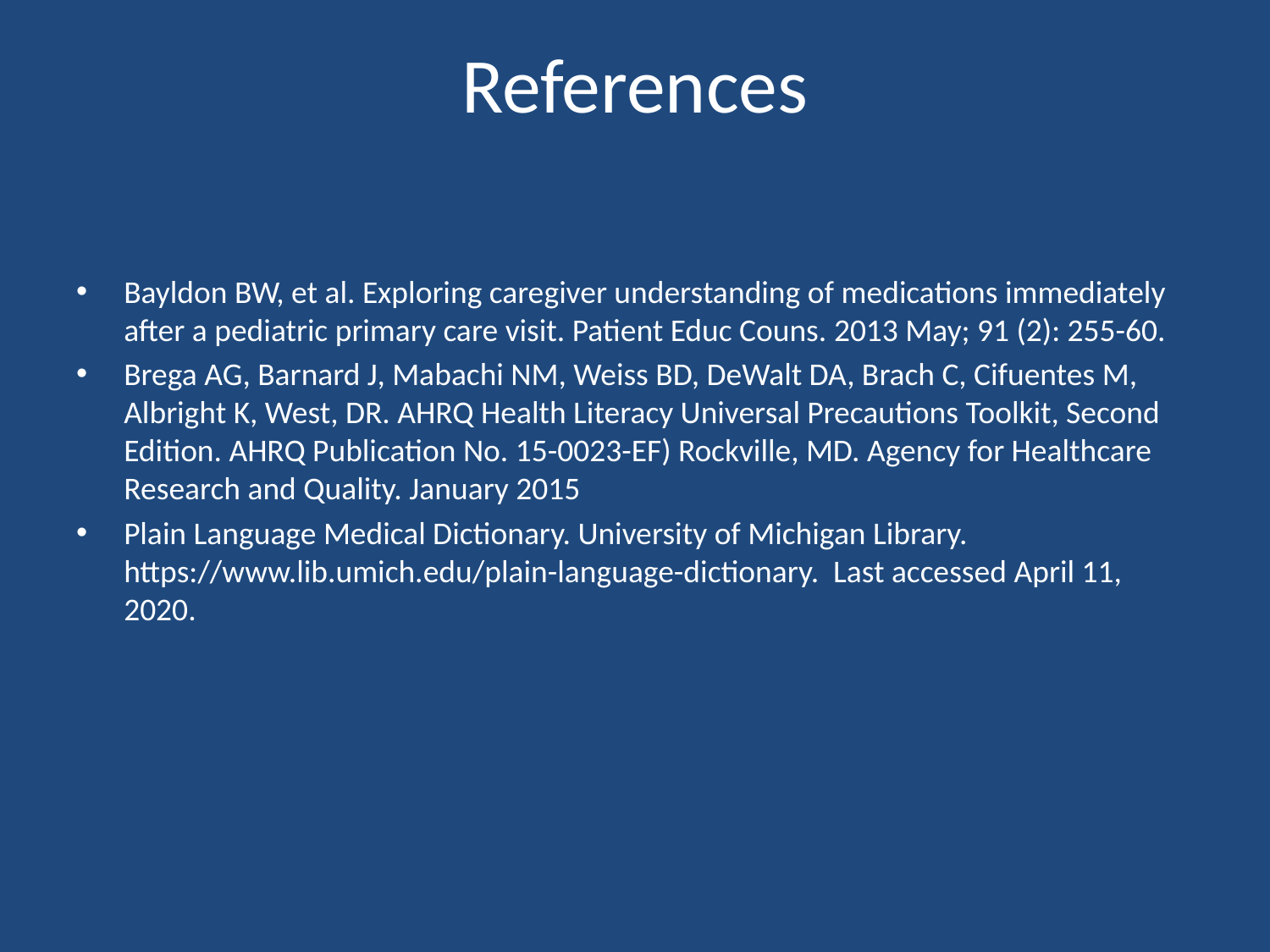

# References
Bayldon BW, et al. Exploring caregiver understanding of medications immediately after a pediatric primary care visit. Patient Educ Couns. 2013 May; 91 (2): 255-60.
Brega AG, Barnard J, Mabachi NM, Weiss BD, DeWalt DA, Brach C, Cifuentes M, Albright K, West, DR. AHRQ Health Literacy Universal Precautions Toolkit, Second Edition. AHRQ Publication No. 15-0023-EF) Rockville, MD. Agency for Healthcare Research and Quality. January 2015
Plain Language Medical Dictionary. University of Michigan Library. https://www.lib.umich.edu/plain-language-dictionary. Last accessed April 11, 2020.
