# Supplementary material for: You Can Teach Every Patient: A Health Literacy and Clear Communication Curriculum for Pediatric Clerkship Students
Source: MedEdPORTAL. 2021 Jan 22;17:11086. doi: 10.15766/mep_2374-8265.11086 (PMC7821440; doi:10.15766/mep_2374-8265.11086)
Supplement: Supplementary file 1 — HLCC Didactic PowerPoint.pptxWorkshop PowerPoint.pptxCTEP Card.docxVideo for Critique.m4vClear Language Cases Students.docxClear Language Cases Instructors Guide.docxTeach-back Cases Students.docxTeach-back Cases Instructors Guide.docxPicture Cases Students.docxPicture Cases Instructors Guide.docxCTEP Cases Students.docxCTEP Cases Instructors Guide.docxCommunication Checklist.docxStudent Survey.docx [file mep_2374-8265.11086-s001.zip › C. CTEP Card.docx]

**Appendix C. CTEP Card**

CTEP for Clear Communication

You **C**an **T**each **E**very **P**atient!

**C** = **Clear and simple language.** No medical jargon.

**T** = **Teach-back technique.** Ask patient to repeat your instructions back to you in their own words.

**E** = **Effectively encourage patient’s questions.** Act like you want to hear their questions! Say “What questions do you have?” NOT “Do you have any questions?”

**P** = **Pictures.** Use pictures or graphics whenever possible when giving patient instructions.
