# Supplementary material for: You Can Teach Every Patient: A Health Literacy and Clear Communication Curriculum for Pediatric Clerkship Students
Source: MedEdPORTAL. 2021 Jan 22;17:11086. doi: 10.15766/mep_2374-8265.11086 (PMC7821440; doi:10.15766/mep_2374-8265.11086)
Supplement: Supplementary file 1 — HLCC Didactic PowerPoint.pptxWorkshop PowerPoint.pptxCTEP Card.docxVideo for Critique.m4vClear Language Cases Students.docxClear Language Cases Instructors Guide.docxTeach-back Cases Students.docxTeach-back Cases Instructors Guide.docxPicture Cases Students.docxPicture Cases Instructors Guide.docxCTEP Cases Students.docxCTEP Cases Instructors Guide.docxCommunication Checklist.docxStudent Survey.docx [file mep_2374-8265.11086-s001.zip › E. Clear Language Cases Students.docx]

**Appendix E. Clear Language Cases: Students**

Note to instructor: You can cut out the case scenarios to pass out to students.

**Tell a family you are getting a brain and spine MRI to evaluate for multiple sclerosis.**

**Tell a family you are going to draw blood for a newborn screen.**

**Tell a patient you will do a rapid strep test and if it’s negative you will send a sample to the lab for a culture.**

**Tell a family you will do a lumbar puncture on their baby.**

**Explain to a parent you are giving their baby a vaccine today for measles.**
