# Supplementary material for: You Can Teach Every Patient: A Health Literacy and Clear Communication Curriculum for Pediatric Clerkship Students
Source: MedEdPORTAL. 2021 Jan 22;17:11086. doi: 10.15766/mep_2374-8265.11086 (PMC7821440; doi:10.15766/mep_2374-8265.11086)
Supplement: Supplementary file 1 — HLCC Didactic PowerPoint.pptxWorkshop PowerPoint.pptxCTEP Card.docxVideo for Critique.m4vClear Language Cases Students.docxClear Language Cases Instructors Guide.docxTeach-back Cases Students.docxTeach-back Cases Instructors Guide.docxPicture Cases Students.docxPicture Cases Instructors Guide.docxCTEP Cases Students.docxCTEP Cases Instructors Guide.docxCommunication Checklist.docxStudent Survey.docx [file mep_2374-8265.11086-s001.zip › F. Clear Language Cases Instructors Guide.docx]

**Appendix F. Clear Language Cases: Instructor’s Guide**

*Notes for Instructor:*

- *This exercise is done as a large group. Each student is given a case and the students take turns acting like the provider and explaining the case in plain language to the group. The group gives feedback to the student at the end of each case.*
- *Examples for sample clear language are given below, but there are endless options!*

**Case #1: Tell a family you are going to draw blood for a newborn screen.**

Sample clear language explanation: We are going to take a small amount of blood from your baby’s foot. It is to test for many different diseases that run in families. We do this test in all babies.

*(Teaching point: Use short sentences. “Run in families” is a good substitute for “genetic diseases.”)*

**Case #2: Tell a patient you will do a rapid strep test and if it’s negative you will send a sample to the lab for a culture.**

Sample clear language explanation: I am going to test your child’s throat for a bacteria, or bug, in her throat called strep. I will wipe her throat with this big Q-tip. I will do a quick test here. If we find bacteria here, I will give you antibiotics, or medicine, to treat it. If the test here does not find the bacteria, then I will send it to the lab for a better test.

*(Teaching point: Don’t ever say a test is “positive” or “negative.” Define jargon when you use any.)*

**Case #3: Tell a family you will do a lumbar puncture on their baby.**

Sample clear language explanation: We are going to do a spinal tap on your baby. We will stick a small needle in her back and take out a little liquid. We will test the liquid to find out more about your baby and why she is sick.

*(Teaching point: Use words patients may use for a procedure, such as spinal tap instead of lumbar puncture.)*

**Case #4: Tell a family you are getting a brain and spine MRI to evaluate for multiple sclerosis.**

Sample clear language explanation: We are going to get an MRI, or a picture, of your son’s brain and spine. We want to know if he has something called multiple sclerosis. This is a disease of the brain and spine where there are sores on different parts of the brain or spine.

*(Teaching point: This one is tricky! This is certainly an overly simplistic explanation of MS and I ask students if they feel that it is appropriate or not. It’s ok that everyone has a different opinion, but teach students that it is often helpful to start simple and then add layers of complexity to an explanation.)*

**Case #5: Explain to a parent you are giving their baby a vaccine today for measles.**

Sample clear language explanation: We will give your son a shot today in his leg. The shots will stop him from getting measles, which is a virus that causes fever and rash.

*(Teaching point: Use simple words, such as “stop him” instead of “prevent.” Use words your patient uses, for example if a patient uses the word “shots” instead of “vaccines”, use that word.)*
