# Supplementary material for: You Can Teach Every Patient: A Health Literacy and Clear Communication Curriculum for Pediatric Clerkship Students
Source: MedEdPORTAL. 2021 Jan 22;17:11086. doi: 10.15766/mep_2374-8265.11086 (PMC7821440; doi:10.15766/mep_2374-8265.11086)
Supplement: Supplementary file 1 — HLCC Didactic PowerPoint.pptxWorkshop PowerPoint.pptxCTEP Card.docxVideo for Critique.m4vClear Language Cases Students.docxClear Language Cases Instructors Guide.docxTeach-back Cases Students.docxTeach-back Cases Instructors Guide.docxPicture Cases Students.docxPicture Cases Instructors Guide.docxCTEP Cases Students.docxCTEP Cases Instructors Guide.docxCommunication Checklist.docxStudent Survey.docx [file mep_2374-8265.11086-s001.zip › G. Teach-back Cases Students.docx]

**Appendix G. Teach-back Cases: Students**

Note to instructor: You can cut out the case scenarios to pass out to students.

**Take azithromycin 9mls x 1 day, then 4.5mls daily x 4 more days**

**If seizure lasts longer than 5 minutes, give diazepam 5mls rectally and call 911.**

**If exposed to peanuts, take epinephrine auto-injector: Pull blue cap on end. Inject in lateral thigh through clothes and hold until clicks and then hold for an additional 10 seconds.**

**If your child has allergy symptoms, take cetirizine 5mls by mouth every night.**

**Take famotidine 2.5mls twice daily by mouth as needed for reflux.**
