# Supplementary material for: You Can Teach Every Patient: A Health Literacy and Clear Communication Curriculum for Pediatric Clerkship Students
Source: MedEdPORTAL. 2021 Jan 22;17:11086. doi: 10.15766/mep_2374-8265.11086 (PMC7821440; doi:10.15766/mep_2374-8265.11086)
Supplement: Supplementary file 1 — HLCC Didactic PowerPoint.pptxWorkshop PowerPoint.pptxCTEP Card.docxVideo for Critique.m4vClear Language Cases Students.docxClear Language Cases Instructors Guide.docxTeach-back Cases Students.docxTeach-back Cases Instructors Guide.docxPicture Cases Students.docxPicture Cases Instructors Guide.docxCTEP Cases Students.docxCTEP Cases Instructors Guide.docxCommunication Checklist.docxStudent Survey.docx [file mep_2374-8265.11086-s001.zip › H. Teach-back Cases Instructors Guide.docx]

**Appendix H. Teach-back Cases: Instructor’s Guide**

*Notes for Instructor:*

- *This is a role-play exercise. Ask students to pair off. There are five sample clinical cases (see Appendix G). The instructor selects one case for a group demonstration and then gives each pair two different cases.*
- *Ask for one pair to demonstrate the exercise for the group, after which the group will give feedback.*
- *One student will act as the “Provider,” one as the “Patient/caregiver.” The “Provider” gives the instructions on the card (in plain language) to the “Patient/caregiver” and then asks for teach-back.*
- *After the group demonstration, students break off into pairs and do the same exercise, taking turns being the “Provider.”*
- *Note: While students are encouraged to practice clear language, the focus of this exercise is eliciting teach-back.*
  - *Some suggested language for eliciting teach-back include (there are many different ways of doing this, but these are suggestions):*
    - *“That was a lot of information I just gave you and I want to make sure I was clear. Can you tell me when you plan on taking the medication?*
    - *“I know I can be confusing sometimes and I want to make sure I am clear about these instructions. Can you tell me how much of the medication you will take and how often you will take it?”*
    - *“Most of my patients find this medication hard to take, so I ask everyone to repeat the instructions back to me so I know I was clear. Can you tell me how you plan to take the medication?”*
- *Ask pairs to time how long the teach-back portion of the encounter takes. Use it as a teaching point to demonstrate that teach-back does not add significant time to the visit.*
- *Tell the students in the “Patient/caregiver” role to act like they are not expecting to be asked for teach-back and to “decrease” their health literacy level.*
- *There are numerous ways to clearly explain these cases, but examples of clear language are included below.*

**Case #1: Take azithromycin 9mls x 1 day, then 4.5mls daily x 4 more days.**

Sample clear language: Your child needs to take this medication for 5 days. The dose is 4.5mls, but the amount on the first day will be a “double dose” of the medication. Today is Monday and you will go home and give your child 9mls (a double dose) today. Then, starting tomorrow--from Tuesday until Friday, or four more days, you will give 4.5mls by mouth once a day.

**Case #2: If seizure lasts longer than 5 minutes, give diazepam 5mls rectally and call 911.**

Sample clear language: If your child’s seizure lasts for more than 5 minutes, you will need to give him/her medication. I will explain how to use it so you feel comfortable. You will use the diazepam syringe that has the medication gel in it. Lie your child on their side and spread the buttocks and insert the syringe into the rectum and use the plunger to insert the medication. Then call 911.

**Case #3: If exposed to peanuts, take epinephrine auto-injector: Pull blue cap on end. Inject in lateral thigh through clothes and hold until clicks and then hold for an additional 10 seconds.**

Sample clear language: If your child eats any peanuts, you will need to use the epinephrine auto-injector. I will explain how to use it so you feel comfortable. First take the blue cap off of the end of the pen. Then, push the needle end of the pen into the side of your child’s thigh through their clothes. Hold the pen into the thigh until it clicks, then hold it in for 10 more seconds.

**Case #4: If your child has allergy symptoms, take cetirizine 5mls by mouth every night.**

Sample clear language: If your child is having the runny, itchy nose we talked about, you will need to start giving him/her medication every night. The medication you will give is called cetirizine. It is a liquid. Every night your child will need to drink 5mls of the medication. You should give it with the cup or syringe that comes with the medication.

**Case #5: Take famotidine 2.5mls twice daily by mouth as needed for reflux.**

Sample clear language: If your child is having the stomach ache and burning feeling in their chest we talked about, you will need to start giving him/her medication every morning and night. The medication you will give is called famotidine. It is a liquid. Every morning and every night your child will need to drink 2.5mls of the medication. You should give it with the syringe that comes with the medication.
