# Supplementary material for: You Can Teach Every Patient: A Health Literacy and Clear Communication Curriculum for Pediatric Clerkship Students
Source: MedEdPORTAL. 2021 Jan 22;17:11086. doi: 10.15766/mep_2374-8265.11086 (PMC7821440; doi:10.15766/mep_2374-8265.11086)
Supplement: Supplementary file 1 — HLCC Didactic PowerPoint.pptxWorkshop PowerPoint.pptxCTEP Card.docxVideo for Critique.m4vClear Language Cases Students.docxClear Language Cases Instructors Guide.docxTeach-back Cases Students.docxTeach-back Cases Instructors Guide.docxPicture Cases Students.docxPicture Cases Instructors Guide.docxCTEP Cases Students.docxCTEP Cases Instructors Guide.docxCommunication Checklist.docxStudent Survey.docx [file mep_2374-8265.11086-s001.zip › J. Picture Cases Instructors Guide.docx]

**Appendix J. Picture Cases: Instructor’s Guide**

*Notes for Instructor:*

- *This exercise can be dropped if running low on time, but if time permits can be a fun game and nice break in the workshop.*
- *Students practice these cases as a large-group exercise/game.*
- *Students take turns drawing the instructions on the board using only pictures. The rest of the group tries to guess the instruction.*
- *Do up to three cases, time permitting.*

Case #1: Mix 1 capful of polyethylene glycol powder with 8oz water or juice and take once daily.

Case #2: Take 2 tablets by mouth every morning.

Case #3: Give your baby Vitamin D 1ml by mouth every day.
