# Supplementary material for: You Can Teach Every Patient: A Health Literacy and Clear Communication Curriculum for Pediatric Clerkship Students
Source: MedEdPORTAL. 2021 Jan 22;17:11086. doi: 10.15766/mep_2374-8265.11086 (PMC7821440; doi:10.15766/mep_2374-8265.11086)
Supplement: Supplementary file 1 — HLCC Didactic PowerPoint.pptxWorkshop PowerPoint.pptxCTEP Card.docxVideo for Critique.m4vClear Language Cases Students.docxClear Language Cases Instructors Guide.docxTeach-back Cases Students.docxTeach-back Cases Instructors Guide.docxPicture Cases Students.docxPicture Cases Instructors Guide.docxCTEP Cases Students.docxCTEP Cases Instructors Guide.docxCommunication Checklist.docxStudent Survey.docx [file mep_2374-8265.11086-s001.zip › K. CTEP Cases Students.docx]

**Appendix K. CTEP Cases: Students**

Note to instructor: You can cut out the case scenarios to pass out to students.

**Your child has a cellulitis of the arm. Give cephalexin 10 mLs every 8 hours for 7 days. Come back if there is fever or redness spreads.**

**Your baby has gastroenteritis and is at risk for dehydration. To keep her hydrated, she should drink 15mLs of electrolyte solution every 15 minutes, using a syringe. If the baby goes for longer than 6 hours without a wet diaper, you should bring her back to clinic.**

**Your child has bacterial conjunctivitis. Use ofloxacin 2 drops 4 times a day for 7 days. If not improving after 48 hours, then return to the clinic.**

**Your child had an asthma exacerbation. They will need to take albuterol 2 puffs every 4 hours for 2 days and take prednisolone 15mls daily for 4 days.**

**Your child has allergic rhinitis. They will need fluticasone: 1 squirt in each nostril every night.**
