# Supplementary material for: You Can Teach Every Patient: A Health Literacy and Clear Communication Curriculum for Pediatric Clerkship Students
Source: MedEdPORTAL. 2021 Jan 22;17:11086. doi: 10.15766/mep_2374-8265.11086 (PMC7821440; doi:10.15766/mep_2374-8265.11086)
Supplement: Supplementary file 1 — HLCC Didactic PowerPoint.pptxWorkshop PowerPoint.pptxCTEP Card.docxVideo for Critique.m4vClear Language Cases Students.docxClear Language Cases Instructors Guide.docxTeach-back Cases Students.docxTeach-back Cases Instructors Guide.docxPicture Cases Students.docxPicture Cases Instructors Guide.docxCTEP Cases Students.docxCTEP Cases Instructors Guide.docxCommunication Checklist.docxStudent Survey.docx [file mep_2374-8265.11086-s001.zip › L. CTEP Cases Instructors Guide.docx]

**Appendix L. CTEP Cases: Instructor’s Guide**

*Notes for Instructor:*

- *This is a role-play exercise that is structured like the teach-back exercise. Ask students to pair off. There are five sample clinical cases (see Appendix K). The instructor selects one case for a group demonstration and then gives each pair two different cases.*
- *Ask for one pair to demonstrate the exercise for the group, after which the group will give feedback.*
- *One student will act as the “Provider,” one as the “Patient/caregiver.” The “Provider” gives the instructions on the card to the “Patient/caregiver” using all four CTEP clear communication skills.*
- *After the group demonstration, students break off into pairs and do the same exercise, taking turns being the “Provider.”*
- *Encourage students to use a paper and pen to best incorporate pictures into their instructions.*
- *There are numerous ways to clearly explain these cases, but examples of clear language are included below.*

**Case #1: Your child has a cellulitis of the arm. Give cephalexin 10 mLs every 8 hours for 7 days. Come back if there is fever or redness spreads.**

Sample clear language: Your child has an infection on his/her arm. I will prescribe a medication, called an antibiotic, to treat it. The medication is called cephalexin. You will give it to your child using a cup or syringe. Your child will need to drink the medicine. You will need to give 10mls every 8 hours. (Try coming up with a schedule that works with the family’s schedule.) You will need to take the medication for 7 days. If your child has any fever or redness spreads, you will need to come back.

**Case #2: Your baby has gastroenteritis and is at risk for dehydration. To keep her hydrated, she should drink 15mLs of electrolyte solution every 15 minutes, using a syringe. If the baby goes for longer than 6 hours without a wet diaper, you should bring her back to clinic.**

Sample clear language: Your child has a stomach bug. She has had a lot of vomiting and diarrhea. I am worried she may be dehydrated. You will need to give her a drink called an electrolyte solution to treat her dehydration. You will use the syringe to give her 15mls every 15 minutes of the electrolyte solution. Continue this until her vomiting stops. Pay close attention to her wet diapers. If she ever goes 6 hours without any wet diapers, you should bring her back to the clinic.

**Case #3: Your child has bacterial conjunctivitis. Use ofloxacin 2 drops 4 times a day for 7 days. If not improving after 48 hours, then return to the clinic.**

Sample clear language: Your child has conjunctivitis, often called “pink eye.” It is an infection of the eye caused by a germ called a bacteria. We can treat it with antibiotic eye drops. You will give drops called ofloxacin. You will put two drops in each of his eyes 4 times a day for 7 days. A good way to remember this is to give the drops with breakfast, lunch, dinner, and before bedtime.

**Case #4: Your child had an asthma exacerbation. They will need to take albuterol 2 puffs every 4 hours for 2 days and take prednisolone 15mls daily for 4 days.**

Sample clear language: Your child had an asthma attack. He will need to take two medications. He will need to use his inhaler and take a medicine by mouth called prednisolone. The inhaler should be used with a spacer (No need to explain how to use a spacer for this exercise). You should take 2 puffs inhaled every 4 hours for the next two days (consider writing out a schedule). The prednisolone needs to be taken by mouth with a syringe. You will need to give your child the prednisolone – 15mls by mouth once a day for 4 days.

**Case #5: Your child has allergic rhinitis. They will need fluticasone: 1 squirt in each nostril every night.**

Sample clear language: Your child has allergies. The allergies are causing some irritation and swelling in her nose. We can make this better with a medication you can squirt right into her nose. The medication is called fluticasone. Here’s how you give the medication. First tilt your child’s head back slightly. Hold down one nostril while putting the tip of the spray into the other nostril. Spray the fluticasone and ask your child to breathe in deeply through their nose, then breathe out through their mouth. Repeat with second nostril.
