# Supplementary material for: You Can Teach Every Patient: A Health Literacy and Clear Communication Curriculum for Pediatric Clerkship Students
Source: MedEdPORTAL. 2021 Jan 22;17:11086. doi: 10.15766/mep_2374-8265.11086 (PMC7821440; doi:10.15766/mep_2374-8265.11086)
Supplement: Supplementary file 1 — HLCC Didactic PowerPoint.pptxWorkshop PowerPoint.pptxCTEP Card.docxVideo for Critique.m4vClear Language Cases Students.docxClear Language Cases Instructors Guide.docxTeach-back Cases Students.docxTeach-back Cases Instructors Guide.docxPicture Cases Students.docxPicture Cases Instructors Guide.docxCTEP Cases Students.docxCTEP Cases Instructors Guide.docxCommunication Checklist.docxStudent Survey.docx [file mep_2374-8265.11086-s001.zip › M. Communication Checklist.docx]

**Appendix M. Communication Checklist**

**Counseling Checklist**

To be completed by SP

**The examinee elicited the following:**

| **Fever Counseling Items*** | **Yes** | **No** |
| --- | --- | --- |
| 1. Did the student explain things in plain, non-medical language as they were counseling about fever? *Check “No” if* ***any*** *unexplained medical jargon or vague terms were used and not defined.* |  |  |
| 1. Did the student use graphics or drawings to help teach? |  |  |
| 1. Did the student check to make sure that you understood their instructions? |  |  |
| If YES, did they use:  [ ] Teach-back method (Asked you to repeat their instructions back to them in your own words.)  [ ] Open ended (e.g., “Tell me what you don’t understand about these instructions.”)  [ ] Closed ended (e.g., “Do you understand?” “Does that make sense?”) | | |
| 1. Did the student ask you if you had any questions? |  |  |
| If YES, did they use:  [ ] Open ended (e.g., “What questions do you have?”)  [ ] Closed ended (e.g., “Do you have any questions?” “Does that make sense?”)  [ ] Ambiguous (e.g., “Ok?” “Alright?” | | |

*Ref. Adapted from: Green JA et al. Addressing health literacy through clear health communication: A training program for internal medicine residents. *Patient Education and Counseling.* 2014 Apr; 95 (1): 76-82.

Comments:
