# Supplementary material for: You Can Teach Every Patient: A Health Literacy and Clear Communication Curriculum for Pediatric Clerkship Students
Source: MedEdPORTAL. 2021 Jan 22;17:11086. doi: 10.15766/mep_2374-8265.11086 (PMC7821440; doi:10.15766/mep_2374-8265.11086)
Supplement: Supplementary file 1 — HLCC Didactic PowerPoint.pptxWorkshop PowerPoint.pptxCTEP Card.docxVideo for Critique.m4vClear Language Cases Students.docxClear Language Cases Instructors Guide.docxTeach-back Cases Students.docxTeach-back Cases Instructors Guide.docxPicture Cases Students.docxPicture Cases Instructors Guide.docxCTEP Cases Students.docxCTEP Cases Instructors Guide.docxCommunication Checklist.docxStudent Survey.docx [file mep_2374-8265.11086-s001.zip › N. Student Survey.docx]

**Appendix N. Clear Communications Survey**

1. List the four components of CTEP:

C:

T:

E:

P:

1. Explain why the four components of CTEP are effective:
2. How many times have you used CTEP in the clerkship so far: (Please circle one)

0 1 2 3 4 5 6 7 8 9 ≥ 10

1. My comfort level using the CTEP clear communication skills **BEFORE** the clerkship:

(Please circle one number)

Low------------------------------Medium-------------------------------High

(1) (2) (3) (4) (5)

1. My comfort level using the CTEP clear communication skills **NOW**:

(Please circle one number)

Low------------------------------Medium-------------------------------High

(1) (2) (3) (4) (5)

(6) Prior to this clerkship, have you had any training in any of the CTEP clear communication skills? (Please circle)

YES NO
